# Future Outlook Toward Heavy‐Metal‐Free Metal Chalcogenide Nanocrystals for Optoelectronic Applications

**DOI:** 10.1002/smsc.70411

**Published:** 2026-10-14

**Authors:** Apurba Mahapatra, Ayon Das Mahapatra, Dipanjan Maity, Samrat Das Adhikari, Manoranjan Mandal, Alexander Eychmüller, Daniel Prochowicz, Fabian Paulus

**Affiliations:** ^1^ Leibniz‐Institute for Solid State and Materials Research Dresden Dresden Germany; ^2^ Physical Chemistry TU Dresden Dresden Germany; ^3^ The Hebrew University of Jerusalem, Institute of Chemistry Jerusalem Israel; ^4^ Chemistry and Physics of Materials Unit Jawaharlal Nehru Centre for Advanced Scientific Research Jakkur, Bengaluru India; ^5^ Institute of Physical Chemistry Polish Academy of Sciences Warsaw Poland; ^6^ Department of Physics Gitam Deemed to be University Bengaluru Karnataka India

**Keywords:** chalcogenide, materials science, nanocrystal, nanotechnology, optoelectronics, photodetection, photoluminescence, photovoltaics, quantum dot, surface engineering

## Abstract

Colloidal nanocrystals (NCs) represent a transformative class of materials for next‐generation optoelectronic devices, including photodetectors (PDs), photovoltaics (PVs), catalysis, and light‐emitting diodes (LEDs). Historically, research in this field has been dominated by toxic heavy‐metal‐based systems (Cd, Pb, Hg), but it is now undergoing a critical shift toward sustainability, driven by health concerns and RoHS regulations. This review provides a comprehensive overview of heavy‐metal‐free (HMF) chalcogenide NCs, including binary, ternary, and multinary systems. We analyze the fundamental principles governing their performance, specifically size‐dependent quantum confinement and compositional tuning that allow these “green” alternatives to achieve photoluminescence quantum yields exceeding 70%–90% and power conversion efficiencies of 8%–12%. This review further explores the unique structure‐property relationships of HMF chalcogenide NCs, highlighting their superior biocompatibility, low toxicity profiles, and defect‐tolerant electronic structures. Diverse applications are evaluated, ranging from broadband infrared photodetection to high‐purity color emission for QLEDs. Special emphasis is placed on the challenges of surface engineering and ligand exchange, focusing on the transition from time‐consuming layer‐by‐layer processing to efficient one‐step solution‐phase strategies. Finally, future research directions are outlined, including mitigation of ions in HMF chalcogenide NCs and development of self‐powered sensing platforms for the Internet of Things (IoT) and wearable electronics.

## Graphical abstract

Heavy‐metal‐free chalcogenide nanocrystals are a nontoxic alternative for next‐generation optoelectronic applications. This review provides a comprehensive overview of their unique optical properties, surface chemistry, and their performance in photodetectors, solar cells, light‐emitting diodes, and photocatalytic systems. We reveal the current challenges and describe the fundamental physical and chemical principles that currently limit the potential of this environmentally friendly and sustainable technology.

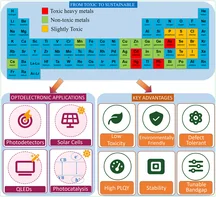

## 1 Introduction

Modern society is heavily reliant on next‐generation optoelectronic devices, including photodetectors (PDs) [1, 2, 3, 4], light‐emitting diodes (LEDs) [5, 6, 7], photovoltaic systems (PVs) [8, 9, 10], energy storage [11, 12, 13, 14], lasers [15, 16], and others. The demand for these semiconducting devices continues to grow rapidly due to increasing integration of wireless devices, the Internet of Things (IoT), and wearable and smart electronics into everyday life. Currently, most of these devices primarily rely on the established bulk semiconductors such as silicon (Si), gallium arsenide (GaAs), and indium gallium arsenide (InGaAs), which require expensive, high‐temperature fabrication and often toxic materials [17, 18]. However, further enhancement and fulfilling the increasing demand for compact and high‐performance optoelectronic components require low‐cost and easily processable alternatives. In this context, colloidal nanocrystals (NCs), commonly referred to as quantum dots (QDs) under certain conditions, have emerged as a transformative class of materials [19, 20].

Historically, this field has been dominated by heavy‐metal‐based (HMB) NCs that have been widely utilized in high‐performance applications owing to their near‐unity photoluminescence quantum yields (PLQYs) and exceptionally narrow emission spectra [21, 22, 23, 24]. Despite their excellent progress, the inherent toxicity of these heavy‐metal elements causes serious health risks as Cd, Pb, Hg, and As are known to bioaccumulate and disrupt fundamental cellular processes [25, 26, 27]. As an example, the substitution of essential biological ions by Pb^2+^ and the severe renal and bone damage caused by Cd^2+^ (notably itai–itai disease) cause serious harm to human health [27, 28]. As a result, global research has shifted toward more sustainable, safer, and environmentally‐friendly alternative materials, driven by the European Restriction of Hazardous Substances (RoHS) directive, which promotes safer commercial electronics. Heavy‐metal‐free (HMF) chalcogenide NCs, including ZnE (E = S, Se, and Te), Ag_2_E, CuInE_2_, AgInE_2_, and AgBiE_2_ chalcogenide systems, have recently emerged as compelling alternatives to conventional HMB NCs [29, 30, 31]. These materials exhibit size‐ and composition‐tunable optical properties comparable to those of Cd‐ and Pb‐based counterparts, with reported PLQYs exceeding ~70%–90% with optimized core/shell structures (e.g., CuInS_2_/ZnS and AgInS_2_/ZnS) [32, 33, 34, 35]. On the other hand, in photovoltaic applications, CuInS_2_‐ and AgBiS_2_‐based NC absorbers have delivered power conversion efficiencies (PCEs) in the range of ~ 8%–12%, highlighting their potential as safer alternatives to lead‐based systems [36, 37, 38]. Similarly, Ag_2_E‐based NCs have shown strong performance as PD_S_, with a broadband spectral response extending from the visible to the short‐wave infrared (SWIR) region and high detectivity (>10^11^ Jones) [1, 29].

Herein, we present a comprehensive overview of the chemical design, surface engineering, and optoelectronic integration of HMF‐chalcogenide NCs. Instead of solely cataloging individual studies or listing peak performance metrics, this work offers a mechanistic evaluation to elucidate the foundational structure–property–performance relationships of HMF chalcogenide NCs. We systematically evaluate their fundamental physics and surface chemistry, specifically contrasting their stoichiometry‐dependent electronic structures and ligand exchange dynamics with those of conventional HMB chalcogenide counterparts before examining their operational performance in PDs, solar cells, QLEDs, and photocatalytic systems. By analyzing how surface ligand management, defect manipulation, and heterojunction interface engineering influence charge transport and recombination dynamics, this review establishes a framework to guide the design of next‐generation, environmentally benign optoelectronic devices. Finally, we address key processing challenges and offer strategic perspectives on the commercial future of sustainable optoelectronics. Distinct from previous reviews that primarily emphasize specific material families or applications, this work uniquely integrates ligand‐specific surface‐engineering strategies with fundamental device physics and structure‐property‐performance relationships across PDs, solar cells, QLEDs, and photocatalytic systems.

## 2 Classification of HMF NCs

Historically, HMB semiconductor NCs (e.g., those containing Cd, Pb, Hg, and As) have dominated this field due to their high PLQY, narrow emission spectrum, and relatively facile synthesis [39, 40, 41]. However, the transition from hazardous HMB semiconductor NCs to less toxic or slightly toxic semiconductor NCs has been driven by the urgent demand for environmentally sustainable technologies and compliance with the European RoHS regulation [24, 29, 42]. In this review, HMF is operationally defined as semiconductor NCs formulated without restricted toxic heavy metal ions (e.g., Cd^2+^, Pb^2+^, Hg^2+^, and As^3+/5+^). Elements that are considered nontoxic (highlighted in light green in Figure 1a) or slightly toxic (marked orange in Figure 1a) under established regulatory thresholds include copper (Cu), indium (In), zinc (Zn), and similar elements. These materials provide promising alternatives for developing safer NC systems with promising optoelectronic performance [45, 46]. The landscape of HMF semiconductor NCs is broadly divided into two categories based on their chemical composition and bonding nature.

**FIGURE 1:**
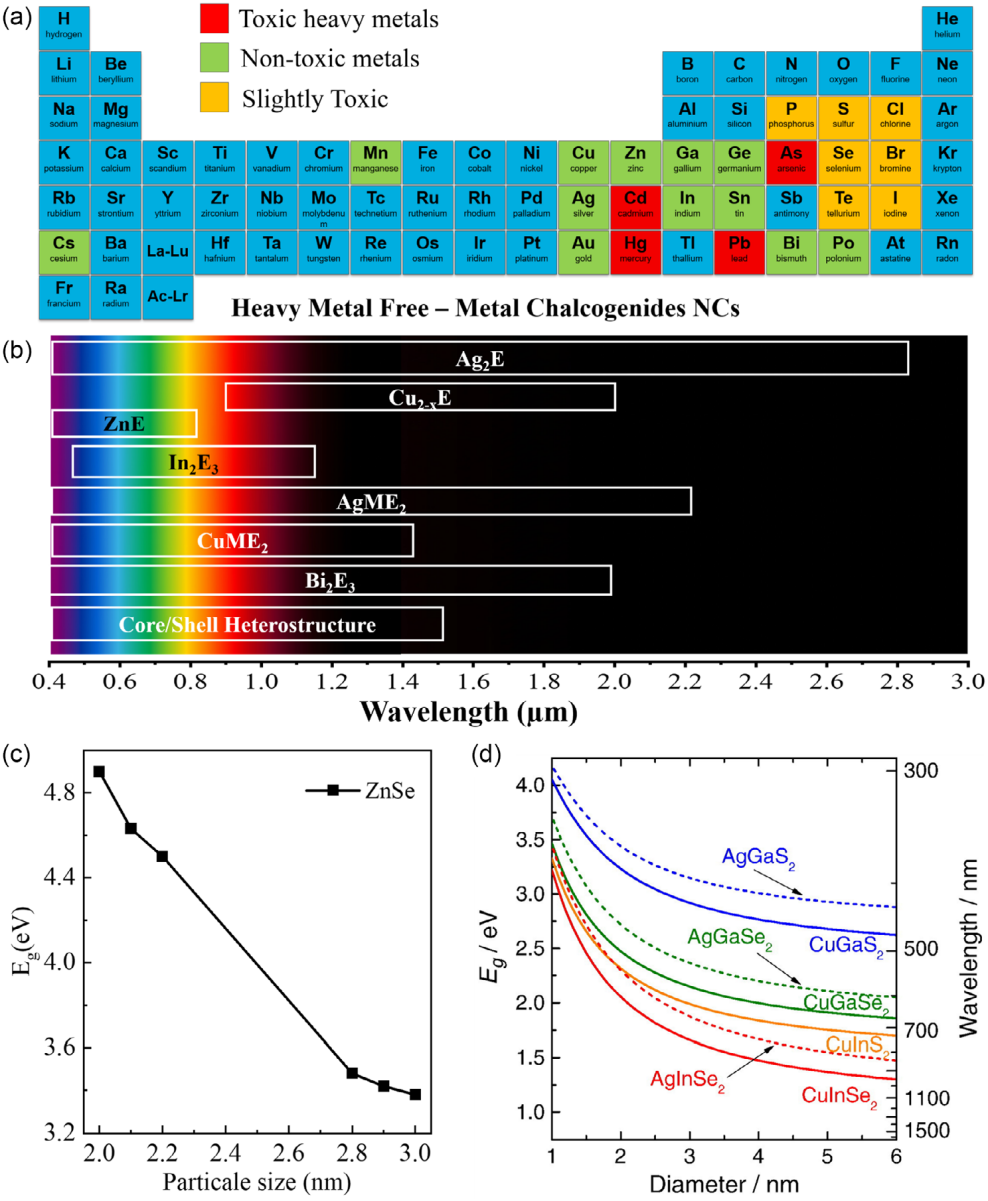
(a) Periodic table of the elements. Heavy‐metal and HMF elements are marked in red and green, respectively. (b) Spectral range of absorption of HMF chalcogenide NCs. (c) Variation of the energy bandgap as a function of particle size of ZnSe. Reproduced with permission from ref. [43]. Copyright 2025, Elsevier. (d) Evolution of the bandgap with size for various ternary chalcogenides. Reproduced with permission from ref. [44]. Copyright 2012, Elsevier.

### 2.1 Metal Chalcogenides

Chalcogenides are compounds that contain at least one chalcogen anion (such as sulfur (S), selenium (Se), or tellurium (Te)) combined with a metal cation. These materials are considered the most competitive functional alternatives to conventional HMB semiconductor NCs. Metal chalcogenide NCs can be broadly classified according to their chemical composition and structural architecture into three categories: (i) binary chalcogenides, (ii) ternary and multinary chalcogenides, and (iii) core/shell QD heterostructures.

i. Binary chalcogenides: Binary chalcogenides consist of a single metal element and a chalcogen element. Typical examples include Zinc Selenide (ZnSe), Zinc Telluride (ZnTe), and Silver Sulfide (Ag_2_S). These materials exhibit diverse electronic and optical properties that can be tuned through composition, particle size, morphology, and surface chemistry, enabling applications spanning the ultraviolet, visible, and near‐infrared (NIR) spectral regions. Their chemical stability, however, varies substantially among different compositions and should not be considered a universal characteristic of binary chalcogenides.
ii. Ternary and Multinary chalcogenides: These include more complex compounds such as I–III–VI_2_ semiconductors (e.g., CuInS_2_ and AgInS_2_), I–V–VI_2_ compounds (e.g., AgSbS_2_) [46, 47], and quaternary systems (e.g., Cu_2_ZnSnS_4_) [31, 48]. The incorporation of multiple cation species provides additional compositional degrees of freedom for tuning the electronic structure, optical response, defect chemistry, and carrier dynamics. These materials provide promising alternatives for developing HMF NC systems without significantly compromising optoelectronic performance while avoiding or reducing the use of toxic HMB elements.
iii. Core/shell QD heterostructures: Core/shell QD heterostructures represent a structural and interfacial design strategy rather than a distinct compositional class. In these architectures, a semiconductor core is surrounded by a shell of a different semiconductor material to modify surface states, carrier confinement, charge separation, chemical stability, and optical properties. Representative examples include CuInS_2_/ZnS, ZnSe/ZnS, and related systems [49, 50, 51]. Depending on the relative band‐edge alignment of the core and shell, these heterostructures can provide type‐I, quasi‐type‐II, or type‐II carrier‐confinement configurations, offering additional control over radiative recombination and charge–transfer processes.

### 2.2 Other Emerging Nonchalcogenide Semiconductor NCs

Beyond metal chalcogenides, several chemically distinct classes of semiconductor NCs have emerged as complementary materials for optoelectronic applications. Unlike chalcogenide NCs, these material families differ fundamentally in their chemical bonding, crystal structures, and electronic properties. These materials are often categorized by their position in the periodic table and their unique crystal lattices.

#### 2.2.1 III–V Semiconductors

III–V semiconductor NCs, particularly Indium Phosphide (InP), represent the most commercially advanced alternative for high‐end display technologies. Unlike the ionic nature of many chalcogenides, III–V materials possess strong covalent bonding, which provides superior structural stability. InP QDs are currently the industrial standard for cadmium‐free QLED televisions, offering narrow emission linewidths and high color purity that directly rival CdSe‐based systems [6, 52].

#### 2.2.2 Lead‐Free Perovskite NCs

Lead‐free perovskite NCs constitute a distinct class of ionic halide semiconductors with crystal structures fundamentally different from those of chalcogenides. While metal halide perovskites (e.g., FAPbX_3_, X = Cl, Br, and I; FA = Formamidinium) are traditionally often lead based [9, 53], recent research has shifted toward lead‐free variants to comply with RoHS standards. This subclass includes Bismuth‐based (Cs_3_Bi_2_X_9_, X = Cl, Br, and I), double perovskites (e.g., Cs_2_AgBiBr_6_), and tin‐based CsSnX_3_‐type (X = Br and I) perovskites, among others [54, 55]. These materials maintain the exceptional defect tolerance and high absorption coefficients characteristic of the perovskite structure while replacing toxic lead ions with safer trivalent or tetravalent metal ions but often suffer from lower stability and lower performance compared to the lead halide perovskites.

#### 2.2.3 Group IV QDs

Group IV semiconductor NCs represent a fundamentally different class based on covalently bonded elemental semiconductors. This category includes carbon dots and silicon QDs [56, 57, 58]. Carbon dots are particularly attractive due to their excellent biocompatibility and simple synthesis from organic precursors. However, these materials often exhibit broader emission spectra compared with those of metal chalcogenide QDs. On the other hand, as an earth‐abundant and nontoxic element, silicon QDs are ideal for integration with existing complementary metal–oxide‐semiconductor (CMOS) electronic platforms. Their stable photoluminescence has led to extensive investigation for biological sensing and luminescent solar concentrators.

In our review, our primary focus remains on HMF chalcogenide NCs, while these nonchalcogenide semiconductor NCs are presented as complementary material platforms that provide useful context for comparing chemical bonding, electronic structure, and optoelectronic performance.

## 3 Importance and Nature of HMF Chalcogenide NCs

HMF chalcogenide NCs have emerged as one of the most promising alternatives among nontoxic NCs owing to their tunable bandgaps, high absorption coefficients, excellent PL properties, and favorable electronic properties for optoelectronic and bioimaging applications [29, 45, 46]. Importantly, many of these nanometer‐sized NCs continue to exhibit strong quantum confinement effects, allowing precise control over their optical absorption and emission properties through manipulation of their size. In addition to the lower toxicity of the constituent metals, HMF chalcogenide NCs often provide great flexibility in compositional tuning, enabling the design of materials with tailored optical and electronic properties for specific technological applications.

### 3.1 Environmental and Health Advantages

One of the primary motivations for developing HMF chalcogenide NCs is their improved environmental and biological safety compared to that of conventional HMB NCs. Heavy metals, such as Cd, Pb, and Hg, can accumulate in living organisms and cause long‐term ecological and health damage. For example, Pb^2+^ toxicity arises primarily from ionic substitution mechanisms in which Pb^2+^ replaces essential biological ions such as Ca^2+^, Mg^2+^, and Fe^2+^, thereby disrupting numerous cellular processes, including enzyme regulation, neurotransmitter release, and intracellular signaling [59, 60]. Similarly, Hg^2+^ strongly binds to thiol groups in proteins, altering cellular structures and functions, and primarily affecting the nervous system. Chronic Hg exposure has been linked to severe neurological disorders such as Minamata disease [26]. On the other hand, Cd^2+^ also presents considerable health risks despite its relatively shorter industrial history. Cd tends to accumulate in biological tissues, particularly in the kidneys, where excessive concentrations can inhibit essential metalloenzymes and disrupt cellular metabolism [27, 28]. Long‐term exposure to Cd^2+^ has been associated with severe bone disorders such as Itai–itai disease, which results from impaired calcium metabolism and bone demineralization. Furthermore, Cd‐based NCs, such as CdSe, can release toxic Cd^2+^ ions under oxidative or ultraviolet conditions, leading to decreased cellular viability and the generation of reactive oxygen species that cause oxidative stress and cellular damage [27, 28].

In addition to their direct toxicity, heavy metals can persist in the environment and enter food chains through air, water, and soil transport, ultimately posing long‐term risks to ecosystems and human health. Consequently, international regulations such as the European RoHS directive limit the allowable concentrations of hazardous heavy metals in electronic materials. In contrast, elements commonly used in HMF chalcogenides, such as Zn‐, Cu‐, Ag‐, Bi‐, and In‐based chalcogenides, generally exhibit lower toxicity and higher biological tolerance [61, 62, 63]. This significantly reduces the toxicity and minimizes the environmental risks associated with large‐scale production and disposal of such nanomaterials and electronic devices. Moreover, the limited release of metal ions from certain systems, such as silver chalcogenides, further enhances their suitability for biological and biomedical applications. As a result, nontoxic metal chalcogenides are increasingly considered key materials for the development of environmentally sustainable nanotechnology and next‐generation optoelectronic devices.

### 3.2 Biocompatibility and Toxicity Profile

The biocompatibility and toxicity profile of NCs are primarily governed by their surface properties, colloidal stability, and interactions with biological environments rather than their core composition. Upon entering biological media, NCs rapidly interact with proteins to form a “protein corona,” a dynamic coating that dictates cellular uptake, immune recognition, and overall biodistribution [45, 64, 65]. On the other hand, the toxicity of these systems is primarily driven by oxidative stress mechanisms, where surface defects or incomplete passivation in materials like ZnS or CuInS_2_ facilitate charge‐transfer processes that generate reactive oxygen species, potentially leading to lipid peroxidation and DNA damage [66, 67]. A key advantage of HMF chalcogenide NCs is their low acute systemic toxicity compared to cadmium‐based counterparts; however, they can still induce localized inflammatory responses and lysosomal membrane permeabilization if the protective shell degrades [68, 69]. This reactivity is highly size‐dependent as smaller particles with higher surface‐to‐volume ratios exhibit increased electronic activity and cytotoxicity. Once internalized via endocytosis, NCs often localize within acidic lysosomes, where partial degradation of the NCs under these conditions can influence long‐term biostability and clearance, which occurs through size‐dependent renal or hepatobiliary pathways. HMF chalcogenide NCs could offer improved chemical robustness. To enhance this, various surface passivation strategies have been developed to improve their chemical stability and safety further. The formation of core–shell structures, such as CuInS_2_/ZnS, represents one of the most effective approaches for suppressing surface defects and minimizing electron‐transfer reactions that lead to reactive oxygen species formation [70]. Such structural heterostructures not only enhance the PL efficiency but also significantly improve the overall robustness of HMF chalcogenide NCs in physiological environments. In summary, the careful design of surface chemistry, including the use of multifunctional coatings like zwitterionic ligands or attachement of polyethylene glycols (PEGylation), can suppress defect‐related electronic states and reduce nonspecific adsorption, thereby enhancing the safe application of these NCs. However, the exact interaction of HMF chalcogenide NCs in ecological and biological systems to evaluate their toxicity requires further investigation.

### 3.3 Electronic Structure, Bandgap Tunability, and Defect States

Bandgap tunability in metal chalcogenides arises from both quantum confinement and compositional effects [71, 72, 73, 74]. In NCs, quantum confinement arises when the NCs size approaches or becomes smaller than the exciton Bohr radius [75, 76, 77]. This confinement increases the kinetic energy of carriers and elevates the band‐edge energy difference between the valence and conduction band (CB) edges, resulting in a blueshift relative to the optical bandgap of bulk material. The size‐dependent bandgap (*E*_gap, QD_) is traditionally described by the Brus equation, building on the foundational work by the Efros brothers [74, 75, 78, 79]. The size‐dependent electronic structure of ideal, spherical semiconductor QDs is traditionally modeled combining the bulk bandgap (*E*_gap, bulk_) with confinement and Coulomb interaction terms:

(1) $$E_{\mathrm{gap},\mathrm{QD}}=E_{\mathrm{gap},\mathrm{bulk}}+\frac{\hbar^{2}\pi^{2}}{2r^{2}}\left(\frac{1}{m_{e}^{*}}+\frac{1}{m_{h}^{*}}\right)-\frac{1.8e^{2}}{4\pi \varepsilon_{0}\varepsilon_{r}r}$$

where *r* is the QD radius, $m_{e}^{*}$, and $m_{h}^{*}$ are the effective masses of electrons and holes, and the final term represents the Coulomb attraction between the electron and hole [75]. While the Brus equation provides a useful first‐order description of size‐dependent bandgap evolution arising from quantum confinement, its applicability is limited for multinary chalcogenide nanocrystals with compositional disorder, anisotropic morphologies, and high defect densities. In such systems, alloy fluctuations, surface states, and defect‐induced localized electronic states can substantially modify the optical bandgap, requiring more comprehensive theoretical approaches beyond the effective‐mass approximation. As the QD size decreases, quantum confinement becomes stronger, resulting in an increase in the bandgap energy and a corresponding shift of optical absorption and emission toward shorter wavelengths. This phenomenon has been extensively exploited in traditional Cd‐ and Pb‐based QDs and remains equally important in the design of HMF chalcogenide NCs. As an example, ZnS QDs typically show optical bandgaps in the range of ~3.6–3.9 eV depending on their size, while ZnSe NCs exhibit bandgaps tunable from ~2.8 to above 3.1 eV [43, 80, 81].

Beyond size, doping and defect engineering provide additional pathways for spectral control. For example, Cu‐doped ZnSe NCs exhibit a broad emission (470–550 nm) that shifts with the host lattice size, while Mn‐doped ZnSe structures allow for tunable PL (575–595 nm) controlled by shell thickness [30, 82]. Mn doping in chalcogenide QDs has been studied extensively due to their distinct emission feature originating from the spin‐flipped d–d transition [82]. While intrinsic defects like vacancies or surface traps are often responsible for nonradiative losses, controlled defect engineering can also be exploited in ternary chalcogenides (such as AgInSe_2_ and CuInSe_2_) to achieve broadband emission or white‐light generation. These ternary systems possess small bandgaps in their bulk form, *E*_gap,bulk_ 1.2 and 1.04 eV, respectively, enabling size‐dependent absorption and emission wavelengths extending from 750 nm to beyond 1200 nm. The nature of optical transitions differs significantly across these HMF systems. In Zn‐based chalcogenides, PL is typically dominated by narrow near‐band‐edge excitonic recombination. In contrast, Ag‐ and Cu‐based I–III–VI_2_ NCs frequently exhibit broad PL spectra with large Stokes shifts, attributed to donor–acceptor pair (DAP) recombination or band‐tail states arising from cation disorder. However, not all Ag‐based chalcogenide NCs exhibit efficient PL. For instance, AgBiS_2_ NCs often show weak or nearly absent PL emission despite their strong optical absorption, a phenomenon associated with their highly disordered crystal structure, abundant defect states, and ultrafast nonradiative recombination pathways [83, 84]. This “no PL” behavior highlights the difference between materials optimized for light absorption and carrier extraction versus those designed for efficient radiative emission. Collectively, the high absorption coefficients and defect‐tolerant electronic structures of HMF chalcogenides position them as environmentally benign building blocks for next‐generation photovoltaics (PVs) and sensors.

## 4 Applications

Based on their tunable sizes, compositions, and defect‐tolerant band structures, HMF chalcogenide NCs serve as versatile light‐absorbing and emitting materials in several key technological areas. These materials are primarily utilized in PDs and solar cells for efficient light harvesting across the visible and even toward the infrared spectra, as well as in catalysis and LEDs, where their unique surface states and radiative recombination pathways are leveraged.

### 4.1 PDs

Semiconductor PDs are commonly categorized into three primary device architectures: photoconductors, photodiodes, and phototransistors. Different types of detectors and their corresponding generalized characteristics are shown in Figure 2.

**FIGURE 2:**
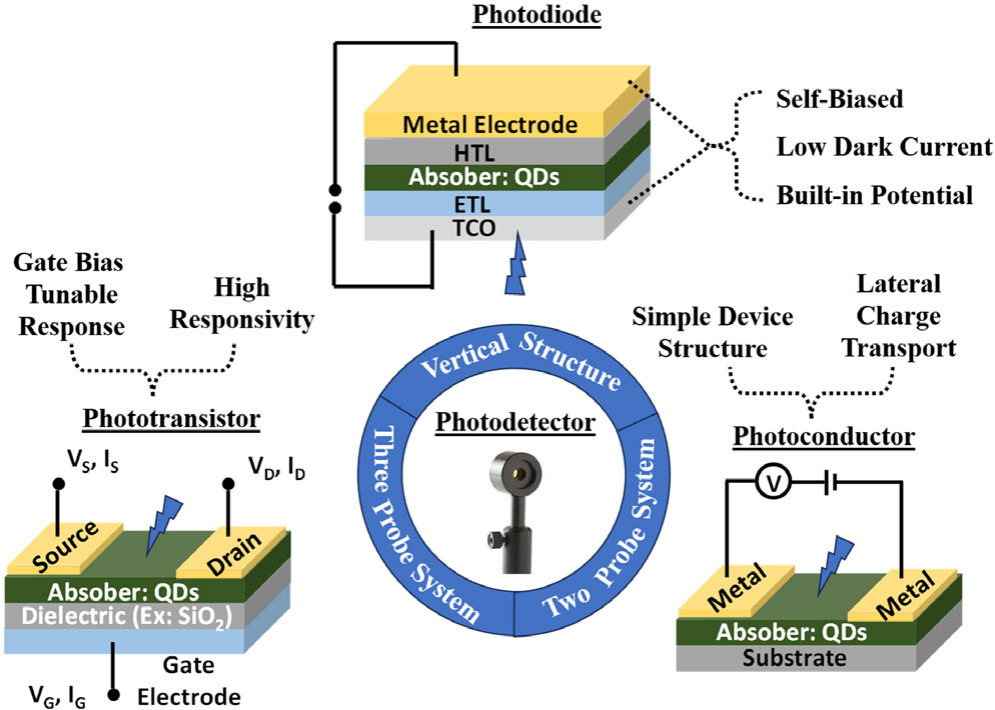
Device structures and corresponding generalized characteristics of photodetectors.

• Photoconductors operate through a light‐induced change in conductivity under an applied bias. Photogenerated carriers increase the channel conductance, and if carrier lifetimes exceed transit times, an internal gain can be achieved through multiple carrier recirculation events. While photoconductors often exhibit high responsivity, their performance can be limited by slow response times and trap‐assisted recombination.
• Photodiodes rely on a built‐in electric field at p–n and p–i–n junctions to separate and extract photogenerated carriers. Such built‐in electric fields are typically generated by the electron‐transporting layer (ETL) and hole‐transporting layer (HTL) or asymmetric contact materials. The internal electric field enables efficient charge separation and collection in the corresponding electrodes even at low or zero external bias, leading to lower noise and faster response compared to photoconductors. Photodiodes are widely employed in high‐speed and imaging applications due to their stability and linearity.
• Phototransistors integrate optical absorption with transistor amplification. In these devices, photogenerated carriers modulate the conductivity of a gate‐controlled channel, allowing direct signal amplification through the transistor functionality. This configuration offers high sensitivity and tunable gain but may involve more complex device designs and often requires optimization with respect to the applied biases.

#### 4.1.1 PD Parameters

PDs function as interfaces between optical signals and electronic systems, enabling light stimuli to be converted into detectable electrical responses [85]. Their working principle involves a series of charge generation and transport processes. Upon illumination, photons with sufficient energy equal to or greater than the semiconductor bandgap excite electrons creating electron–hole pairs within the active layer. These photogenerated charge carriers modify electrical properties, such as conductivity, current, or voltage. These photogenerated carriers are then ideally separated and driven by internal or applied electric fields, resulting in the formation of a photocurrent. This current is subsequently collected and interpreted by an external circuit, allowing the incident light to be accurately sensed and quantified. PDs are key components in modern technology, enabling efficient conversion of light into electrical signals for applications ranging from communication and sensing to medical diagnostics and guidance systems. Their role is rapidly expanding into emerging fields such as autonomous vehicles, intelligent safety systems, and advanced health monitoring.

The PD performance depends not only on light absorption but also on efficient carrier separation, transport, and minimized recombination, with interfaces playing a crucial role in reducing losses and noise. Key metrics, such as sensitivity (*S*), responsivity (*R* *_λ_*), specific detectivity (D*), external quantum efficiency (EQE), response speed (*τ*), signal‐to‐noise ratio (SNR), linear dynamic range (LDR), and stability, are governed by intrinsic material properties, device architecture, and applied bias conditions, which are elaborated in the equations below.

The most basic parameter, sensitivity *S,* for a PD is defined as a unitless parameter.

(2) $$S=\frac{I_{\mathrm{ph}}}{I_{d}}$$

Here, *I*_d_ and *I*_ph_ define the current values corresponding to dark and under‐light conditions, respectively. The responsivity (*R* *_λ_*) is a wavelength (*λ*)‐dependent parameter describing the gain of a detector based on illumination intensity and incident illumination power density (*P*), which is derived as

(3) $$R_{\text{λ}}=\frac{J_{\mathrm{ph}}-J_{d}}{P}$$

*R* *_λ_* is usually expressed in SI units of A/W, whereas *J*_ph_ and *J*_d_ define the photo and dark current densities. The EQE is a synonym for the efficiency with which the incident photons are converted to electrons and is expressed as:

(4) $$\mathrm{EQE}=\frac{R_{\text{λ}}}{\text{λ}}\frac{hc}{q}=\frac{h\text{ν}R_{\text{λ}}}{q}$$

where *c* is the velocity of light, *ν* is the frequency of the optical signal, and *h* is Planck's constant.

The specific detectivity D* of a photodetector is a figure of merit taking the noise current (*i*_n_) of the device into account and is represented as [86]:

(5) $$D^{*}=\frac{R_{\text{λ}}}{\sqrt{2qJ_{d}}}=\frac{\sqrt{A_{\mathrm{PD}}\cdot \text{Δ}f}}{\mathrm{NEP}}∼\frac{R_{\text{λ}}\sqrt{A_{\mathrm{PD}}\cdot \text{Δ}f}}{i_{n}}$$

where *q* is the charge of an electron, *A*_PD_ is the active area of the device, and Δ*f* is the bandwidth, NEP is the noise equivalent power and D* is commonly expressed in Jones $\left(\mathrm{cm}\cdot \frac{\sqrt{\mathrm{Hz}}}{W}\right)$.

Response speed is a key performance metric for PDs, typically characterized by a rise time (*τ*_r_) and a fall time (*τ*_f_) of the detector's current upon a light pulse. *τ*_r_ is the duration required for the photocurrent to increase from 10% to 90% of its maximum value upon illumination, whereas *τ*_f_ represents the time taken for the photocurrent to decay from 90% to 10% after the light is turned off. Shorter response times indicate faster and more efficient detector performance.

Detectors have versatile usage and have to stand up to the noise under low‐intensity spectra. Therefore, the SNR is used to compare the levels of the desired signal and background noise with:

(6) $$\mathrm{SNR}=P_{\mathrm{Power}}/P_{\mathrm{Noise}}$$

where *P* is the corresponding average power. LDR denotes the range of light intensities over which the PD exhibits an effective and linear photoresponse to the input signal. The LDR value can be evaluated using the following equation:

(7) $$\text{LDR}=20\log\;\frac{P_{\mathrm{Max}}}{P_{\mathrm{Min}}}$$

where *P*_Max_ and *P*_Min_ define the maximum and minimum incident light intensities in the linear photoresponse range.

Historically, photodetection has relied on material systems such as single‐crystal silicon, two‐dimensional materials, conjugated polymers, metal oxides‐sulfides, perovskite thin films, and so on [87, 88, 89, 90, 91]. Although these materials have enabled substantial progress, their practical deployment is often constrained by issues including complex and costly fabrication processes, inappropriate or zero bandgap materials, uncontrolled defects, low fabrication yields, and limited long‐term stability under operating conditions [92, 93]. These challenges have motivated extensive efforts toward material optimization through doping, interface engineering, and the exploration of alternative photoactive platforms. In this context, QDs have gained increasing attention for PD applications due to their nanoscale size‐dependent bandgap tunability, broad and controllable spectral absorption, high absorption coefficients, and capability for multiexciton generation, positioning them as highly promising building blocks for next‐generation photodetector technologies [94]. HMB QDs have already demonstrated benchmark optoelectronic performance, establishing strong competition with perovskite and emerging 2D material systems [95, 96, 97]. With the rapid expansion of wearable, implantable, and consumer‐friendly integrated electronics, the complete removal of toxic heavy metals has become critical for ensuring regulatory adherence, environmental safety, and responsible manufacturing practices. HMF chalcogenide NCs, like Ag‐, Cu‐, Bi‐, Sb‐, Zn‐, or In‐based compounds combined with S, Se, or Te, exhibit strong quantum confinement effects, enabling precise bandgap tunability across the ultraviolet (UV), visible toward SWIR, and sometimes mid‐wave infrared (MWIR) spectral regions [95, 96, 97]. Furthermore, their solution‐phase processability and compatibility with low‐temperature, large‐area fabrication make them ideal for flexible, lightweight, and cost‐effective optoelectronic platforms. In this section, we discuss the strategic integration of HMF‐chalcogenide NCs across diverse photodetection systems.

#### 4.1.2 Ligand Exchange Mediated Photoresponse Study

The performance of PDs is fundamentally determined by two primary processes: efficient optical absorption followed by charge carrier generation, separation, and extraction. Strong light absorption ensures sufficient photogeneration of electron–hole pairs, while efficient separation and collection prevent recombination losses and enable a measurable photocurrent. However, these processes alone do not fully define the device performance. Charge transport properties, including carrier mobility, diffusion length, and transit time, critically influence how rapidly and efficiently photogenerated carriers reach the electrodes. Furthermore, recombination dynamics, encompassing radiative, trap‐assisted, and Auger processes, further regulate the carrier lifetime and quantum efficiency. Colloidal QDs are generally synthesized with long‐chain organic ligands, such as oleic acid and oleylamine, which impart colloidal stability by preventing aggregation and ensuring solubility in nonpolar solvents (toluene, octane, hexane, etc.) [98]. Although these ligands are essential during synthesis and processing, their electrically insulating nature and considerable molecular length lead to increased interparticle spacing in solid‐state films, thereby forming significant barriers that suppress charge transport. As a result, ligand exchange represents a pivotal step in the fabrication of QD‐based PDs. Substituting long insulating ligands with shorter or electronically conductive species reduces interdot separation, strengthens electronic coupling, and facilitates efficient carrier transport across the QDs network toward the electrodes [99]. However, the ligand‐exchange process is considerably more complex than merely shortening interparticle spacing. Beyond improving electronic coupling through reduced interparticle spacing, ligand exchange can also reshape the surface chemistry by modifying dipole moments, oxidation states, trap‐state density, and even band alignment [100]. These changes act together to influence charge transport and recombination dynamics, ultimately determining the performance and stability of QD‐based PDs.

##### 4.1.2.1 Solid‐State Ligand Exchange

Ligand exchange in QD systems can be categorized generally into two approaches: solid‐state (post‐deposition) and solution‐phase (pre‐deposition). Solid‐state exchange is a simple, device‐compatible process, independent of colloidal stability issues. In the solid‐state method, first, stable long‐chain ligand‐capped QDs are deposited on a substrate and then treated with a solution of short‐chain ligands to induce ligand replacement. The film is subsequently rinsed with a polar solvent to remove excess of both types of ligands. Careful selection of a short‐chain ligand will ensure better charge transport, but for better light absorption, the thickness of the QD layer is optimized with either tuning QD concentration in solution or adapting a layer‐by‐layer deposition process. Different surface‐passivating short‐chain ligands dissolved in polar solvents like 1,2‐ethanedithiol (EDT), 2‐mercaptoethanol (2‐ME), 3‐mercaptopropionic acid (MPA), tetrabutyl ammonium iodide (TBAI), and 1,3‐benzenedithiol (BDT) were reported for Ag_2_Te‐ and Ag_2_Se QD‐based self‐biased photodiodes and phototransistors [101, 102, 103, 104, 105, 106, 107, 108]. For the photodiode structures, TiO_2_, SnO_2_, and ZnO‐like ETLs and spiro‐OMeTAD, MoO_3_, and poly[bis(4‐phenyl) (2,4,6‐trimethylphenyl) amine] (PTAA)‐like HTLs were used to create a built‐in potential. A schematic of a self‐biased photodetector device is shown in Figure 3a. Precise alignment of energy levels is essential to promote efficient carrier extraction and suppress the dark current, particularly in low‐bandgap materials. This alignment also regulates the internal electric field, which plays a vital role in enabling PDs to operate in the photovoltaic mode. To determine the energy levels, the valence band maximum (*E*_VB_) and Fermi level (*E*_F_) of EDT‐, ME‐, and MPA‐treated Ag_2_Te QDs, together with ZnO serving as the ETL, were investigated using ultraviolet photoelectron spectroscopy, as presented in Figure 3b [108]. Relative to ZnO, the QDs modified with EDT and ME display a slightly shallower *E*_F_, whereas the MPA‐treated QDs exhibit a ~0.14 eV deeper *E*_F_. Consequently, under equilibrium conditions in the dark, the energy bands of ZnO bend downward near the junction interface, while the bands of EDT‐ or ME‐modified QDs bend upward. In contrast, the interface of ZnO and the MPA‐modified QD forms a typical p–n junction because of the deeper *E*_F_ formation of the MPA‐passivated QD surface. This deeper *E*_F_ level facilitates improved rectification, resulting in reduced dark current density (~1.1 × 10^−4^ A/cm^2^ at −1 V) through the suppression of minority carrier diffusion, as observed in *J*_d_ versus *V* plots (Figure 3c). Furthermore, effective surface passivation by mercaptopropionic acid (MPA) ligands minimizes trap‐assisted recombination and helps to achieve the highest EQE of 0.21% at 1550 nm and 0.0072% at 2004 nm, respectively. Apart from MPA, EDT is another short ligand that is well established for solid‐state ligand exchange and was reported with Ag_2_Te QDs for self‐powered SWIR light detection, further being utilized in evolving applications like health monitoring [111]. In another instance, these EDT‐capped QDs were used with direct photopatterning by integrating the same QDs of different sizes for multispectral SWIR photodetection (device architecture shown in Figure 3d), which enables their application in high‐resolution pixelation on CMOS readout integrated circuits (ROICs) [109]. Apart from Ag‐based chalcogenides, SnTe QDs and Bi_2_S_3_ nanodots are also used in visible‐to‐MWIR photodetection after solid‐state ligand exchange with shorter ligands, such as 2‐nitrothiophene and ethanethiol [112, 113].

**FIGURE 3:**
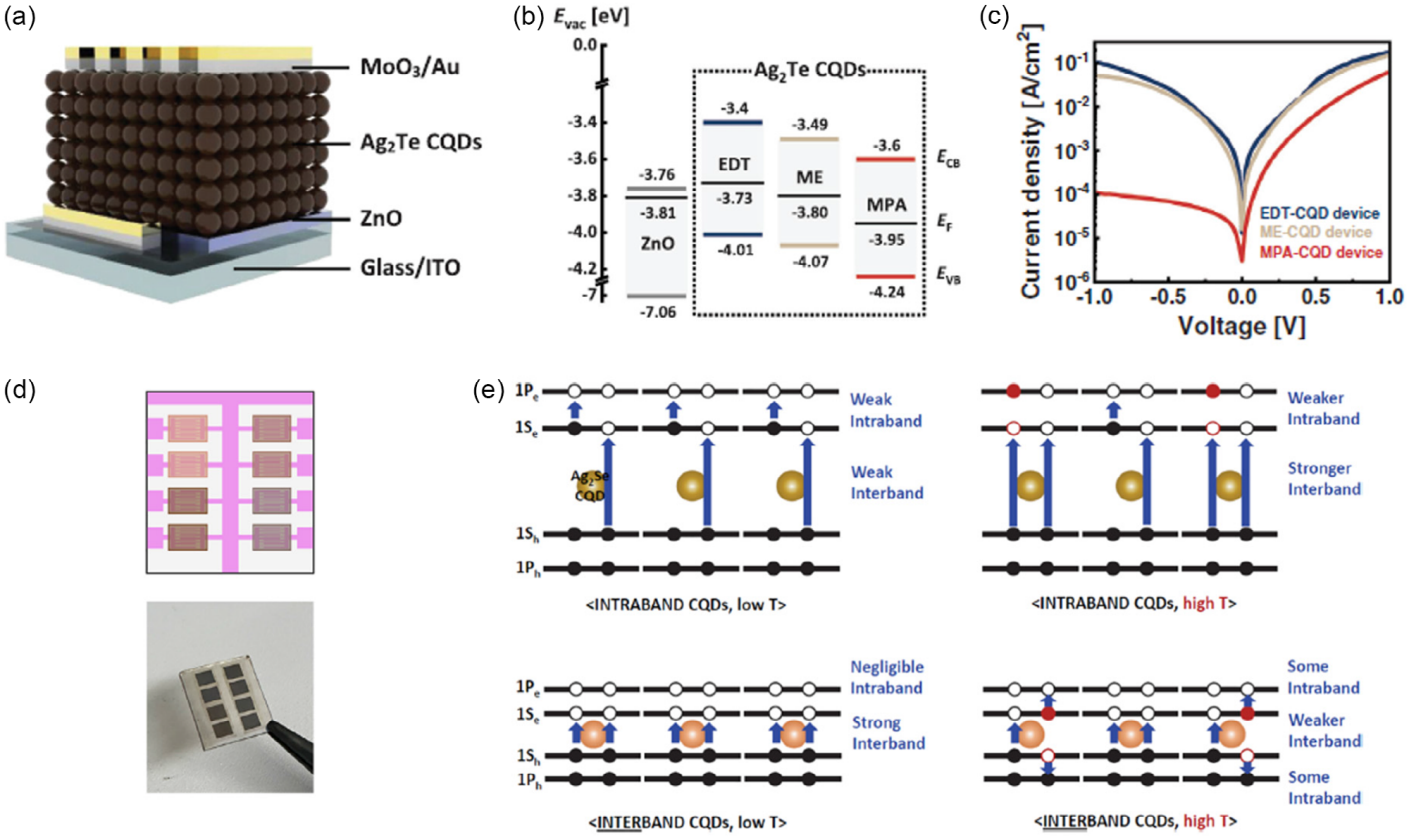
(a) Device architecture of a Ag_2_Te QD‐based self‐biased photodiode. (b) Energy‐level diagrams for ZnO (gray), EDT‐ (blue), ME‐ (beige), and MPA‐CQD (red) solids from ultraviolet photoelectron spectroscopy (UPS) measurements. *E*_vac_, *E*_F_, *E*_CB_, and *E*_VB_ indicate the vacuum level, Fermi level, conduction band edge, and valence band edge, respectively. (c) Current density–voltage (*J*–*V*) curves of EDT‐ (blue), ME‐ (beige), and MPA‐CQD (red) devices under dark conditions. Reproduced with permission from ref. [108]. Copyright 2024, Wiley‐VCH GmbH. (d) Schematic image (top) and photograph (bottom) of the multispectral Ag_2_Te QD device. Reproduced with permission from ref. [109]. Copyright 2026, Wiley‐VCH GmbH. (e) Schematics illustrating the carrier distribution and available optical transitions in intraband CQDs at low and high temperatures (left and right figures of the top panel, respectively). The CQDs with a missing electron in the 1S_e_ state represent the underoptimized doping condition. Open and closed red circles represent electron–hole pairs generated at elevated temperatures. Blue arrows illustrate available optical transitions. As a comparison, the left and right figures of the bottom panel depict carriers in typical interband CQDs at low and high temperatures, respectively. Note that the interband gap is now the MWIR‐absorbing gap. Reproduced with permission from ref. [110]. Copyright 2019, American Chemical Society.

Intraband transitions in colloidal QDs are electron movements between quantized energy levels within the same band. Due to their low energy difference, they are of great interest for mid‐infrared optoelectronics. Compared to bulk semiconductors, intraband CQDs can potentially exhibit Auger suppression, allowing high‐temperature operation of PDs due to discrete energy levels that hinder the phonon relaxation process. In Ag_2_Se QDs, excess electrons occupy the first excitonic level (1S_e_) and enable intraband transitions to the second excitonic level (1P_e_), with the transition energy tunable through size‐dependent quantum confinement. This narrow gap and electron population allow for tunable MWIR absorption. At low temperature (90 K), the QD film is underdoped, whereas at room temperature (~300 K), thermal excitation generates carriers between 1S_e_ and 1P_e_, altering the carrier distribution [110]. Consequently, intraband transitions weaken, while interband transitions strengthen with increasing temperature, reducing the intraband photocurrent. The photoresponse cutoff also redshifts at lower temperature extending beyond 7 µm at 90 K, but remains limited to ~6 µm at 300 K due to the temperature dependence of the 1S_e_–1P_e_ gap. Corresponding interband and intraband transitions are shown in Figure 3e. Furthermore, after using EDT, it can be concluded that to enhance device performance, Ag_2_Se QDs require increasing electron concentration to fully occupy the 1S_e_ level, which can be achieved using electron‐donating ligands or partial cation exchange.

##### 4.1.2.2 Solution‐Phase Ligand Exchange

The requirement of solution‐phase ligand exchange is obvious as the post‐deposition technique may result in an incomplete exchange, film shrinkage, microstructural defects, and limited mobility due to residual insulating or large‐chain ligands. Whereas this pre‐deposition ligand exchange offers more uniform surface modification and improved film homogeneity, though it can cause colloidal instability and aggregation. During this alternative ligand‐exchange process, QDs with mostly long‐chain ligands are dispersed in a nonpolar solvent and then brought into contact with short‐chain ligands dissolved in a polar solvent like DMF, acetonitrile, etc [114]. During this process, ligand substitution occurs, and the QDs, now capped with short‐chain ligands, transfer into the polar phase, enabling phase separation and purification. The exchanged QDs can subsequently be directly deposited as thin films. Solution‐phase ligand exchange in metal chalcogenide QDs is inherently challenging because of several interrelated surface and colloidal effects. These NCs are typically synthesized with strongly bound long‐chain ligands that coordinate tightly with surface metal atoms, making their displacement by shorter ligands kinetically slow and often incomplete. When these stabilizing ligands are removed, the steric protection around the QDs decreases significantly, which can lead to colloidal instability, aggregation, or precipitation of the NCs in solution. Inadequate passivation during ligand exchange can expose dangling bonds on the QD surface, leading to the formation of electronic trap states that promote nonradiative recombination and degrade device performance [115]. Additionally, metal chalcogenide surfaces are extremely sensitive to chemical perturbations, and ligand exchange can alter the surface stoichiometry, defect density, and electronic structure of the NCs, making controlled solution‐phase exchange particularly difficult. Therefore, only a few reports on stable metal‐chalcogenide‐based QD systems in polar solvents after solution‐phase ligand exchange were observed.

Halide‐ and MPA‐capped Ag_2_Te colloidal inks were prepared from oleylamine (OLA)‐capped Ag_2_Te QDs dispersed in hexane [116]. For ligand exchange, tetrabutylammonium halide (TBAX, where X = Cl, Br, or I) or MPA was mixed with the Ag_2_Te‐OLA QD solution, followed by vortexing to transfer the QDs to a polar DMF/acetonitrile (3:2) mixed solvent. Both halide and MPA‐Ag_2_Te QD inks were spin‐coated one after another to build a multilayer absorbing layer of a self‐powered photodetector. Iodide (I^−^) ligands can provide effective surface passivation of QDs compared to other halide (Cl^−^ or Br^−^)‐based ligands because of their higher atomic‐scale size, enabling close and uniform coverage of surface sites, which should also enhance air stability through improved steric shielding of the nanocrystal surface. Kim et al. [116] reported that the iodide ligands were necessary to preserve the colloidal stability of the QDs, while uniform iodide‐only passivation was not sufficient to produce stable Ag_2_Te QD inks as it led to particle aggregation. The presence of Cl^−^ in the ligand‐exchange solution efficiently prevents aggregation of the ink. Therefore, from a comparative study on 3 halide ions mixed (Cl/Br/I‐Ag_2_Te ink) and two halide ion mixture solution (Cl/I‐Ag_2_Te ink), it was observed that the Cl/I^−^Ag_2_Te ink showed a low trap density (5.8 × 10^15^ cm^−3^) with higher electron mobility (8.9 × 10^−2^ cm^2^ V^−1^ S^−1^), as shown in Figure 4a. Capping with MPA ligands imparts a comparatively p‐type character to the QDs relative to their halide‐passivated counterparts. Consequently, the photodetector architecture consisting of ZnO (ETL)/halide‐Ag_2_Te CQD ink/MPA‐Ag_2_Te CQD ink/MoO_*x*_ establishes an n–i–p device configuration (band alignment in the device shown in Figure 4b). This particular device exhibited an EQE value of ~16% (at 0.3 V bias) with a responsivity of *R* *_λ_* ~ 0.23 A/W at peak absorption in the deep SWIR region (peak value ~1700 nm).

**FIGURE 4:**
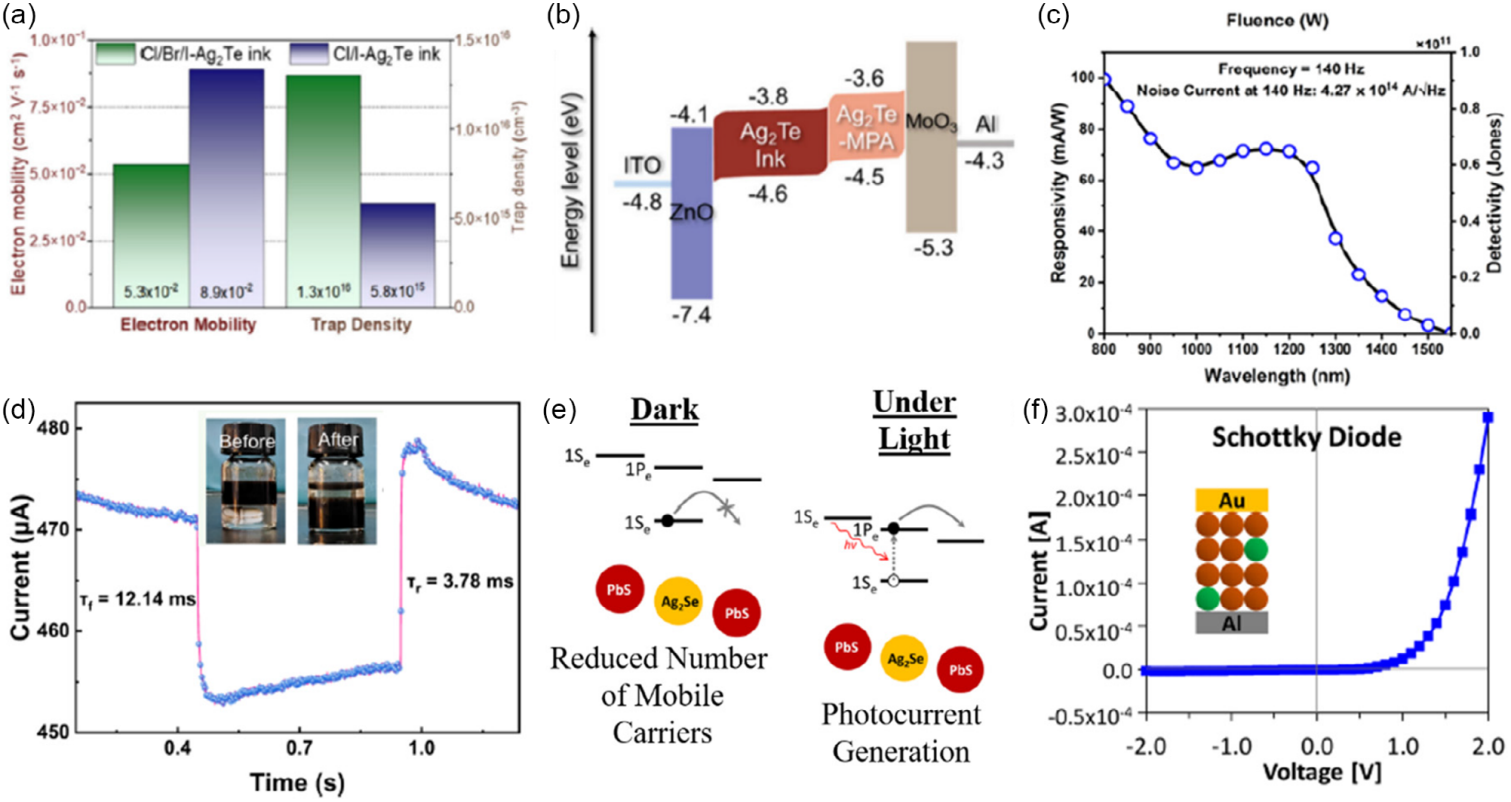
(a) Electron mobility and trap density from the space‐charge‐limited current measurements of the devices with various ratios of halide ligands. (b) Energy level alignment of the Ag_2_Te photodiode device. Reproduced with permission from ref. [116]. Copyright 2024, American Chemical Society. (c) Responsivity and detectivity versus wavelength for a diode measured at 140 Hz. Reproduced with permission from ref. [117]. Copyright 2025, American Chemical Society. (d) Transient photoresponse of graphene‐Ag_2_Se QDs photodetector. In the inset, photos shows solution‐phase ligand exchange of Ag_2_Se QDs. Reproduced with permission from ref. [118]. Copyright 2025, American Chemical Society. (e) Schematic depiction of the intraband charge transfer mechanism in a binary Ag_2_Se/PbS mixture film, both in the dark and under IR illumination. (f) Confirmation of Schottky junction formation observed in *J–V* plot for the Al/EDT‐capped binary QDs/Au detector. In the inset schematic, the device structure is shown. Reproduced with permission from ref. [119]. Copyright 2023, Royal Society of Chemistry.

In another work by Paul et al. [117], Ag_2_Se QDs were modified with a mixture of AgI and ME to utilize in both photodiode and photoconductive devices. For the photodiode structure in self‐biased mode (0 V bias condition), EQE reached to ~7.39%, D* ~ 6.5 × 10^10^ Jones, and *R* *_λ_* ~ 70 mA/W (Figure 4c), while in the photoconductive device under 0.3 V bias, the *R* *_λ_* value increased to ~142 mA/W at the wavelength of 1200 nm. After adding ME in the liquid ligand‐exchange procedure, the photovoltaic device characteristics were improved, after comparing with the Ag_2_Se QDs‐based photodetector reported by Li et al. [120] In another report, sodium tetrathioarsenate (Na_3_AsS_4_·8H_2_O) was used as a ligand to passivate the Ag_2_Se QDs (phase transfer is depicted in the inset of Figure 4d) and employed with a graphene sheet for photodetection in a field‐effect transistor architecture [118]. Such Graphene/Ag_2_Se QD NIR PDs exhibited a broad spectral detection from 808 to1550 nm with a maximum *R* *_λ_* value of ~2.878 × 10^4^A W^−1^, D* of ~2.35 × 10^13^ Jones along with a photoresponse speed (transient photoresponse is shown in Figure 4d) in the microsecond range at 100 mV bias with a photocurrent of 66.60 μA under 808 nm illumination (3.5 mW cm^−2^), where ligand exchange significantly enhanced the Ag_2_Se QD performance, highlighting their potential for compact, low‐power, and cost‐effective NIR PDs.

#### 4.1.3 Interface Engineering Mediated Photoresponse Study

Interface engineering is essential for achieving high‐performance PDs because the junctions between different materials strongly influence carrier dynamics. Poor interfaces often introduce defects that result in a degraded device efficiency. By carefully tailoring the interface, proper band alignment and efficient charge separation can be achieved [121]. For example, interfaces between active layers and electrodes can also strongly influence carrier transport. By optimizing band alignment and introducing passivation or interfacial layers, charge separation and carrier extraction can be significantly improved [122]. Interface modulation can primarily be achieved through surface passivation, heterojunction engineering, and composite formation.

##### 4.1.3.1 Ligand‐Less Surface Passivation

Surface passivation is crucial for improving the performance of QD‐based PDs. The high surface‐to‐volume ratio of QDs creates many surface defects and dangling bonds that act as trap states, causing carrier recombination. Effective surface passivation will reduce the number of traps, enhance charge transport, and lower the dark current, thereby improving the overall photodetector performance. Annealing is a vital procedure to form crystalline QD‐based thin films. Apart from residual solvent and excess ligand removal, this also enables closer QD packing and stronger interparticle coupling. With annealing, defects and trap states can be modified, which can help to enhance charge transport and ultimately improve photodetector performance. Lee et al. demonstrated this approach for spin‐coated Ag_2_Se QDs, followed by annealing at 100 and 150 °C, to fabricate a flexible NIR PD [123]. Films annealed at 150 °C showed highly crystalline Ag_2_Se QDs with higher conductivity, yielding a high D* value of ~7.14 × 10^9^ Jones, with stable and faster photoresponse than those annealed at 100 °C. The formation of metallic Ag may be attributed to the Ag^+^ ions, which enhance electrical conductivity and passivate trap states inside the Ag_2_Se film. These effects can facilitate faster charge extraction and suppress carrier recombination, ultimately contributing to the improved performance of such flexible PDs.

##### 4.1.3.2 Formation of Schottky Junction

Schottky junction PDs are advantageous because the metal‐semiconductor barrier enables efficient photocarrier separation and low dark current while offering simpler fabrication than p–n junction devices and better rectification than Ohmic‐contact photoconductors [124]. Mahfuz et al. [119] reported MWIR Schottky photodiodes based on a binary QDs film of intraband Ag_2_Se and PbS QDs. Binary QDs selectively block ground‐state electron and hole transport while allowing photoexcited electrons to flow freely, resulting in enhanced photocurrent. An intraband charge‐transfer mechanism in binary QDs both in the dark and under illumination is shown in Figure 4e. After finalizing the optimized ligand, these binary QDs were utilized in photodetection by sandwiching between Au contacts but suffered from high dark current. The primary source of dark current in a photoconductive device is the majority carrier drift current; therefore, the Au bottom contact (work function = 5.2 eV) was replaced with low work function Al (work function = 4.1 eV), which helped to form a Schottky contact (photoconductive device structure: Al/binary QDs/Au, schematic shown in Figure 4f). The Schottky junction formation resulted in a reduction of noise current by an order of magnitude, strong rectification with an on/off ratio of ~210 (at ±2 V), corresponding *I*–*V* curve is shown in Figure 4f. Among these tested ligands, EDT based device showed better infrared photoresponse (*R*_3µm_ ~ 0.17 A/W and *R*_4.5µm_ ~ 0.11 A/W) with D* in the order of ~10^7^ Jones at room temperature. In another lateral photodetector device, using similar Ag contact on SnSe QDs, non‐Ohmic photoconductivity in the visible to NIR spectral region was reported with a responsivity R_780 nm_ of ~401 μA/W and a corresponding D* of ~10^8^ Jones in ambient conditions [125]. Moreover, faster photoswitching in vacuum conditions highlights the influence of environmental factors, such as oxygen, on Se‐deficient SnSe QDs‐based PDs.

##### 4.1.3.3 Core–Shell Formation

Another efficient way to passivate the surface defects is the formation of a core–shell structure, typically assisting in the suppression of nonradiative recombination and prolongation of carrier lifetime, thereby influencing photocurrent, EQE, and other parameters in optoelectronic devices. For example, SnTe, a narrow‐bandgap semiconductor (0.18 eV at 300 K) with high mobility (~230 cm^2^ V^−1^ s^−1^) and carrier concentration (~10^21^ cm^−3^), is promising for IR applications but suffers from rapid oxidation. To overcome this issue, Martin et al. [126] employed an air‐stable ZnTe shell with an oxygen‐free 1‐dodecanethiol (DDT) ligand instead of air‐sensitive tin silylamide and oleic acid, enabling the synthesis of SnTe QDs stable for up to 1 month without noticeable oxidation.

Optically active type‐I heterostructures were formed in the case of MgS shell‐passivated ZnS core QDs (ZnS@MgS) [127]. This core–shell structure generated electron–hole pairs primarily in the ZnS core under UV illumination, followed by charge transport to the electrodes through the builtin ‐electric field, shown in Figure 5a. The MgS shell, owing to its wide bandgap, acted as an effective filter for non‐UV radiation, reducing interference from longer‐wavelength photons and thereby improving the device sensitivity. This ZnS@MgS core–shell‐based photodetector demonstrated significantly higher conductivity with a 60 000‐fold improvement in photocurrent compared to the only ZnS QDs‐based device, observed in phototransient curves measured at 1 and 20 V bias conditions (Figure 5b). Superior charge transport properties at the interface of ZnS and MgS layers, followed by effective suppression of surface traps by the MgS shell, ensuring enhanced carrier mobility. Another type‐I Ag_2_Se@Ag_2_S core–shell heterostructure was reported in self‐powered photoelectrochemical‐type PDs [131]. After the introduction of the Ag_2_S shell, the performance became faster (from ~850 to ~700 ms), along with a ~47‐fold improved D* (~4.5 × 10^11^ Jones) and an excellent S value of ~1450 under 940 nm NIR light. In another study, ZnO nanoneedle arrays loaded with Ag_2_S QDs formed a type‐II heterojunction (charge‐transfer mechanism shown in Figure 5c) to build an efficient self‐powered broadband (400–1100 nm) photodetector with an *R*_440 nm_ value of ~0.13 A/W [128]. The benefit of the core–shell structure was extended by Lee et al. to the deep SWIR region by utilizing an Ag_2_Te@Ag_2_S core–shell structure in a self‐powered photodiode device with a remarkable EQE of ~8.4%, *R* *_λ_* of ~100 mA/W, and D* up to ~10^11^ Jones at 1550 nm [132].

**FIGURE 5:**
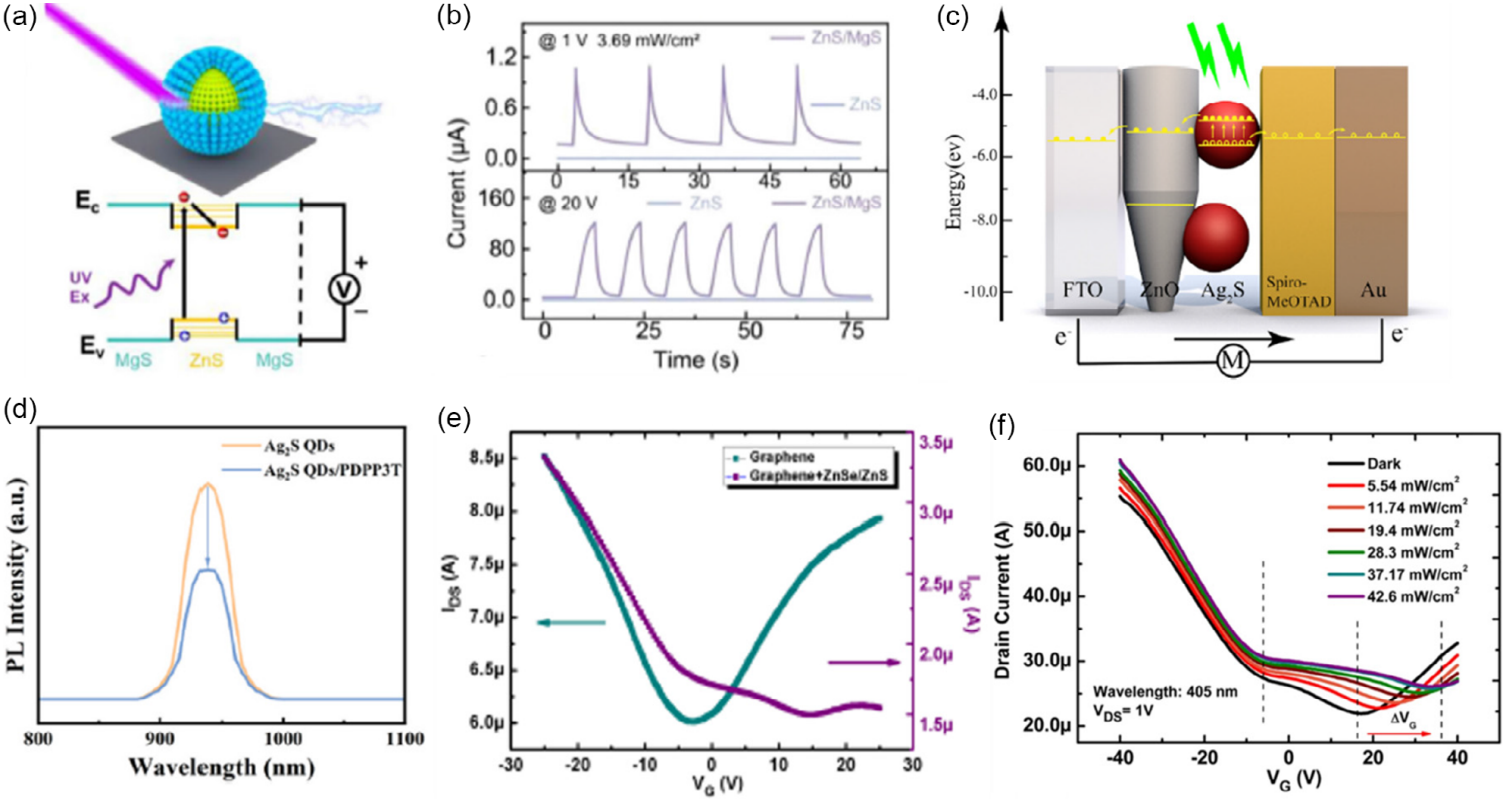
(a) Schematic diagram of the working mechanism of the ZnS@MgS core–shell nanostructure device. (b) Comparison of the *I–t* curves between ZnS@MgS core–shell nanostructures and ZnS QD PDs at bias voltages of 1 and 20 V under an illumination intensity of 3.69 mW/cm^2^. Reproduced with permission from ref. [127]. Copyright 2025, Wiley‐VCH GmbH. (c) Energy band structure showed formation of a type‐II heterojunction between ZnO nanoneedle array and Ag_2_S QDs, used in self‐biased photodetection. Reproduced with permission from ref. [128]. Copyright 2018, Elsevier. (d) PL spectra of Ag_2_S QDs and the Ag_2_S QDs/PDPP3T heterojunction. Reproduced with permission from ref. [129]. Copyright 2026, Elsevier. (e) The transport characteristics of graphene‐based FET before (blue line) and after (purple line) the ZnSe@ZnS core–shell QDs spin‐coated on the graphene surface. (f) Transfer characteristics of graphene‐based phototransistors decorated with ZnSe/ZnS quantum dots under different light irradiation with the wavelength of 405 nm. Reproduced with permission from ref. [130]. Copyright 2018, Springer Nature.

##### 4.1.3.4 Hybrid Composite or Layer‐by‐Layer Heterojunction Formation

Constructing heterostructures is a widely adopted strategy for improving photodetector performance, from efficient charge transfer to broadening the spectral detection range. Two‐dimensional (2D) transition metal dichalcogenides (TMDs) are attractive due to their high in‐plane carrier mobility, thickness‐dependent bandgap, strong light absorption, and remarkable stability. Therefore, the advantage of the spectral tunability of zero‐dimensional (0D) QDs can be integrated onto large‐area crystalline layers of 2D materials, which not only broadens the spectral limit, but the interaction between 0D and 2D materials can also improve the dispersion and stability of QDs [133]. In addition, rapid charge transfer through the 2D nanostructures suppresses charge recombination, leading to a significantly improved photodetection performance. UV light‐sensitive SnS_2_ QDs were deposited on 2D MoS_2_ sheets on a silicon substrate for broadband (UV‐NIR) photodetection. The corresponding *R* *_λ_* values under UV, visible, and NIR light were 278, 435, and 189 A/W, respectively, at 1 V applied bias condition [134]. Similarly, 2D/0D heterojunctions for improved and broad spectral detection were also reported with Ag_2_S, ZnS, and Bi_2_S_3_ QDs decorated 2D SnS_2_ nanosheet, organic poly(diketopyrrolopyrrolothiophene) (PDPP3T), bimetallic CuMoO_4,_ and WO_3_ nanoplates [129, 135, 136, 137]. The quenching observed in the PL spectra of microwave heating‐grown Ag_2_S QDs after spray coating with a 2D organic layer of PDPP3T confirmed efficient photoexcited carrier separation at the Ag_2_S QDs/PDPP3T interface, which suppresses corresponding radiative recombination transport, which helps in a one‐order enhancement in *R* *_λ_* and D* values measured at 700 nm wavelength (shown in Figure 5d) [129].

Graphene exhibits an exceptionally high electron mobility (~1.5 × 10^4^ cm^2^ V^−1^ s^−1^) and has proven to be an efficient electron acceptor in hybrid systems in combination with semiconductor nanoparticles such as QDs. Cu_2_S, ZnS, and SnSe_2_ QDs on graphene or reduced graphene oxide‐based substrates were developed for UV and blue light detection [138, 139, 140]. Moreover, a hybrid phototransistor consisting of a CVD‐grown monolayer of graphene and ZnSe@ZnS core–shell QDs demonstrated strong UV photodetection with an *R*_405 nm_ above 10^3^ A W^−1^ at 405 nm, where photoexcited carriers transfer from the QDs to the graphene, enabling a gate‐tunable photoresponse with a maximum at ~15 V [130]. The transfer characteristics, drain current (*I*_DS_) versus gate voltage (*V*_G_), of the field‐effect transistors in the dark showed ambipolar behavior (typical V‐shaped curve) only for the plain graphene sheet, whereas after deposition of the ZnSe@ZnS QDs onto the graphene, a change in that transfer curve was observed, indicating only p‐type conduction dominance (shown in Figure 5e). To get more insight into the charge‐transfer mechanism, 405 nm illumination with different intensities was tested, as shown in Figure 5f. Conductance of the graphene channel enhances with an increase in light irradiation when hole carriers are dominant, i.e., *V*_G_ < *V*_Dirac_ (Dirac point), but the completely opposite phenomena is observed when electron carriers are dominant (*V*_G_ > *V*_Dirac_). Therefore, it can be concluded that under illumination, graphene becomes strongly p‐doped due to hole accumulation. The transfer of holes to graphene exceeds that of electrons, leaving excess electrons in the QDs and inducing additional holes in graphene. Consequently, a more positive gate voltage is required to reach the charge neutrality point, while the increased hole concentration leads to a higher channel current.

p–n heterojunctions are widely used in QDs‐based PDs because their built‐in electric field at the interface efficiently separates photogenerated electron–hole pairs and efficiently reduces recombination. This enhances charge transport and reduces dark and noise current, along with an improved responsivity. Such devices were realized using p‐type CsPbBr_3_ QDs as the primary absorption layer and n‐type Ag_2_S QDs as the secondary absorption layer [141]. Such a p–n heterojunction facilitated efficient charge transfer and suppressed carrier recombination, leading to a broad spectral response from 350–1040 nm (vis‐NIR). The Ag_2_S/CsPbBr_3_ photodetector showed an *R* *_λ_* value of ~11.3 A/W, a D* value of ~3.55 × 10^10^ Jones, and a low NEP of ~4.28 × 10^−12^ W under 405 nm illumination, along with a response time of 31–34 ms under 532 nm irradiation. Embedding nanoparticles or QDs within a polymer matrix offers additional functionalities such as improved solubility, reactivity, assembly, miscibility, and processability while also enhancing the long‐term material stability and performance. ZnSe QDs embedded in a polymer matrix were successfully used in UV detection, where polymer capping helped to reduce the number of surface traps [142]. Whereas, in another study, polymer‐capped Ag_2_S QDs enabled visible‐NIR photodetection for which conductive polymers helped in charge transportation [143, 144].

#### 4.1.4 Doping and Plasmon Mediated Photoresponse Study

Doping plays a significant role in the precise control of the electronic structure, optical properties, and the charge transport properties in QD‐based devices [145]. The incorporation of suitable dopant atoms can modify the carrier concentration, tune the *E*_F_, and optimize the band alignment between QDs and adjacent transport layers or electrodes in a PD [146]. Moreover, doping can reduce trap states and suppress carrier recombination and also enable broader spectral absorption. Consequently, doping serves as an effective strategy to enhance the key parameters of optoelectronic devices.

For example, the intrinsic defect structure of stable, visible light‐sensitive β‐In_2_S_3_ QDs exhibits interesting electronic, optical, and optoelectronic properties. Doping of such QD‐based thin films influenced the distribution of electron density by modulating the concentration of defects [147]. Pure In_2_S_3_ behaves as an n‐type semiconductor due to the presence of sulfur vacancies, with photoconduction mainly dominated by electron transport [148]. The S value increased from 78 for pristine QDs to 217 and 185 for Mn‐ and Cu‐doped QDs at 10 V bias, respectively. An increase in sulfur defects and the introduction of more localized states upon d‐element doping are the main reasons for the high gain and lower photo‐carrier lifetime in the Mn‐ and Cu‐doped samples. Furthermore, this enhancement is also attributed to an improved carrier mobility under illumination, oxygen desorption from the surface, and defect states that prolong electron lifetime, allowing multiple carrier circulation and thus a higher photoconductive gain.

Hot injection‐induced Bi‐doping in Ag_2_Te QDs (~0.88 eV) showed a downward (toward the VB) shift of the Fermi level due to the introduction of Bi^3+^ electron‐acceptor states near the VB, which improved the p‐type conductivity of QDs, as shown in Figure 6a [149]. After doping, Bi‐doped Ag_2_Te QD‐based self‐powered photodiode (device schematic shown in Figure 6b) exhibited an improved D* value of 7.67 × 10^10^ Jones in comparison to 1.66 × 10^9^ Jones for the undoped QDs‐based detector, at 1460 nm during measurement at room temperature (shown in Figure 6c). Despite having excellent chemical stability and inherent ductility, Ag_2_S QDs exhibit a lower optoelectronic performance due to the low formation energy of silver vacancies that generate deep trap states, along with poor carrier mobility caused by strong scattering at numerous domain boundaries in QD thin films. Bi doping in Ag_2_S was reported by Cao et al., suppressing Ag^+^ migration and enhancing the power conversion efficiency in all‐solid‐state devices [152]. In this method, Ag^+^ migration was suppressed with the connection of small‐molecule ligands to cap the QD surface through the I─Ag bond by adding BiI_3_ during the formation of Bi‐doped Ag_2_S QDs. Therefore, without further ligand exchange, the Bi‐doped Ag_2_S QDs were directly used for film deposition and exhibited a sixfold enhancement in *R* *_λ_* value. Lei et al. [153] introduced an additional surface treatment using EDT, MPA, and thioglycolic acid (TGA) in a solid ligand exchange on Bi‐doped Ag_2_S QD films to improve the thin film quality. A thiol‐based small‐molecule treatment effectively modulated the electronic structure of the QD films since sulfhydryl‐containing molecules strongly coordinate with metal cations (Ag^+^ and Bi^3+^), passivating uncoordinated surface sites and eliminating dangling bonds. An EDT‐treated Bi‐doped Ag_2_S QD‐based detector showed a ~1.8 times enhancement in *R* *_λ_* from the device without passivation under 650 nm visible light.

**FIGURE 6:**
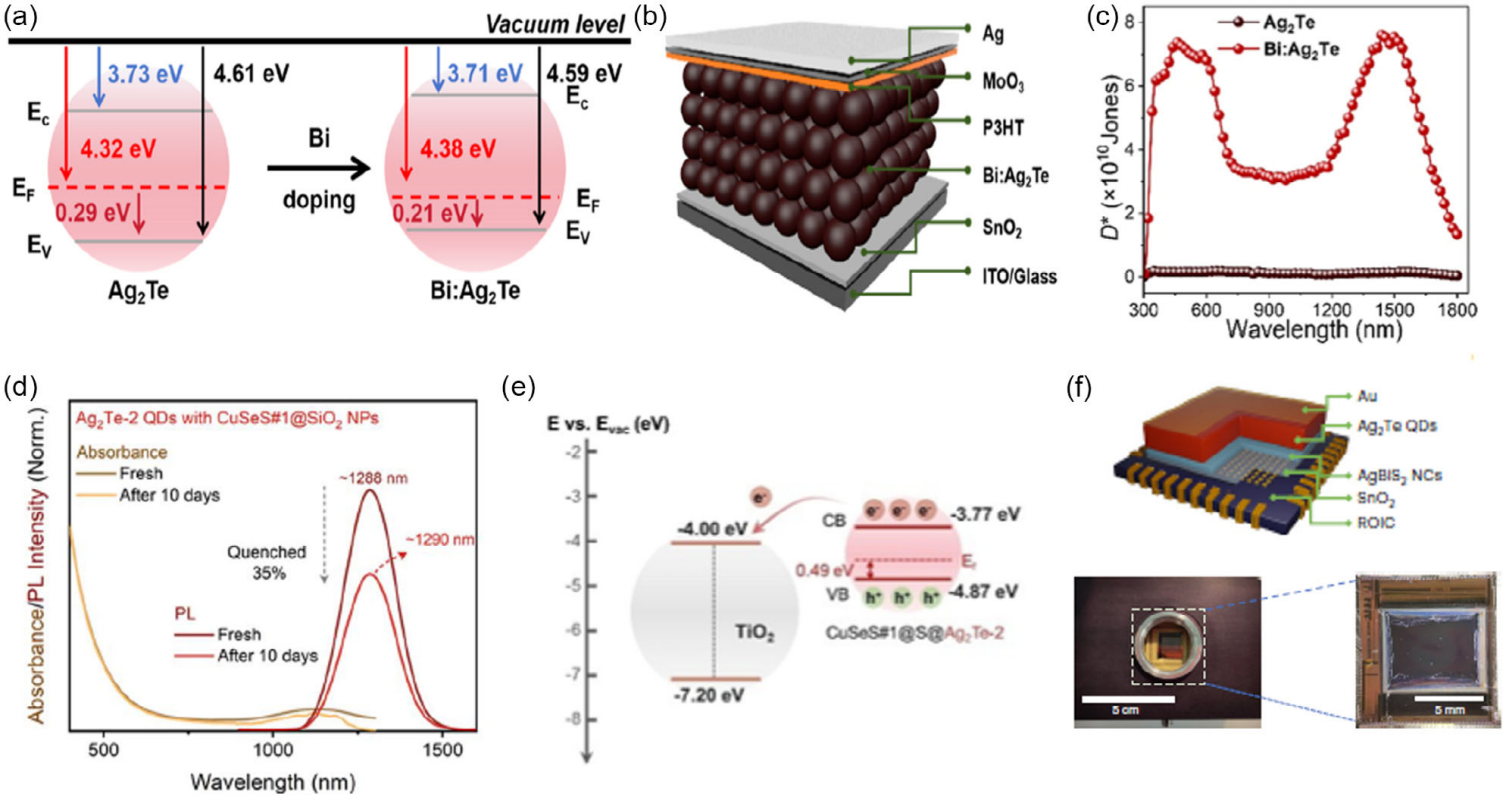
(a) Energy level positions of Ag_2_Te and Bi‐doped Ag_2_Te QDs were calculated from UPS measurements. (b) Schematic of a self‐powered photodiode device made of Bi‐doped Ag_2_Te QDs. (c) Corresponding broadband‐specific detection‐rate spectra (D*) of Ag_2_Te and Bi‐doped Ag_2_Te QDs photodiodes at zero bias. Reproduced with permission from ref. [149]. Copyright 2025, American Chemical Society. (d) PL decay curves (*λ*_ex_ = 405 nm) of the original Ag_2_Te QDs and CuSeS#1@S@Ag_2_Te‐2 QDs. (e) Energy band alignment diagram of the fabricated CuSeS#1@S@Ag_2_Te‐2‐based PDs. Reproduced with permission from ref. [150]. Copyright 2025, Royal Society of Chemistry. (f) Schematic of ROIC‐integrated Ag_2_Te QD SWIR imager with photograph of the imager and zoomed‐in view of the ROIC die (bottom panel). Reproduced with permission from ref. [151]. Copyright 2024, Springer Nature.

Localized surface plasmon resonance (LSPR) is widely employed to improve the performance of PDs. Especially for HgTe QD‐based photodetector systems, benefits of plasmonic structures on the device performance were reported [154]. LSPR originates from the resonant collective oscillation of conduction electrons in metallic nanostructures when they interact with incident light at particular frequencies. Incorporating such plasmonic nanostructures into QD films generates intense localized electromagnetic fields near the metal surface, which enhance optical absorption within the QD‐based thin films. This enhanced field facilitates greater exciton generation and more efficient separation of photogenerated carriers. CuSeS#1@SiO_2_ nanoparticles were used to decorate Ag_2_Te QDs using solution‐phase mixing and drop‐casting on mesoporous TiO_2_ in a photoconductivity study [150]. After the nanoparticles were integrated with the Ag_2_Te QDs, the modified QDs became more stable, as evidenced by a smaller quenching of the PL (drop only ~35% after 10 days) compared to 62% in 10 days for the nondecorated QDs. In addition, LSPR‐induced carrier lifetime enhancement was observed (as shown in Figure 6d). The lifetime values increased from 13.6 ns (faster lifetime component, *τ*_1_) and 46 ns (slower lifetime component, *τ*_2_) to corresponding values of 17.8 ns (*τ*_1_) and 63.2 ns (*τ*_2_), respectively. The corresponding modified *E*_CB_, *E*_VB,_ and *E*_F_ energy levels allowed to form a type‐II band alignment with TiO_2_, which promotes the separation and transfer of photoinduced charge carriers between QDs and TiO_2_ (shown in Figure 6e), to achieve a ~4.18‐fold enhancement in *R*_980nm_ (14.1 A/W) and ~2.89‐fold enhancement in *R*_1064nm_ (19.8 A/W) under a 1 V bias condition.

#### 4.1.5 Photoresponse Study for Ternary Metal Chalcogenide QDs

Unlike binary material systems, ternary metal chalcogenide QDs are often more complex to synthesize since the reactivity of the different components must be balanced. Consequently, ternary QDs exhibit stronger composition‐dependent optoelectronic properties and device performance.

##### 4.1.5.1 Ligand Exchange in Ternary QDs

A solid‐state ligand‐exchange process using thiol (EDT)‐ and halide (TBAI)‐based short ligands in Ag_2_Te_*X*_S_1−*X*_ and CuInSe_2_ QDs were used for visible to NIR photodetection [155, 156]. Eco‐friendly AgBiS_2_ QDs were also used in visible light detection in a layer‐by‐layer structured device after solid‐state ligand exchange with tetramethylammonium iodide (TMAI). After adding a polyelectrolyte as a cathode buffer layer on the surface of the ETL (ZnO), the *R* *_λ_* value was enhanced by 17% in the visible spectrum and also an improvement the dynamic characteristics under 940 nm NIR light [157] was observed. Moreover, a solution‐phase ligand exchange with MPA was reported by Guo et al. for CuInSe_2_ QDs and applied in UV to SWIR (300–1100 nm) broadband photodetection [158]. The ligand exchange (from oleylamine to MPA) improved the electron transport, resulting in an *R*_1000 nm_ of ~0.5 A/W and a corresponding D* value of ~7.5 × 10^10^ Jones, at zero‐bias conditions.

##### 4.1.5.2 Interface Engineering in Ternary QDs

Interfacial recombination between ETL SnO_2_ and the absorbing layer of Ag_2_Te QDs typically causes poor device performance. Therefore, a buffer layer of AgBiS_2_ QDs was used to act as an intermediate electron acceptor layer, providing lower carrier density and a better band alignment compared to SnO_2_ in a self‐powered photodetector architecture [151]. With this modified band alignment, Ag_2_Te QDs exhibited an improved performance in the SWIR region, with a wide LDR of over 118 dB and a high D* value of over 3 × 10^12^ Jones, which were integrated into silicon ROICs to demonstrate a nontoxic, solution‐processed integrated SWIR imager operating at room temperature. The Au‐top‐electrode‐based stack was used for an image sensor by integrating it on an ROIC to form a focal plane array. A schematic of the ROIC‐integrated Ag_2_Te QD SWIR imager, along with a picture of the imager and a zoomed‐in view of the ROIC die, is shown in Figure 6f. The silicon ROIC plays a critical role by collecting the electrical charges generated by the light‐absorbing Ag_2_Te layer and converting them into readable digital signals for image processing.

In other works, ZnS and ZnSe were reported as the shell of a core–shell structure with CuInSnSe and CuInS_2_ QDs to be utilized in photodetector applications [159, 160, 161]. An organic–inorganic heterojunction architecture was developed to enhance the performance of CuInS_2_@ZnS core–shell QD PDs by improving exciton dissociation and charge transport. The device consists of a CuInS_2_@ZnS core–shell structure sandwiched between two organic blend layers composed of the HTL, poly(4‐butyl‐N,N‐diphenylaniline) (Poly‐TPD), and the phenyl‐C61‐butyric acid methyl ester (PCBM) as the ETL. An energy‐favorable band alignment between the QDs and the organic layers enabled efficient exciton separation and rapid charge transport, with holes transferred to Poly‐TPD and electrons to PCBM. As a result, the PDs exhibited an *R* *_λ_* value of ~0.86 A/W with a D* value of ~3.76 × 10^11^ Jones and an EQE of ~262% at −40 V gate bias, along with a fast response time below 5 ms.

##### 4.1.5.3 Beyond Ligand Exchange and Interfacial Studies in Ternary QDs

To mitigate the limited carrier transport and trap‐related recombination issues in these QDs, heat treatment, halide and thiol‐like ligand passivation, and mixed‐size QDs usage were reported to improve photodetection [162, 163]. Heat treatment helps reduce the amount of surface functional groups like amines and thiols originating from ligands and also the defects present on the film surface. Heat‐treated (200 °C) AgBiS_2_ and Ag_2_S QDs‐based PDs were prepared with comb‐shaped electrodes to measure NIR light. Heat treatment improved the photocurrent response of AgBiS_2_ and Ag_2_S. Heat treatment above the phase‐transition temperature (AgBiS_2_: 255 °C and Ag_2_S: 178 °C) caused phase segregation and resulted in deviations from the stoichiometry. Mixed‐sized (average particle sizes of 4.7 ± 1.0 and 7.1 ± 1.6 nm) AgBiS_2_ QDs were designed to facilitate the energy band alignment and acted as the main absorbing layer for UV to NIR range (350–1070 nm) spectral detection with a D* value above 3 × 10^11^ Jones [164]. Moreover, Yang et al. [165] synthesized nontoxic, air‐stable bismuth ternary chalcogenides with the formula of ABiX_2_ (A = Na, K, or Cs, and X = S or Se) nanocrystals (NCs) having narrow size distributions with strong absorption in the spectral range from visible to NIR region. Among them, the KBiS_2_ nanocrystal‐based device showed a better photoresponse with an excellent D* value of approximately 5.1 × 10^15^ Jones under 525 nm visible laser illumination. For a better understanding of the relation between photodetection mechanisms and materials chemistry, recent material studies and changes in their corresponding performance parameters with different ligand exchange procedures are summarized in Table 1.

**TABLE 1:** PDs device performances based on HMF chalcogenide NCs PDs (solid state ligand exchange represented as SSLE and solution phase ligand exchange represented as SPLE).

Device structure	Bias, V	Wavelength, nm	Ligand exchange	EQE, %	***R*** *_λ_* **,** **mA/W**	D* (×10 ^**9**^ **) (Jones)**	References
FTO/TiO_2_/Ag_2_Se/Spiro‐OMeTAD/Ag	0.5	1100	SSLE (1,3‐benzenedithiol)	0.6	4.17	—	[101]
ITO/ZnO/Ag_2_Te/MoO_3_/Ag	1.5	1550	SSLE (EDT/MPA/TBAI)	~4	27.6	1.3	[102]
ITO/ZnO/Ag_2_Te/PTAA/MoO_3_/Ag	−0.3	1550	SSLE (EDT)	30	380	0.14	[103]
Si/Ag_2_Te/Al	1.5	1050	SSLE (EDT)	—	150 000	4800	[104]
	0				1030	5600	
ITO/ZnO/Ag_2_Te/MoO_3_/Ag	0	1550	SSLE (EDT)	—	2	—	[105]
FTO/TiO_2_/Ag_2_Te/Ag	—	1400	SSLE (1,3‐benzenedithiol)	0.14	1.5	—	[106]
ITO/SnO_2_/Ag_2_Te/Au	−1	1450	SSLE (EDT + AgNO_3_)	~30	~250	~110	[107]
	−0.5			~27			
	0			>20			
ITO/ZnO/Ag_2_Te/MoO_3_/Au	−0.3	1550	SSLE (MPA/ME/EDT)	0.21	~2	0.01	[108]
		2004		0.0072	—	0.0061	
ITO/ZnO/Ag_2_Te/MoO_3_/Au	0	1000	SSLE (EDT)	0.9	7.22	89	[111]
		1400		0.54	—	28.1	
ITO/Mixed sized Ag_2_Te QDs	0.2	1150	SSLE (EDT)	20.3	188.4	4.1, 2, 0.99 and 2.5	[109]
		1350		15.9	173.5		
		1550		12.4	155.6		
		1800		12.8	185.8		
Si/SiO_2_/Au/SnTe	10	637	SSLE (1 2‐nitrothiophene)	—	48	18	[112]
		940			3.7	1.3	
		1310			0.11	0.023	
		1550			0.03	0.012	
		2000			0.03	0.0014	
Si/SiO_2_/Cr/Au/Ag_2_Se	4	4500 nm (90 K)	SSLE (EDT)	—	0.35	—	[110]
ITO/ZnO/Ag_2_Te–Ag_2_S Core–Shell/MoO_3_/Au	−0.5	1550	SSLE (EDT)	8.4	100	110	[132]
ITO/SnO_2_/Bi‐doped Ag_2_Te/P3HT/MoO_3_/Ag	0	1460	SSLE (MPA)	11.2	130	76.7	[149]
	0.3			30	330	—	
FTO/ZnO/Bi‐doped Ag_2_S/PTB7/MoO_3_/Ag	0.3	635	SSLE (TGA/MPA/EDT)	—	195.31	—	[153]
ITO/SnO_2_/AgBiS_2_/Ag_2_Te/Au	0	1400	SSLE (EDT)	~20	~220	—	[151]
	−0.1			~30	—		
Ag_2_Te_*x*_S_1−*x*_/Ti/Au	0.5	1350 nm	SSLE (EDT)	—	0.48	9.1	[155]
ITO (ITO‐PEN Substrate: Flexible)/PEDOT:PSS‐MoS_2_/CuInSe/ZnO/Ag	0	470 nm	SSLE (TBAI)	23.2	87.9	2100	[156]
ITO/ZnO/PFN‐Py/AgBiS_2_/PTB7‐Th/MoO_3_/Ag	−0.5	680	SSLE (TMAI)	—	550	1400	[157]
ITO/ZnO/Ag_2_Te/MoO_3_/Al	0.3	1700	SPLE (MPA)	16	230	90	[116]
FTO/ZnO/Ag_2_Se/CuSCN/Ag	0	1200	SPLE (2‐ME)	7.39	70	65	[117]
	0.3			—	142	—	
Si/SiO_2_/Graphene/Ag_2_Se/Au	0.1	808	SPLE (Na_3_AsS_4_·8H_2_O)	—	2.878 × 10^7^	—	[118]
		1064			—	23 500	
FTO/TiO_2_/graphene/ZnS/MoO_3_/Ag	0	390	SPLE (MPA)	—	310	—	[140]
FTO/Compact TiO_2_/Mesoporous TiO_2_/CuInSe_2_/Spiro‐OMeTAD/Ag	0	330	SPLE (MPA)	—	11.3	1600	[158]
		500			3.1	440	
		780			1.5	210	
		1000			0.5	75	
ITO/ZnO/AgBiS_2_ (SPLE)/AgBiS_2_ (SSLE)/PTAA/MoO_3_/Ag	−0.5	680	SPLE (AgX, X = I and Br) SSLE (EDT)	—	153	240	[163]
		810			108	175	

### 4.2 Solar Cells

HMF chalcogenide NCs have emerged as a promising class of light‐harvesting materials for next‐generation photovoltaic devices, offering a sustainable alternative to conventional HMB NCs [166]. Depending on their composition and the device architecture, these NCs can function either as a primary photoabsorber in sensitized solar cells, as a dense NC layer in a planar solar cell, or as functional additives that enhance charge transfer and passivation in hybrid systems. To evaluate their viability, it is essential to establish their primary device architectures, working principles, and key figures of merit.

The earliest solar cell structures for HMF NCs utilized a QD‐sensitized solar cell type (QDSSC), where NCs are adsorbed onto mesoporous oxide photoanodes (typically TiO_2_ or ZnO). Upon light absorption, photogenerated electrons are injected from the NC CB into the oxide, while holes are transferred to a redox electrolyte (or a solid‐state hole conductor) to form a complete circuit. Conversely, modern high‐performance devices favor the planar heterojunction architecture. Employing NCs as a dense, standalone absorber layer requires efficient charge transport through the film, which routinely necessitates the exchange of bulky native surface ligands for shorter, more conductive alternatives. These processed NC layers were then sandwiched directly between an ETL and an HTL. The detailed categorized working principles, structural classifications, and device development pathways of CQDs solar cells are reviewed in comprehensive detail by Sargent et al. [167]

Binary HMF chalcogenide NCs, such as ZnE and Ag_2_E systems, have been implemented in several solar cell architectures depending on their functional role within the device. Among binary HMF chalcogenide system, Ag_2_Se QDs show a broad spectral coverage from the visible to the SWIR region, and the adaptation of a ZnS passivation significantly reduced surface recombination, leading to improved charge transfer and device stability in mesoporous‐type devices, with the highest PCE of 1.76% under AM 1.5G irradiation conditions [168]. Higher PCE values at lower light intensities were reported and primarily attributed to the reduction of charge carrier recombination as a result of the lower photon flux. Ag_2_S NC‐based solar cells have demonstrated significant progress through surface engineering. While traditional Ag_2_S NCs often suffer from low efficiency due to the challenging ligand exchange, recent work has utilized a thiol‐free synthesis involving heterovalent‐metal decoration (such as Bi^3+^, Ga^3+^, In^3+^, Sb^3+^, or Zn^2+^) [169]. Specifically, Ag_2_S NCs treated with TMAI achieved a PCE of 2.21%, which was further improved to over 4% by utilizing Bi‐decorated Ag_2_S NCs in a ~40 nm thick light‐absorbing layer in planar solar cells (Figure 7a,b) [169]. This enhancement is attributed to an increased electron–hole separation and prolonged exciton lifetimes facilitated by the heterovalent surface dopants [169, 171]. However, despite these advancements, solar cells based on binary material compositions often still fall behind with their relatively low efficiency compared to their lead‐based counterparts. Ternary and multinary chalcogenides have demonstrated more promising performances, narrowing the performance gap to established heavy‐metal‐containing material compositions while offering great flexibility in composition and electronic structure. For instance, AgBiS_2_ NCs have emerged as a leading HMF candidate, leveraging an exceptionally high absorption coefficient (>10^6^ cm^−1^), excellent air and moisture stability, cubic crystal structure, and sufficiently high charge transport properties within the NC film.

**FIGURE 7:**
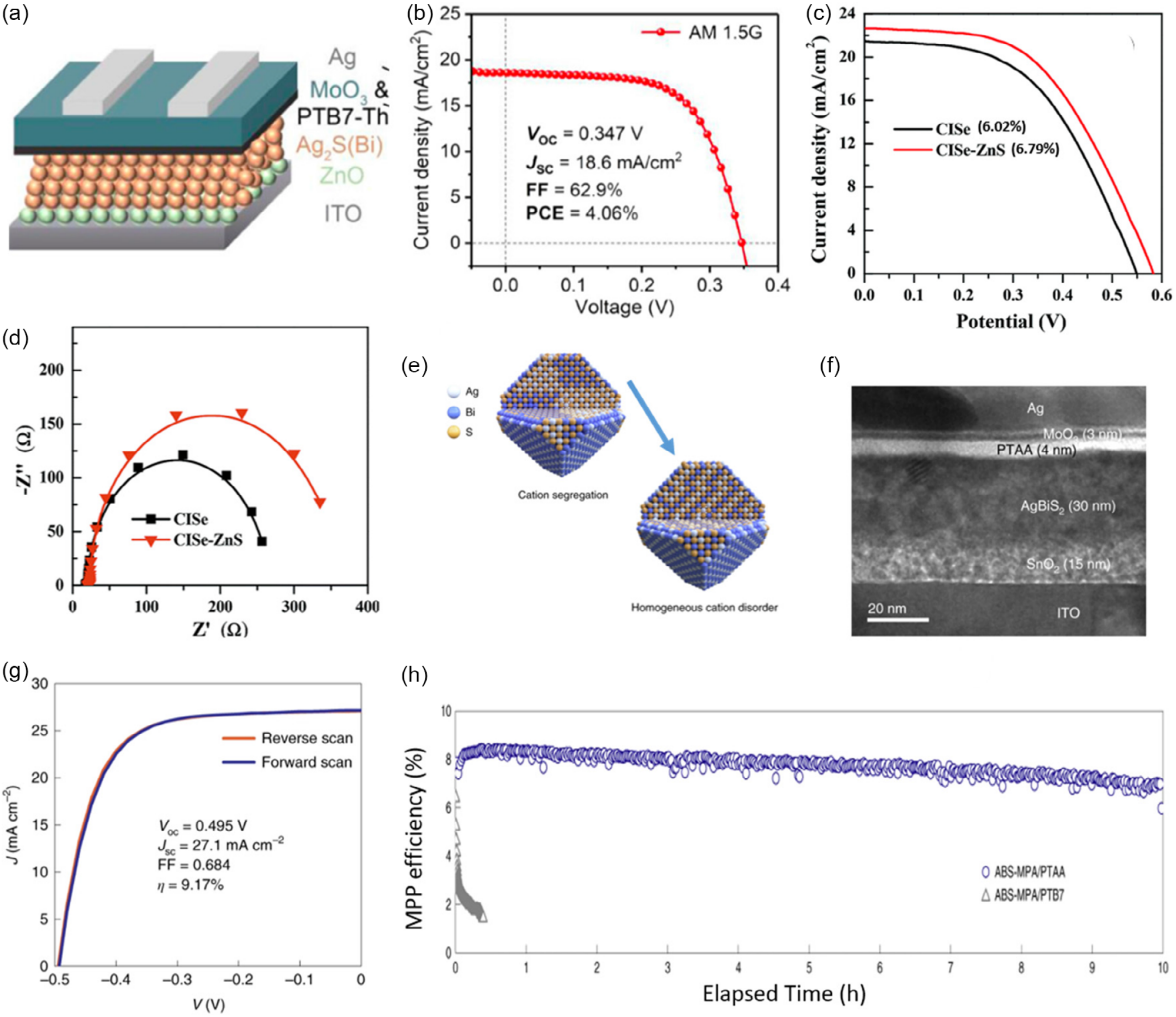
(a) Architecture of the Ag_2_S(Bi) NC‐based solar cells. (b) *J–V* characterization of the champion device. Reproduced with permission from ref. [169], Copyright 2023 American Chemical Society. (c) *J–V* curves and (d) Nyquist plots at the forward bias of −0.55 V of CISe (black) and CISe‐ZnS cells (red) champion cells. Reproduced with permission from ref. [170], Copyright 2014 Royal Society of Chemistry. (e) Schematic of AgBiS_2_ NCs with cation segregation and homogeneous cation disorder. (f) Cross‐sectional TEM image of AgBiS_2_ NCs device. (g) *J–V* curves of the champion device with PTAA as the HTL. (h) MPP test of AgBiS_2_ devices with PTB7 and PTAA as the HTL. Reproduced with permission from ref. [84], Copyright 2022 Springer Nature.

Recent advances in ternary and multinary HMF chalcogenide NCs have enabled significant improvements in device performance, particularly for systems exceeding a PCE value of 6%, where both absorber quality and interfacial engineering were simultaneously optimized [84, 170, 172, 173, 174, 175]. A foundational breakthrough in this area was demonstrated by Pan et al., who utilized ternary CuInS_2_ QDSSC to achieve a PCE of 7.04% [176]. This was accomplished by implementing effective surface passivation to form a core/shell CuInS_2_/ZnS structure, which minimized interfacial recombination while keeping the device entirely free of toxic heavy metals. Moving toward NIR‐absorbing materials for even higher theoretical efficiencies, Zhong et al. expanded the work to CuInSe_2_ (CISe) QDs [170]. The authors reported that a Cu‐poor stoichiometry (Cu/In ~ 0.7) creates beneficial cation vacancies and improves the charge separation, enabling a PCE of 6.02% (Figure 7c). This PCE was further improved to 6.79% by introducing ZnS surface passivation, which led to the formation of a CuInSe_2_–ZnS (CISe–ZnS) alloy configuration via successive ionic layer adsorption and reaction. This alloyed structure served as a highly effective light‐harvesting sensitizer by significantly reducing the charge recombination process, which was confirmed by electrochemical impedance spectroscopy (EIS) (Figure 7d). A more pronounced performance improvement has been observed in AgBiS_2_ NCs‐based solar cells, with reported efficiencies exceeding 10% in recent years. As demonstrated by Wang et al., a PCE of 9.17% (certified 8.85%) was enabled by the induction of homogeneous cation disorder (Figure 7e) within the rock‐salt lattice via low‐temperature annealing at 150 °C for an ultrathin solar cell architecture (Figure 7f) [84]. This controlled cation disorder significantly enhanced the optical absorption coefficient by approximately 5–10 times compared to conventional materials while simultaneously reducing recombination losses and directly contributing to a high *J*_sc_ of 27.11 mA cm^−2^ (Figure 7g). Furthermore, by employing a PTAA hole‐transport layer and a SnO_2_ electron‐transport layer (ETL), interfacial recombination was minimized, while 95% of the initial PCE was retained after 1000 h of ambient storage (Figure 7h).

In parallel with these interfacial refinements, the development of scalable and sustainable manufacturing routes has been further improved by the implementation of green‐processed AgBiS_2_ NC inks [173]. Wang et al. subsequently developed a green‐solvent processing route using MPA in methanol, which facilitates a clean phase transfer into environmentally friendly solvents (Figure 8a), yielding stable inks and a PCE of 7.3% (Figure 8b). These devices retained over 95% of their efficiency after 30 days of ambient exposure, highlighting the viability of nontoxic, roll‐to‐roll‐compatible fabrication [173]. To further enhance the scalability of AgBiS_2_ PVs, a solution‐phase ligand‐exchange (AgI, NaI, AgBr, and NaBr) method was introduced to passivate the (111) and (100) facets of AgBiS_2_ CQDs, which significantly reduces trap states and improves the charge extraction efficiency [172]. A distinct phase transfer is observed, wherein the AgBiS_2_ QDs migrate from a nonpolar hexane supernatant to a polar DMF subphase containing the AgI, NaI, AgBr, and NaBr salts (Figure 8c). This complete transfer indicates the successful coordination of the halide ions (I^−^ and Br^−^) and metal cations (Na^+^, Ag^+^) to the QD surface, effectively neutralizing surface charges and providing electrostatic stabilization. This mechanism results in a highly concentrated and stable colloidal ink (retaining 90% optical density over 1100 min), which is vital for the formation of the dense, pinhole‐free absorber layers required for the high‐efficiency planar heterojunction architecture (Figure 8d). The PCE of devices fabricated with these passivated CQDs reached 8.14%, representing a 53% increase compared to control devices, which had a PCE of 5.16% (Figure 8e). The passivated devices also exhibited the fastest response time of 400 ns, 2.5 times shorter than the previous best for AgBiS_2_‐based devices, showing great potential for efficient, environmentally friendly optoelectronics.

**FIGURE 8:**
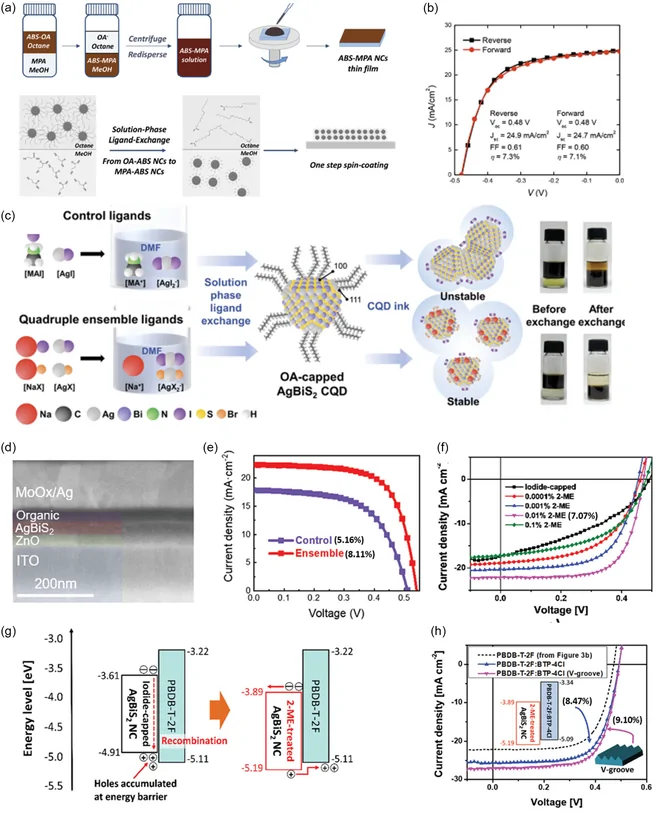
(a) Schematics of the solution‐phase ligand‐exchange process of AgBiS_2_ NCs. (b) *J–V* curves of AgBiS_2_ NCs ink‐based devices. Reproduced with permission from ref. [173], Copyright 2022 Wiley‐VCH GmbH. (c) Schematic of the ligand exchange and CQD ink‐formation methods using control ligands and a quadruple‐ligand ensemble. The images display the control (top) and ensemble ligands (bottom) before and after the solution‐phase ligand exchange. (d) Cross‐section image of the AgBiS_2_ QD device with the ensemble ligands. (e) *J–V* curves of the AgBiS_2_ QD champion cell with control and ensemble ligands. Reproduced with permission from ref. [172], Copyright 2024 Wiley‐VCH GmbH. (f) *J–V* characteristics of AgBiS_2_ NC solar cells based on the iodide‐capped NCs (black) and the 2‐ME‐treated NCs at various concentrations (red, blue, pink, and green). (g) Schematic of the energy‐level alignments of AgBiS_2_ NC and PBDB‐T‐2F before (left) and after (right) 2‐ME treatment. (h) *J*–*V* characteristics of 2‐ME‐treated AgBiS_2_ NC/PBDB‐T‐2F:BTP‐4Cl hybrid solar cells (blue) with the V‐groove textured film (pink). Reproduced with permission from ref. [174], Copyright 2022 Wiley‐VCH GmbH.

Kim et al. chose an alternative path to enhance the performance by introducing a dual organic/inorganic passivation strategy with V‐groove texturing, demonstrating a landmark PCE of 9.1% [174]. In this work, AgBiS_2_ NCs were additionally treated with 2‐ME in a solid‐state TMAI ligand exchange to be co‐passivated by iodide (I^−^) and thiolate (‐S^−^). As a result, the energy level of the AgBiS_2_ NCs absorber layer was modified and efficiently aligned with the hole transport layer (HTL), which enhanced charge extraction, leading to an improved *V*_oc_ and *J*_sc_ (Figure 8f). The study highlights the critical role of surface passivation and the importance of energy‐level matching between the AgBiS_2_ NCs and the HTL in achieving high efficiency (Figure 8g). The 2‐ME treatment increased *J*_sc_ and FF, but a higher concentration of 2‐ME resulted in decreased performance, indicating that an optimal concentration is crucial for achieving the best results. The device performance was further enhanced by incorporating BTP‐4Cl, a nonfullerene acceptor, which increased the short‐circuit current *J*_sc_ through complementary absorption in the longer‐wavelength region and improved the overall FF. Further, V‐groove texturing enhanced light trapping and optical absorption in ultrathin AgBiS_2_ layers, which, when combined with effective surface passivation and optimized energy‐level alignment, significantly contributes to achieving high efficiencies (~9.1%) in hybrid NC solar cells (Figure 8h).

Building directly upon these principles of energy‐level alignment and complementary absorption, recent breakthroughs have successfully pushed AgBiS_2_ devices past the 10% PCE milestone [38]. In particular, the implementation of a custom diketopyrrolopyrrole (DPP)‐based polymer (BD2FCT) (Figure 9a) as the HTL has enabled highly efficient vertical charge transfer by providing a well‐aligned highest occupied molecular orbital (HOMO) with the VB of AgBiS_2_ (Figure 9b). The deep HOMO levels of BD2FCT facilitated highly favorable vertical charge transfer, extracting holes to the electrode much faster than they can recombine with electrons at the interface, which drastically reduced interfacial recombination and boosted the FF. This was structurally combined with a narrow‐bandgap nonfullerene acceptor, which absorbs strongly in the NIR region. By integrating it, the device achieved true complementary absorption to harvest a much wider slice of the solar spectrum, while its energy levels created a thermodynamic driving force for charge funneling that efficiently swept electrons away from the AgBiS_2_ interfaces (Figure 9c). Together, these BD2FCT‐based AgBiS_2_ solar cells demonstrated a record PCE of 10.1% alongside significant improvements in *J*_sc_ and FF (Figure 9d).

**FIGURE 9:**
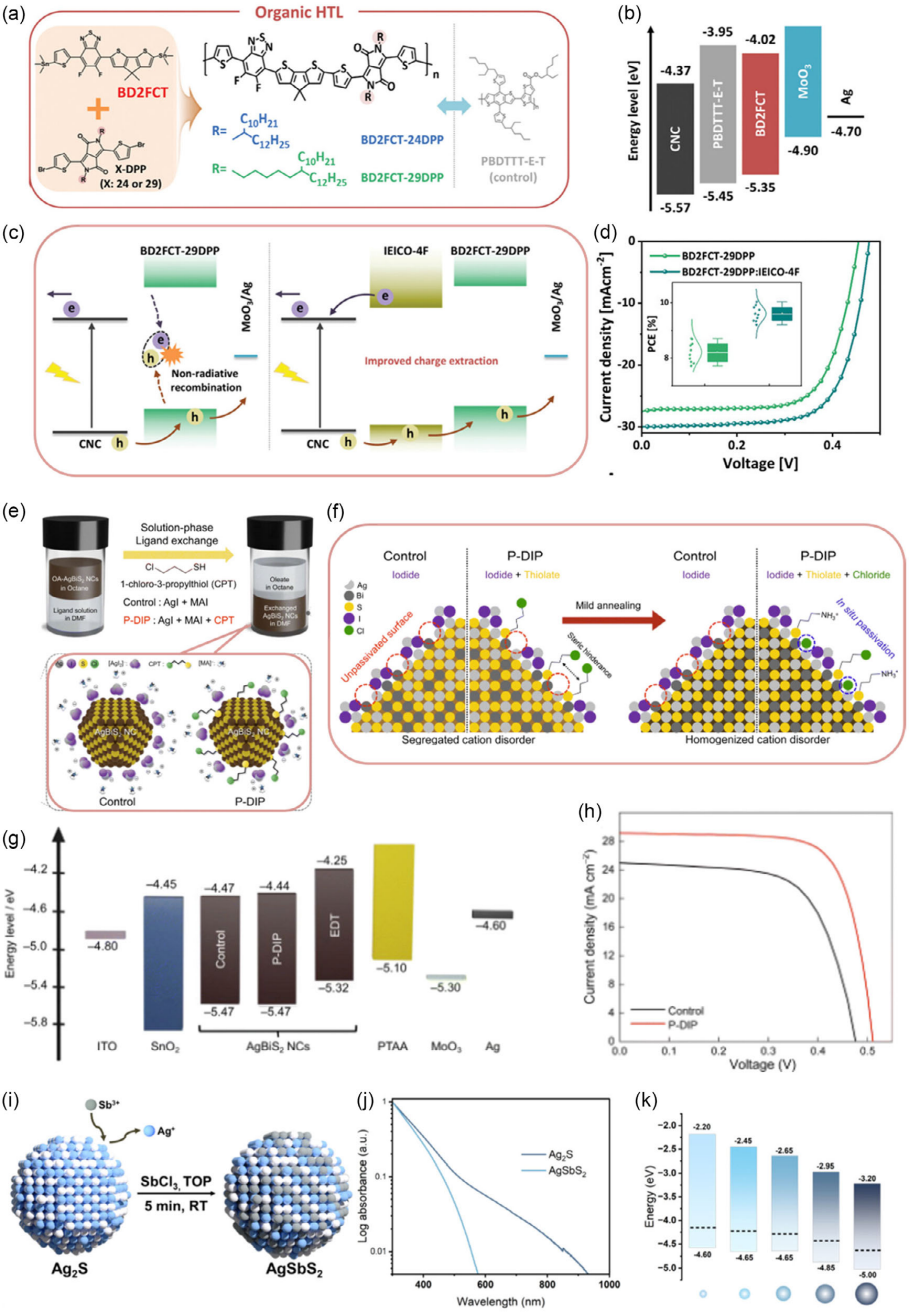
(a) Molecular structures of HTL materials used in this study, BD2FCT, and PBDTTT‐E‐T. (b) Energy levels of AgBiS_2_ NCs, PBDTTT‐E‐T, and BD2FCT. (c) Schematics illustrating charge transport in CNC/BD2FCT‐29DPP and CNC/BD2FCT‐29DPP:IEICO‐4F systems. (d) *J–V* curves, of BD2FCT‐29DPP (green) and BD2FCT‐29DPP:IEICO‐4F (cyan)‐based AgBiS_2_ NCs solar cells. Reproduced with permission from ref. [38], Copyright 2025 Wiley‐VCH GmbH. (e) Schematic of the fabrication process of control and P‐DIP AgBiS_2_ NC ink and the resulting AgBiS_2_ NCs. (f) Schematic of mild annealing‐induced in situ passivation on the P‐DIP AgBiS_2_ NCs, coupled with cation disorder homogenization. (g) Energy‐level diagram of AgBiS_2_ NC ink solar cells with different ligands. (h) *J*–*V* curves of control and P‐DIP ink solar cells. Reproduced with permission from ref. [37], Copyright 2024 Royal Society of Chemistry. (i) Schematic representation of AgSbS_2_ cation exchange synthesis at room temperature. (j) Absorbance spectra of Ag_2_S and AgSbS_2_ NCs. (k) UPS data of AgSbS_2_ NC films on SnO2 after different annealing times in solution. Reproduced with permission from ref. [47], Copyright 2026 American Chemical Society.

Concurrently, an alternative highly effective strategy was reported for AgBiS_2_ NC inks that achieves superior surface passivation via in situ dissociated chlorides [37]. Oh et al. yielded inks with exceptionally improved colloidal dispersibility and stability by introducing 3‐chloro‐1‐propanethiol (CPT) as a multifunctional molecular agent in combination with iodide ligands (Figure 9e). This advanced ink formulation leads to thin films with distinctly better morphological characteristics. Crucially, this post‐deposition in situ passivation yields NC films with significantly improved optoelectronic properties, documented by a decisively lower trap‐state density (Figure 9f) alongside increased and balanced carrier mobilities with preferable band alignment (Figure 9g), ultimately resulting in solar cells with an average PCE of 10% and an FF over 72%, and the best‐performing devices reaching a record PCE of 10.8% (Figure 9h). To eliminate the high cost and time consumption of traditional multi‐step solid‐state ligand exchanges, Ma et al. recently shifted toward direct ink synthesis frameworks [177]. This group first developed a scalable, low‐temperature solution‐phase strategy to produce electronic‐grade, pre‐passivated AgBiS_2_ NC inks, completely bypassing conventional hot‐injection routes and insulating oleic acid shells to drastically reduce material waste and carbon footprint. Next, they achieved further improvements by combining a butylamine solvent matrix with 3‐chloro‐1‐propanethiol (CPT) as bifunctional agent [178]. This strategy enabled robust chlorine‐terminated surface passivation while stripping the overall film organic content down to just 12%, which minimized inter‐nanocrystal spacing, enhanced wave‐function overlap, and ultimately propelled the power conversion efficiency of these directly synthesized AgBiS_2_ NC solar cells from 8.91% to 9.82%.

Inspired by the rapid maturation of AgBiS_2_ PVs, these scalable synthesis and passivation frameworks are now being translated to related environmentally friendly ternary chalcogenides. As illustrated in Figure 9i, the inherent difficulty of direct multinary synthesis ‐ caused by differing precursor reactivities ‐ is bypassed through a cation exchange method. Such a cation‐exchange synthesis was first developed by Senina et al. for AgBiS_2_ [179] and then also employed for AgSbS_2_ NC [47]. The transition from bismuth to antimony alters the orbital overlap and covalent character of the metal–sulfur bonds, which significantly impacts the optoelectronic properties. The log absorbance spectra (Figure 9j) highlight this shift, showing that while Ag_2_S possesses a broad NIR tail, the resulting AgSbS_2_ exhibits a much cleaner, steeper absorption edge, indicating a more defined band structure and a reduction in sub‐gap states that can plague AgBiS_2_. Furthermore, because they were synthesized as colloidal NCs, they exhibit quantum confinement, allowing precise tuning of the optical bandgap by adjusting the NC size post‐synthesis (Figure 9k). Such size‐tuned AgSbS_2_ NCs have been successfully integrated into planar solar cell architectures, demonstrating the broader versatility and potential of ternary nanocrystals for next‐generation photovoltaics.

The performance evolution summarized in Table 2 highlights a clear transition from composition‐ and ligand‐limited binary chalcogenide systems toward ternary and multinary NCs in which cation distribution, surface passivation, energy‐level alignment, and interfacial charge extraction become increasingly decisive. Beyond ternary networks, expanding into quaternary or higher HMF chalcogenide NCs like kesterite Cu_2_ZnSnS_4_ (CZTS) has been explored to achieve optimal bulk bandgaps, yet their colloidal solar cell efficiencies are characteristically below ~7% [31, 180, 181, 182]. These devices suffer exceptionally from a narrow thermodynamic stability window, where differing precursor reactivities trigger secondary phase impurities (e.g., ZnS and Cu_*x*_S) alongside extensive atomic anti‐site disorder. These crystalline mismatches generate severe local electrostatic potential and band‐edge fluctuations that act as nonradiative recombination centers, drastically limiting the device open‐circuit voltage (*V*_oc_). Therefore, modern HMF chalcogenide NCs photovoltaic research has largely consolidated around symmetric, defect‐tolerant ternary configurations like AgBiS_2_, which bypass these multi‐cation traps to secure a more practical pathway toward scalable, low‐temperature, and high‐performance green optoelectronics.

**TABLE 2:** Performance summary of representative HMF chalcogenide NC‐based solar cells.

HMF chalcogenide NC	Device type	Key surface/interface modification	PCE, %	***J***_**sc**_ **, mA/cm^2^**	***V***_**oc**_ **, V**	FF, %	References
CuInSe_2_–ZnS	QDSC	ZnS‐passivated CuInSe_2_ QD	6.79	22.61	0.583	51.5	[170]
AgBiS_2_	NC solar cell	Quadruple‐ligand ensemble multi‐facet passivation	8.11	22.33	0.540	67.0	[172]
AgBiS_2_	NC solar cell	MPA/methanol green solvent processing	7.30	24.90	0.480	61.0	[173]
AgBiS_2_	NC/Organic Hybrid	PBDB‐T‐2F:BTP‐4Cl bulk heterojunction bulk‐hybrid interface	9.10	27.07	0.490	68.0	[174]
CdSe_0.65_Te_0.35_	QDSC	Mesoporous carbon counter electrode integration	11.16	20.68	0.798	67.7	[175]
CuInS_2_/ZnS	QDSC	Core/shell structure optimization	7.04	18.21	0.707	54.7	[176]
AgBiS_2_	NC ink	Direct‐synthesis ink ligand exchange	9.32	27.67	0.487	69.2	[177]
AgBiS_2_	NC ink	Direct‐synthesis coupling design (CPT additive)	9.82	27.85	0.495	71.2	[178]
AgBiS_2_	Planar NC	Cation exchange synthesis	7.35	25.10	0.480	61.0	[179]
Cu_2_ZnSn(S_*x*_Se_1−*x*_)_4_	NC solar cell	Polar NC ink formulation	6.70	30.40	0.420	52.7	[180]

### 4.3 LEDs

An LED device consists of a p–i–n junction diode in which the emitting layer is sandwiched between the hole and electron transport layers, which are connected to the anode and cathode, respectively. Upon application of a voltage, holes and electrons are injected into the valence and conduction bands of the emitting layer. The radiative recombination of electrons and holes generate photons, whereas the emitted photon energy, and consequently, the emission wavelength or color, is determined by the energy difference between the conduction and valence bands of the emissive material. The efficiency of the emission is measured with the estimation of the following parameters: luminance spectrum, luminous flux, and current density across the device. With these factors, the efficiency of the electroluminescence is measured following the equations. To calculate EQE from the current density and luminance data, the following equations are used:

(8) $$\mathrm{EQE}=\frac{\text{Emitted photons}}{\text{Injected electrons}}$$

(9) $$\text{Emitted photons}=\frac{P_{\mathrm{opt}}\times \lambda }{hc}$$

where *h* is the Planck constant, *λ* is the emission wavelength, and *c* is the speed of light.

Now, *P*_opt_ is estimated as follows:

(10) $$P_{\mathrm{opt}}=\frac{L\times A\times \Omega }{\eta }$$

where *L* is the luminance (in Cd/m^2^), *A* is the emitting area, Ω is the solid angle in steradians, and *η* is the luminous efficacy.

On the other hand, injected electrons can be estimated from the following equation:

(11) $$\text{Injected electrons}=\frac{J\times A}{q}$$

where *J* is the current density (in mA/m^2^), *q* is the elementary charge, and *A* is the emitting area (in m^2^).

Combining all the above equations, it is apparent that the EQE is dependent on two variables, luminance and current density, as follows:

(12) $$\mathrm{EQE}\propto \frac{L}{J}$$

Equation (12) shows that achieving higher luminance at a lower current density is essential for maximizing EQE. In this sense, the current density increases with the increase of applied voltage. Therefore, a low operating voltage is necessary to mitigate the increase in current density. Photoluminescence (PL) emission properties play a critical role in determining the ultimate performance of LEDs. The PL quantum yield (PLQY) provides a direct measure of the radiative recombination of the photogenerated excitons. Radiative recombination in the emitting layer determines the emission output efficiency intrinsically and hence limits the electroluminescence efficiency. Therefore, a higher PLQY is a prerequisite for high EQE in LEDs.

NCs offer highly tunable, bright, and color‐pure emitters for efficient display technologies, often referred to as the QLED technology. Early QLEDs often relied on cadmium‐based QDs, which raised environmental and health concerns due to heavy metal toxicity. Subsequent development has focused on heavy metal‐free QLEDs to achieve comparable performance while improving safety and sustainability for commercial applications [5, 183, 184, 185, 186]. In contrast, recent progress in perovskite QDs has resulted in remarkable advances in LED performance (~26%) [187]. However, most high‐performing perovskite QDs contain lead and, consequently, fail to comply with the RoHS regulations, similar to conventional cadmium‐based chalcogenide QDs [188, 189]. Moreover, some perovskite LEDs are simultaneously struggling with long‐term stability [190]. Over the past decade, there has been a surge in QLED research, mostly focusing on the development of optimized HMF QLEDs and improving their performance to become a viable alternative to the well‐studied Cd, Hg, and Pb chalcogenides. Especially, metal chalcogenides based on Zn, Cu, Ag, In, and Ga exhibit promising performance [191]. Current research on HMF‐QDs focuses on emission wavelength tunability and spectral narrowing, which are two critical factors for achieving color purity and performance of QLEDs [192].

Among HMF chalcogenide QDs, Zn‐based systems have attracted particular attention for blue‐light‐emitting applications because of their intrinsically wide bandgaps, and consequently, high‐energy emission [193]. The high‐energy emitting Zn‐chalcogenide‐based QDs are distinguishable from many other HMF QD families, which predominantly emit in the green‐to‐red or NIR spectral regions. The blue QLEDs performance remains considerably more challenging than red and green electroluminescence due to a poor energy alignment with the adjacent transport layers and band alignment issues [194]. With a bulk bandgap of ~2.7 eV, ZnSe is intrinsically well‐positioned for color‐pure blue emission. However, reducing the QD size strengthens quantum confinement and widens the effective bandgap, driving the emission toward violet or near‐UV wavelengths, imposing a practical limitation on blue QLED implementation [195, 196, 197]. Consequently, recent progress has increasingly focused on heterostructure and alloy engineering, including ZnSe/ZnS‐ and ZnSeTe‐based architectures, to tune the emission toward the pure‐blue region while simultaneously improving carrier confinement and suppressing nonradiative surface recombination.

Alloyed Cu/Ag–In–Ga–Zn–S/Se QDs have emerged as promising alternatives to toxic HMB emitters for light‐emitting applications. Their compositional tunability enables precise control over the electronic structure and optical properties, offering advantages such as broad bandgap engineering [198, 199, 200], improved PLQY [201], and enhanced stability [202, 203, 204]. Figure 10 summarizes the reported optical performance of selected promising HMF‐alloyed QDs based on a comprehensive literature survey. In a report by Teng and coworkers [199], the authors showed that the reaction temperature is an important parameter to control the PL emission of Cu–In–Zn–S. In particular, the synthesis of multinary QDs has been synthesized composed of Cu–In–Zn–S, which was performed by varying the reaction temperature from 150 to 230 °C to study the PL effects, see Figure 10a,b, and the following effects have been obtained: (i) shifting of PL maxima from 563 to 548 nm effected from the partial cation exchange of Cu^+^ and In^3+^ with Zn^2+^, (ii) enhancement of PL intensity with the reaction temperatures increasing to 210 °C, and (iii) suppression of PL shoulder peak at 665 nm. Notably, the shoulder peak originated from the Zn‐free Cu–In–S QDs that formed at lower temperature can be considered a side product, which was successfully diminished at higher temperatures. Alongside the effect of alloying, composition tuning is also effective for bandgap tunability. In a report by Sun et al. [198], emission wavelength tunability was monitored in the synthesis of Cu–Ga–Zn–Se NCs covering the range from 524 to 603 nm by optimizing the Cu:Ga precursor molar ratios in the nucleation process, see Figure 10c. Figure 10d presents the PL spectra of I–III–VI type Ag–In–Ga–Zn–S, which showed emission tunability spanning from 470 to 614 nm by adjusting the In/Ga ratio following a one‐pot synthesis approach, reported by Xie et al. [198] Furthermore, narrowing of the emission wavelength was observed as well. Upon increasing the In/Ga ratio, increasing In^3+^ caused the decrease of the Ag^+^ and Ga^3+^ proportion concomitantly, and the disproportional ratios of Ag^+^, In^3+^, and Ga^3+^ caused the existence of Ag^+^ vacancies, promoting dominant band‐to‐hole recombination, which is conducive to narrow‐bandwidth emission.

**FIGURE 10:**
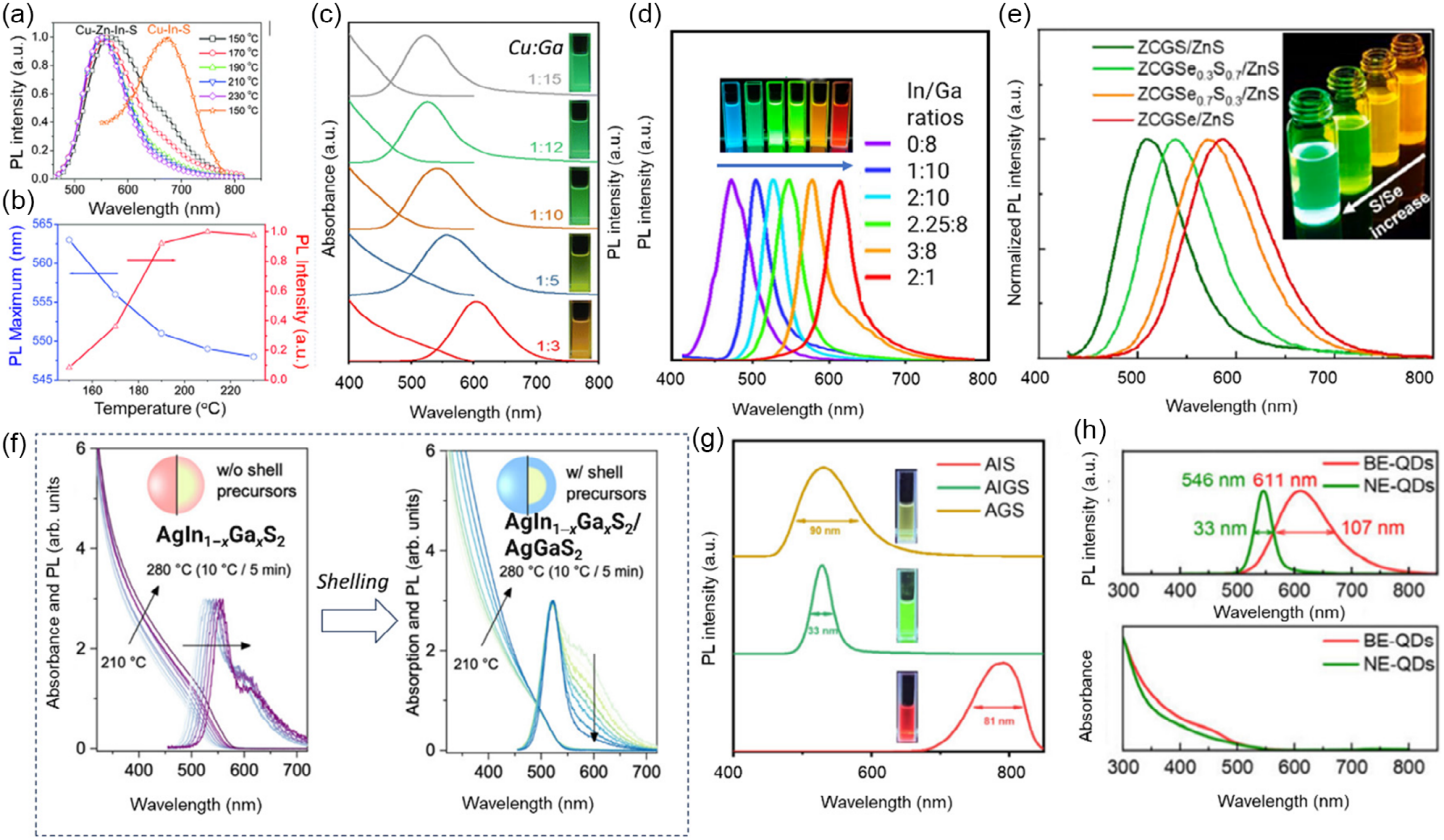
(a) PL spectra of the Cu–Zn–In–S multinary NCs synthesized at different reaction temperatures, and Cu–In–S NCs, and (b) corresponding PL peak maxima and normalized PL intensity values as a function of reaction temperature. The PL spectra were recorded at a 425 nm excitation wavelength. Reproduced with permission from ref. [199], Copyright 2015 Royal Society of Chemistry. (c) PL spectra of Cu–Ga–Zn–Se/ZnS NCs prepared at different Cu/Ga ratios, inset shows the sample emission under a 365 nm UV lamp. Reproduced with permission from ref. [200]. Copyright 2024 Royal Society of Chemistry. (d) PL spectra of Ag–In–Ga–Zn–S NCs with emission tunability at different In/Ga ratios. Reproduced with permission from ref. [198]. Copyright 2025 American Chemical Society. (e) PL spectra of Zn–Cu–Ga–Se_1−*x*_S_*x*_ showing emission tunability by altering the S/Se ratio. The inset shows the fluorescent samples under a UV lamp. Reproduced from ref. [205], Copyright 2025 Elsevier. (f) Absorbance and PL spectra of core‐only AgIn_1−*x*_Ga_*x*_S_2_ and core/shell AgIn_1−*x*_Ga_*x*_S_2_/AgGaS_2_, portray the suppression of the PL tail upon shelling. Reproduced with permission from ref. [206]. (g) Normalized PL spectra of AgInS_2_, AgInGaS_2_, and AgGaS_2_ NCs, showing bandgap narrowing upon In/Ga component engineering, and the inset shows corresponding photos of illuminated samples under a 365 nm UV lamp. Reproduced with permission from ref. [207]. Copyright 2026, American Chemical Society. (h) PL spectra (top panel) of broad‐emitting (BE) QDs and narrow‐emitting (NE) Ag–In–Ga–S QDs showing bandwidth narrowing, and their corresponding absorbance spectra. Reproduced with permission from ref. [208]. Copyright 2025 American Chemical Society.

While the previous examples highlighted the influence of cation tuning, other studies have demonstrated that emission properties can also be tuned by varying the chalcogen component. Figure 10e shows the set of PL spectra of core/shell Zn–Cu–Ga–Se_1−*x*_S_*x*_/ZnS, where the emission blueshifted with an increase of the S content [205]. The shell effect of ZnS showed improved PLQY due to the higher bandgap of the ZnS shell compared to the core material, offering a type‐I electronic structure for maximal carrier confinement, which is beneficial for light emission. The shelling effect was also found to be essential to suppress the tail emission of core/shell AgIn_1−*x*_Ga_*x*_S_2_/AgGaS_2_ QDs, as evidenced by the absorbance and PL spectra without and with shelling presented in Figure 10f. Heating at a higher temperature of the core‐only AgIn_1−*x*_Ga_*x*_S_2_ QDs caused ripening of the NCs, resulting in a redshift of the absorbance, a decrease in PLQY, and an evolution of the PL tail. Upon shelling, the absorption edge was consistently located at the same wavelength, and the tail emission was minimized with the enhanced PLQY. Apart from the emission tunability, PL spectral narrowing is necessary for QLED applications. In this regard, Cd‐free I–III–VI QDs typically show broad emission with a full‐width‐half‐maximum (FWHM) of over 90 nm, which limits the color purity in QLED devices. Therefore, significant concerns regarding PL spectral narrowing have been raised for such material systems. Ag–In–Ga–S QDs represent a rapidly developing class of HMF chalcogenide emitters [209]. In a report by Xie et al. [207], ternary AgGaS_2_ and AgInS_2_ showed broad PL emission, while incorporating both Ga and In alongside controlling the reaction procedure, resulted in narrow PL emission (Figure 10g). While the FWHM of AgGaS_2_ and AgInS_2_ are 90 and 81 nm, respectively, the Ga/In mixed QDs showed narrower PL with a FWHM of 33 nm. In a report by Yan et al. [208], uniform alloyed Ag–In–Ga–S QDs showed narrow PL emission via a defect self‐compensation effect, where thermal activation caused compensation of interstitial Ag to Ag vacancies, thereby surpressing the structural defects are significantly suppressed, hence narrowing the PL emission (Figure 10h). Li et al. demonstrated a multistep temperature‐control strategy that enabled regulated nucleation, cation exchange, and defect reconstruction, thereby minimizing the nonuniform distribution of Ag vacancies. The corresponding QLEDs achieved EQEs of 13.2%, 8.0%, and 2.9%, respectively, highlighting the critical role of defect distribution control and surface passivation in governing the optical and electroluminescent performance of multinary HMF QDs [209].

Several strategies have been employed to enhance the optical and QLED device performance through compositional engineering, surface treatment, and plasmonic coupling, among others. One of the effective approaches involves the incorporation of ZnI_2_ to passivate the QD surface to minimize surface defects [210]. As illustrated in Figure 11a–c, the introduction of ZnI_2_ significantly modifies the interfacial energy alignment and passivates surface trap states of the ZnSeTe/ZnSe/ZnS QDs, see Figure 11a. Consequently, the ZnI_2_‐treated ZnSeTe/ZnSe/ZnS QDs exhibit improved radiative recombination, which promotes stronger electroluminescence (EL) intensity compared to pristine samples, with the EQE increasing from 5.0% for the pristine QLEDs to 9.1% for the ZnI_2_‐treated QLEDs (Figure 11b). Another promising strategy for the QLED devices with improved performance involves coupling QDs with plasmonic nanoparticles through LSPR, which has been previously discussed for the improvement of PD application in Section 4.1.3 [211]. LSPR is the collective oscillation of free electrons when the particle size of metal nanoparticles (Au, in this example) is smaller. LSPR enhances the optical absorption and emission performances by amplifying the electromagnetic field around the metal nanoparticles and enhancing the emission radiation rate. Figure 11c presents a schematic of Au plasmonic nanoparticles, enabling a stronger plasmon‐exciton coupling mechanism, which enhances the excitonic emission. Figure 11d presents the absorbance spectrum of Au nanoparticles showing LSPR and the PL spectrum of ZnSeTe, which spectrally overlap in the same wavelength range. Figure 11e presents the energy band alignment of each of the layers architected for the QLED device fabrication, and the resulting device performance is presented in the plot of EQE versus current density (Figure 11f). The EQE results show that the Au nanoparticles have a significant enhancement in QLED performance, aided by the LSPR effect.

**FIGURE 11:**
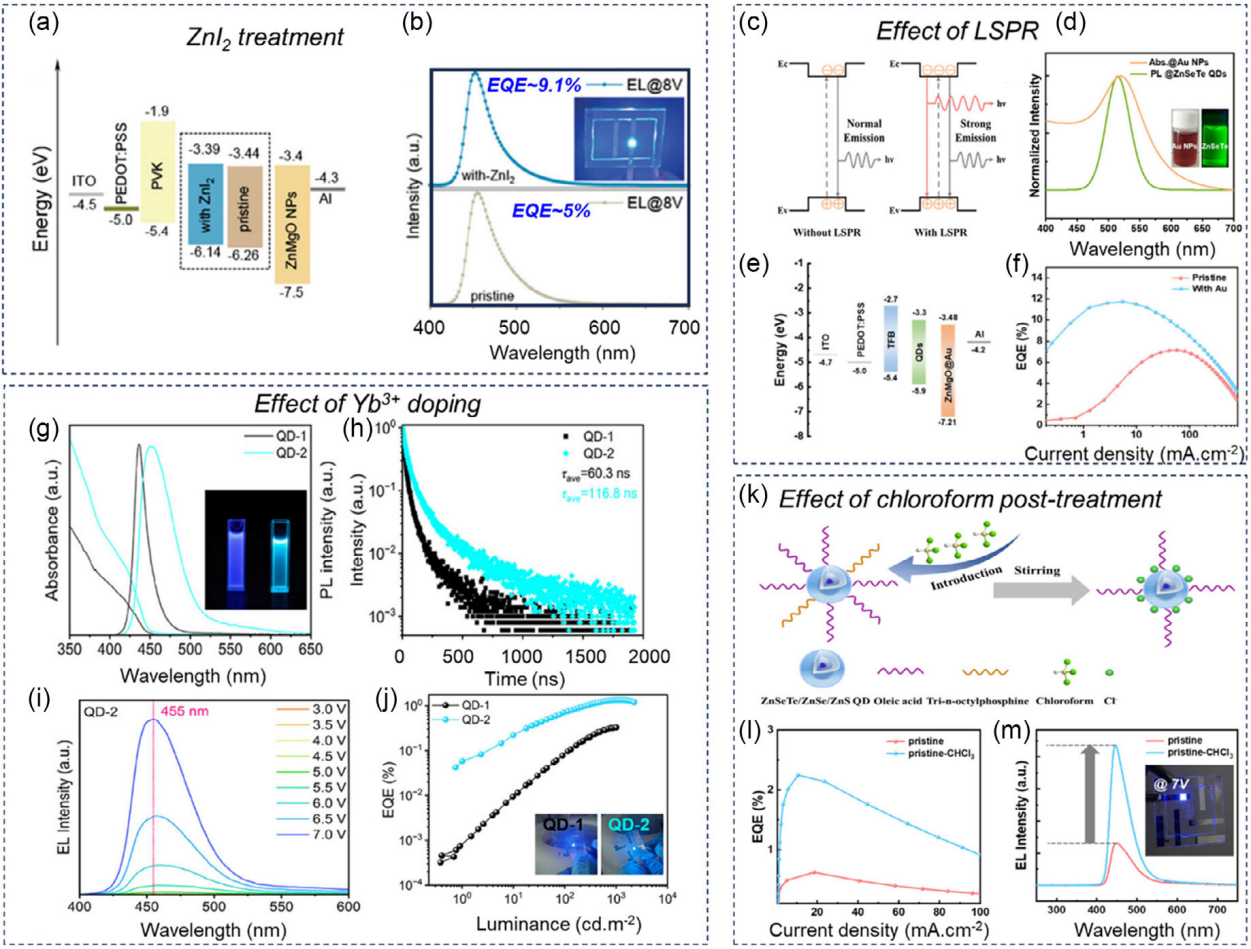
Summarizing the EL performance of QLEDs with the following effects: ZnI_2_ treatment (a) energy level diagram of as‐prepared QLED device fabricated with and without ZnI_2_ treatment, and (b) corresponding EL spectra collected at 8 V bias. Reproduced with permission from ref. [210]. Copyright 2024 Wiley‐VCH GmbH. Effect of localized surface plasmon resonance (LSPR): (c) Schematic of the LSPR effect impacting the PL emission performance of ZnSeTe QDs. (d) Normalized absorption spectrum of plasmonic Au(0) nanoparticles, and PL spectrum of ZnSeTe QDs. (e) Energy level diagram, and (f) corresponding EQE versus current density plot of the fabricated ZnSeTe QLED. Reproduced with permission from ref. [211]. Copyright 2026, American Chemical Society. Effect of Yb^3+^ doping: (g) normalized absorbance and PL spectra of undoped and Yb^3+^‐doped ZnSe/ZnS, termed as QD1 and QD2, respectively, and corresponding (h) time‐resolved PL spectra collected at 440 nm. (i) EL spectra of QD2 collected at different bias voltages, and (j) corresponding EQE versus luminance plot of QD1 and QD2 showing improved performance of Yb^3+^ doped ZnSe/ZnS over the undoped one. Reproduced with permission from [212]. Copyright 2025 Wiley‐VCH GmbH. Effect of chloroform post‐treatment: (k) schematic illustration of chloroform post‐treatment of ZnSeTe/ZnSe/ZnS QDs followed for the enhancement of optical performance. (l) EQE versus current density plots, and (m) EL spectra collected at 7 V of a blue QLED based on ZnSeTe/ZnSe/ZnS QDs with pristine and post‐chloroform treatment. Reproduced with permission from ref. [213]. Copyright 2024, American Chemical Society.

Utilizing doping to influence and control the emissive behavior of QDs and improve the QLED performance has become increasingly popular in recent years [214, 215]. In the report by Zheng and coworkers [212], Yb^3+^‐doped ZnSe/ZnS QDs have been synthesized, presenting intense pure‐blue emission centered at ~450 nm. The 2 mol% of Yb^3+^ dopants incorporated in the ZnSe core reduced the surface energy of the (220) crystal plane of ZnSe, thereby reducing interface strain in the ZnSe/ZnS structure and promoting the growth of QDs. This enables the synthesis of larger QDs with improved crystal stability. The absorbance and PL spectra of undoped (QD1) and doped (QD2) are presented in Figure 11g, and their time‐resolved PL is presented in Figure 11h. This extended lifetime suggests the suppression of nonradiative recombination processes and improved exciton stability. The EL spectra of the fabricated QLED devices under varied applied voltages are presented in Figure 11i, which shows stable emission and color purity. The corresponding EQE‐luminance characteristics are presented in Figure 11j, which shows superior device performance for doped QDs, highlighting the effectiveness of Yb^3+^ doping in enhancing the emission efficiency. In a report by Zheng et al., the post‐synthetic surface treatment of ZnSeTe QDs was studied to improve their optical performance, where the QD surface was passivated by in situ‐formed Cl^−^ ions originating from the reaction with TOP ligands present at the QD surface and CHCl_3,_ resulting in an enhancement of the PLQY from 39.6% to 92%. Furthermore, the passivation strategy also reduced the surface capping ligands, which is additionally increasing the device performance. While the surface ligands are important to stabilize the QDs, they can act as an insulating barrier. Therefore, it is necessary to reduce the surface ligands by common ligand‐exchange approaches. Figure 11k presents the schematic of the chloroform‐assisted post‐treatment process employed to introduce Cl^−^ ions onto the surface of ZnSeTe/ZnSe/ZnS QDs. The fabricated LED devices were compared with the pristine QDs highlighting the beneficial effect of the passivation [213]. Figure 11l,m present the EQE‐current density plot and EL spectra collected at a 7 V applied voltage, respectively. The passivated QDs exhibit an enhanced EL intensity and improved device efficiency compared to the pristine samples, which demonstrates that defect passivation through solvent‐mediated post‐treatment significantly enhances the charge recombination efficiency within the emitting layer. A summary of recently reported QLED devices is provided in Table 3.

**TABLE 3:** LED device performances based on heavy metal‐free QLED devices.

Material	Device structure	Emission wavelength, nm	Luminance, Cd m^−2^	EQE, %	References	Year
ZnSeTe/ZnSe/ZnS	ITO/ZnMgO/QDs/ZnMgO/Al	455	6370	9.1	[210]	2024
ZnSeTe/Au nanoparticles	ITO/PEDOT:PSS/TFB/ZnSeTe QDs/ZnMgO@Au/Al	518	82 970 at ~700 mA cm^−2^	11.75	[211]	2025
ZnSeTe	ITO/PEDOT:PSS/PF8Cz/QD/ZMO/Al	458	44 037 at 5V	18.02	[216]	2025
ZnSeTe/ZnSe/ZnS QDs	(ITO)/PEDOT:PSS/PVK/QDs emissive layer/ZnMgO nanoparticles/Al	446	710	2.25	[213]	2024
ZnSeTe	ITO/ZnO/ZnMgO/QDs/PVK/TFB/PMA/Al	461	1862	6.1	[217]	2025
ZnSeTe	ITO/PEDOT:PSS/PF8Cz/QDs/ETL/Al	456	13 670	17.5	[218]	2025
ZnSeTe/ZnSe/ZnS	ITO/PEDOT:PSS/PVK/QDs/ZnMgO/Al	443	332	5.46	[219]	2025
Cu–In–Zn–Se–S	ITO/PEDOT:PSS/TFB/NCs/Cl@ZnO:Mg/Al	663	500	4.2	[220]	2019
CuInS_2_/ZnS QLEDs	ITO/ZnO/QDs/CBP/TCTA/NPB/MoO_3_/Al	650	3000	—	[221]	2019
CuInS_2_	ITO/PEDOT:PSS/TFB/CIS‐QDs/ZnMgO/Ag	720	9	∼0.12	[222]	2025
Cu–In–Zn–S	ITO/Cu–NiO_*x*_/PEDOT:PSS/TFB/CIZS/ZnSnMgO@Cl/Al	~560	2145	6.04	[223]	2023

One of the important factors limiting the device performance of Zn‐chalcogenide‐based QLEDs is insufficient and imbalanced charge transport, making the appropriate selection and engineering of charge‐transport layers particularly important. ZnMgO is widely employed as an ETL in Zn‐based QLEDs due to its efficient electron‐transport characteristics, high optical transparency across the visible region, and suitable energy‐level alignment with the emissive QD layer [218]. Despite these advantages, its performance can be limited by poor crystallinity and associated defect states, as well as by the generation or activation of oxygen vacancies during device operation, which can promote interfacial nonradiative recombination and exciton quenching [194]. In this context, Sun et al. employed Cl^−^ ions to passivate the ZnMgO surface and subsequently introduced a Mg(OH)_2_ shell to stabilize the Cl^−^‐induced passivation. This interfacial modification suppressed nonradiative recombination and exciton quenching, thereby facilitating improved charge injection and enhanced QLED performance [218]. These findings highlight that, beyond the optimization of the emissive Zn‐chalcogenide QDs themselves, controlling the defect chemistry and interfacial electronic properties of the transport layers is equally critical for achieving efficient and operationally stable QLEDs.

### 4.4 Photocatalysis

The global energy consumption has sharply increased as a result of the ongoing expansion of the world's population, industrial activity, and living standards. Modern society has largely relied on fossil fuels, leading to environmental issues such as air pollution, climate change, and ocean acidification [224, 225, 226, 227]. Carbon dioxide (CO_2_) is the primary greenhouse gas responsible for global warming and harming ecosystems, contributing to extreme weather, melting glaciers, rising sea levels, and biodiversity loss. To address these challenges, future energy systems must be able to meet the world's expanding energy needs while also being environmentally friendly and sustainable. Solar energy stands out as an abundant and renewable resource, with the Earth receiving far more solar power than the current global demand [228, 229]. Despite this enormous availability, one of the main challenges is effectively capturing and storing solar energy in a useful and affordable way. Although photovoltaic technologies generate electricity effectively, they are limited by periodic day–night cycles and the lack of large‐scale, cost‐efficient storage [230].

Photocatalysis offers a promising alternative by directly converting solar energy into chemical energy [225, 231]. In this process, semiconductor photocatalysts absorb light to generate electron–hole pairs that drive redox reactions to propel chemical changes [231]. This enables the production of clean fuels such as hydrogen (H_2_) via water splitting, as well as the conversion of CO_2_ into value‐added products such as CO, CH_4_, and CH_3_OH and multi‐carbon compounds such as CH_3_COOH, C_2_H_5_OH, and C_2_H_2_. In addition, photocatalytic processes can facilitate ammonia synthesis through nitrogen or nitrate reduction [232, 233, 234, 235]. Beyond fuel production, photocatalysis is also widely explored for environmental remediation, where organic pollutants present in water and air can be degraded into less harmful products [236, 237]. Owing to these capabilities, photocatalysis is a promising approach for sustainable fuel production and environmental remediation. The process involves light absorption by a semiconductor to generate electron–hole pairs, followed by their separation, transport to the surface, and participation in redox reactions that drive chemical transformations. Therefore, an effective photocatalyst requires strong visible‐light absorption, efficient charge separation, good conductivity, abundant active sites, and suitable band positions to provide sufficient thermodynamic driving force to promote the targeted redox reactions. Research in photocatalytic water splitting gained significant momentum following the pioneering work of Fujishima and Honda, who demonstrated photoelectrochemical water splitting using a TiO_2_ photoanode and a platinum counter electrode under ultraviolet illumination with an applied bias [238]. Soon after, Schrauzer and Guth reported that TiO_2_ powders could drive water splitting under ultraviolet light without the need for an external electrical bias [239]. These findings spurred extensive research into inorganic semiconductor photocatalysts, particularly metal oxides, due to their stability and suitable electronic structures [240]. However, most oxide photocatalysts have large bandgaps, limiting absorption mainly to ultraviolet light, which makes up only a small fraction of the solar spectrum. This significantly reduces their overall solar utilization efficiency [241, 242]. In contrast, metal chalcogenide semiconductors possess narrower bandgaps and strong visible‐light absorption, making them more suitable for solar‐driven photocatalysis [243]. When engineered as QDs, their performance is further enhanced. Quantum confinement enables tunable bandgaps, short charge‐transport distances reduce recombination losses, and high surface‐to‐volume ratios provide more active catalytic sites. As a result, metal chalcogenide QDs are widely explored for applications such as (i) water splitting, (ii) hydrogen peroxide production, (iii) CO_2_ reduction, (iv) pollutant degradation & bacteria disinfaction, and (v) nitrate removal, highlighting their strong potential for solar fuel generation and sustainable catalysis [244, 245]. Here, we focus on the photocatalytic applications of HMF chalcogenides.

#### 4.4.1 Photocatalytic Water Splitting

Photocatalytic water splitting is considered one of the most sustainable approaches for producing green hydrogen, which has emerged as a promising alternative to fossil fuels because of its high gravimetric energy density and zero carbon emissions. The overall water splitting process consists of two half‐reactions: the hydrogen evolution reaction (HER) and the oxygen evolution reaction (OER). Thermodynamically, the reaction corresponds to a Gibbs free‐energy change of +237 kJ mol^−1^ H_2_, requiring a minimum potential of 1.23 V to proceed (Equations I–III).

(I) $$2H_{2}O+\text{sun light}↔2H_{2}+O_{2}\;\;\;\;\;\text{Δ}G=237\;\mathrm{kJ}\;{\mathrm{mol}}^{-1}$$

(II) $$4H^{+}+4e^{-}↔2H_{2}\;\;\;\;\;\;E_{\mathrm{red}}^{0}=+0.000\;V\;\mathrm{vs}.\;\text{NHE}$$

(III) $$2H_{2}O+4h^{+}↔4H^{+}+O_{2}\;\;\;\;\;\;E_{\mathrm{ox}}^{0}=+1.229\;V\;\mathrm{vs}.\;\text{NHE}$$

Therefore, semiconductor photocatalysts used for overall water splitting must possess a bandgap that can provide sufficient energy to drive this process. In practice, additional overpotential is required due to the sluggish kinetics of the OER and the complex four‐electron‐transfer process involved in water oxidation. As a result, photocatalysts with bandgaps in the range of approximately 1.8–2.4 eV are generally considered suitable for efficient solar‐driven water splitting [246]. Furthermore, the CB minimum of the photocatalyst must be more negative than the H^+^/H_2_ reduction potential, while the VB maximum should be more positive than the O_2_/H_2_O oxidation potential to ensure thermodynamic feasibility. Among various semiconductor photocatalysts, zinc sulfide (ZnS) has attracted considerable attention due to its suitable band‐edge positions and strong reduction capability. ZnS is a wide bandgap semiconductor (~3.68 eV) with a CB edge at approximately −1.36 eV, which is significantly more negative than the H^+^/H_2_ reduction potential (0 eV vs. RHE), while its VB edge (~+2.35 eV) is sufficiently positive to drive water oxidation (1.23 eV vs. RHE). These band positions suggest that ZnS is thermodynamically capable of driving photocatalytic water splitting, although its activity is mainly limited to ultraviolet irradiation because of its wide bandgap [247]. Moreover, bulk ZnS suffers from severe charge‐carrier recombination due to its short minority carrier diffusion length, which limits its photocatalytic HER activity. Reducing the particle size to form nanostructures is an effective strategy to mitigate this limitation. In nanostructured ZnS, photogenerated charge carriers need to travel a shorter distance to reach the surface and participate in catalytic reactions, thereby reducing the probability of bulk recombination and enhancing charge utilization for the HER. Jiang et al. synthesized ZnS QDs on a hydrogel (HR) substrate using a modified gel crystal growth method, producing nanoparticles with an average size of ~6 nm. The well‐dispersed ZnS/HR composite exhibited significantly enhanced hydrogen evolution activity compared to bulk ZnS, achieving a hydrogen evolution rate nearly fifteen times higher under UV–vis irradiation in the presence of Na_2_S and Na_2_SO_3_ as sacrificial reagents [248].

Nevertheless, the photocatalytic HER performance of ZnS QDs remains insignificant due to the large bandgap, poor conductivity, and insufficient intrinsic catalytic activity of ZnS. To overcome these limitations, several strategies, such as elemental doping and heterostructure engineering, have been explored. For instance, Poornaprakash et al. demonstrated that doping ZnS QDs with Eu^3+^ (6%) reduces the bandgap from 3.6 to 3.39 eV due to sp‐d/sp‐f exchange interactions between the band electrons and localized f‐orbital electrons of Eu^3+^ within the ZnS lattice [249]. This bandgap modulation significantly enhanced the photocatalytic activity, increasing the hydrogen evolution rate to 9000 μmol g^−1^ h^−1^ compared to 3800 μmol g^−1^ h^−1^ for pristine ZnS QDs. Although nanostructure formation can effectively mitigate interfacial carrier recombination by reducing the distance associated with minority carrier diffusion, bulk carrier recombination remains a major factor limiting the photocatalytic performance. Constructing heterojunction QDs represents an effective strategy to suppress carrier recombination by promoting efficient charge separation and transfer, thereby improving charge carrier dynamics and ultimately enhancing photocatalytic activity. Despite significant research efforts, the photocatalytic HER performance of ZnS QD‐based systems remains limited under visible‐light irradiation owing to their wide bandgap. Therefore, research has extended beyond ZnS‐based systems toward narrow‐bandgap metal chalcogenide QDs, which offer enhanced visible‐light absorption and have been investigated as promising photocatalysts for solar‐driven hydrogen generation. For example, Yu et al. reported Ag_2_S QDs as infrared‐responsive photocatalysts capable of producing H_2_ under long‐wavelength irradiation (Figure 12) [250]. Through a solvothermal synthesis approach, the band structure of Ag_2_S QDs was tuned to facilitate electron transfer to hydrogen ions. Ag_2_S QDs with an absorption peak at 800 nm exhibit a Tafel slope of 89 mV dec^−1^ and an overpotential of 0.32 V in an electrocatalysis test, demonstrating their catalytic potential toward the HER. When used as photocatalysts, these QDs generated hydrogen at a rate of 858 μmol g^−1^ h^−1^ under white light irradiation and remained active even under NIR excitation (800 ± 20 nm), representing one of the longest excitation wavelengths reported for semiconductor‐driven hydrogen generation.

**FIGURE 12:**
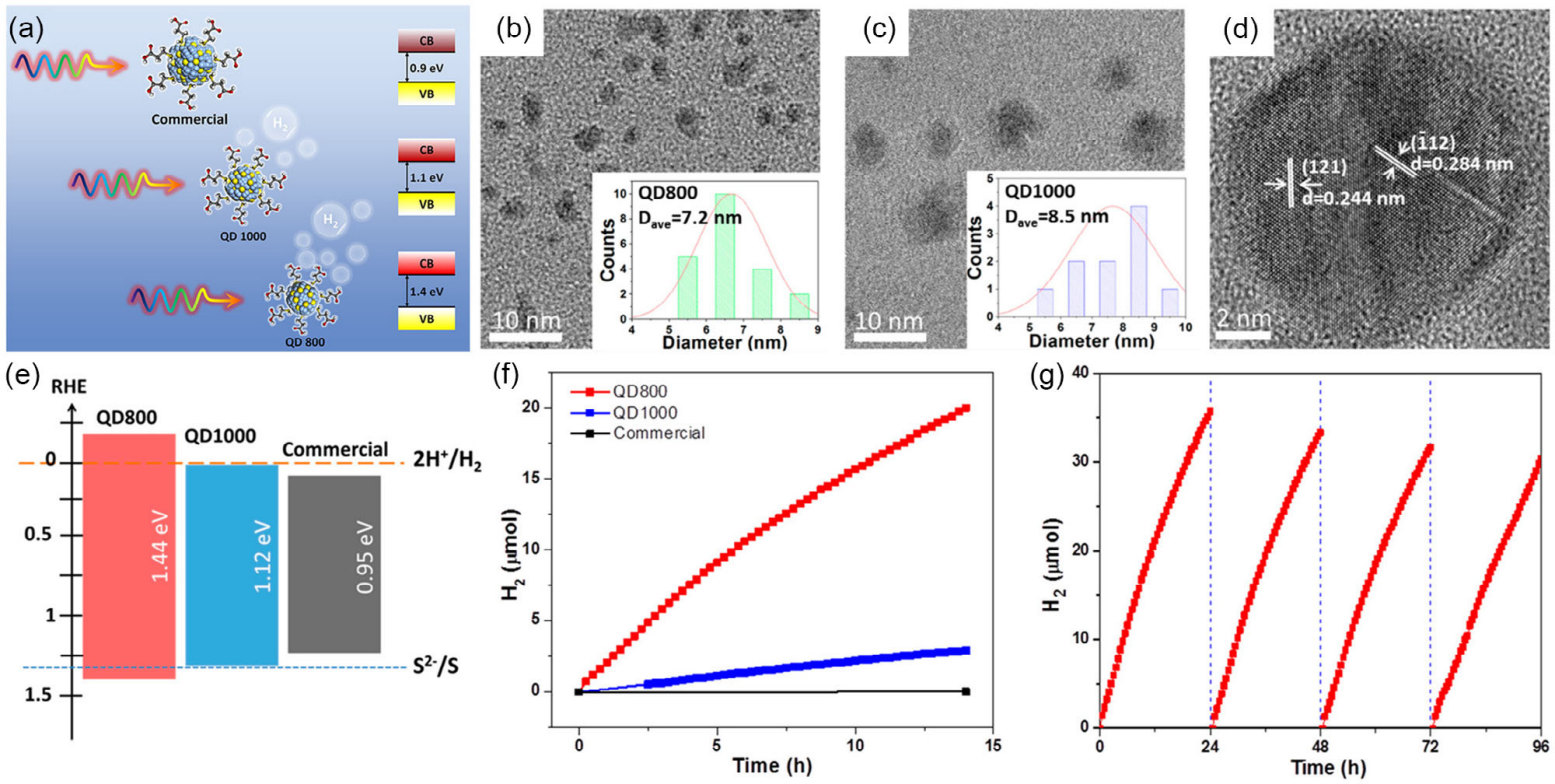
(a) Schematic illustration of commercially available Ag_2_S, Ag_2_S QDs with absorption peaks at 800 nm (QD 800) and absorption peak at 1000 nm (QD 1000). TEM images of (b) QD800 and (c) QD1000. Insets: average diameter distribution of the QDs. (d) HR‐TEM image of an Ag_2_S QD. (e) band energy diagram and (f) Photocatalytic H_2_ evolution test of the QDs. (g) Stability tests for Ag_2_S QD800s dots. Reproduced with permission from ref. [250]. Copyright 2019, American Chemical Society.

Heterojunction approaches are also adopted for tuning the catalytic properties of Ag_2_S QDs. You et al. developed a 0D/2D heterostructure by anchoring Ag_2_S QDs onto g‐C_3_N_4_ nanosheets through a self‐assembly strategy [251]. The Ag_2_S QDs with an average size of ~5.8 nm were uniformly dispersed on the surface of g‐C_3_N_4_, improving charge transfer and extending light absorption. The optimized composite containing 0.5 wt% Ag_2_S QDs achieved a hydrogen evolution rate of 471.1 μmol g^−1^ h^−1^ with an apparent quantum efficiency of 1.48% at 405 nm, outperforming both pristine g‐C_3_N_4_ and Pt‐loaded g‐C_3_N_4_ systems. The enhanced performance was attributed to quantum confinement effects and efficient interfacial charge transfer between the two components. Similarly, Fan et al. constructed another 0D/2D heterostructure by anchoring 0D Ag_2_S QDs onto 2D graphdiyne (GDY) nanosheets for wide‐spectrum photocatalytic hydrogen evolution [252]. This architecture empowered strong interfacial contact between Ag_2_S QDs and the GDY, enabling rapid electron transfer. The composite exhibited broad light absorption extending up to 1200 nm. Density functional theory calculations and in situ XPS analysis revealed that the incorporation of Ag_2_S QDs modifies the electronic structure of GDY, generating an internal electric field that promotes directional electron transfer. Consequently, a 7 wt% Ag_2_S QDs/GDY composite achieved an impressive hydrogen evolution rate of 2418 μmol g^−1^ h^−1^, significantly higher than that of the pristine materials. In addition to monometallic chalcogenide QDs, multimetallic chalcogenide QDs have also been explored due to their tunable electronic structures and synergistic catalytic properties, including enhanced charge transport, adjustable band structures, and improved catalytic activity. For example, Li et al. investigated CuInS_2_ (CIS) QDs and demonstrated that defect engineering plays a crucial role in improving carrier separation and transport [253]. Shallow defects can trap and transfer carriers efficiently, whereas deep defects promote nonradiative recombination. By controlling the nucleation process and passivating deep defects with a ZnS shell, CIS/ZnS QDs exhibited significantly improved carrier dynamics. Time‐resolved PL studies revealed that coating with ZnS eliminates recombination pathways associated with deep defect states. As a result, the CIS/ZnS QDs achieved a superior hydrogen production rate of 35.6 mmol g^−1^ h^−1^, which is nearly 240 times higher than that of the conventional CIS QDs. Beyond bimetallic systems, trimetallic chalcogenide QDs have also demonstrated remarkable photocatalytic performance due to their tunable composition and band structures. Yuan et al. reported composition‐controlled (AgIn)_*x*_Zn_2(1−*x*)_S_2_ QDs as efficient photosensitizers for hydrogen generation [254]. By varying the composition from ZnS (*x* = 0) to AgInS_2_ (*x* = 1), the bandgap could be tuned from 3.55 to 1.80 eV. An optimal balance between light absorption and the redox driving force was achieved for (AgIn)_0.5_ZnS_2_ QDs, which exhibited the highest photocatalytic performance with an apparent quantum yield of 8.2% under monochromatic illumination at 450 nm. This study demonstrates that bandgap engineering through compositional tuning provides an effective strategy for designing efficient and low‐toxicity QD photosensitizers for solar hydrogen production.

In addition to freshwater splitting, photocatalytic seawater splitting has gained increasing attention due to its practical relevance and abundant resources. For this purpose, Chang et al. developed nitrogen‐doped carbon dots (N‐CDs) coupled with indium‐doped ZnS heterostructures as a photocatalyst for hydrogen production from seawater [255]. The incorporation of N‐CDs improved light absorption and facilitated charge separation within the heterostructure. Under visible light irradiation, the heterostructure photocatalyst exhibited significantly enhanced hydrogen evolution rates in seawater, which were approximately 1.83 times higher than those obtained in deionized water. This improvement was attributed to the higher ion concentration in seawater, which suppresses charge recombination and enhances carrier mobility. Furthermore, the photocatalyst demonstrated excellent stability, retaining more than 90% of its activity after five cycles. These results highlight the potential of doped ZnS‐based heterostructured photocatalysts for sustainable hydrogen production from seawater.

Overall, QDs have emerged as highly promising photocatalysts for solar‐driven water splitting due to their tunable band structures, strong light absorption, and enhanced charge carrier dynamics arising from quantum confinement effects. Through strategies such as compositional engineering, elemental doping, defect modulation, and heterostructure construction, the photocatalytic performance of QDs can be significantly improved. Therefore, continued research on QD‐based photocatalysts is expected to play a crucial role in the development of efficient and sustainable systems for future solar hydrogen production.

#### 4.4.2 Photocatalytic H_2_O_2_ Production

Hydrogen peroxide (H_2_O_2_) is regarded as an environmentally benign oxidizing agent because of its high oxidation potential and excellent atom efficiency. Owing to these advantages, it has been widely applied in chemical synthesis, sterilization, wastewater treatment, bleaching processes, and Fenton‐based advanced oxidation reactions. Currently, global H_2_O_2_ production exceeds several million tons annually, with more than 95% of the supply being generated through the conventional anthraquinone process. However, this industrial method is energy‐intensive and associated with considerable carbon emissions, releasing nearly 1.2 tons of CO_2_ for every ton of H_2_O_2_ produced [256]. These environmental concerns have stimulated extensive research toward sustainable and green routes for H_2_O_2_ generation.

Photocatalytic production of H_2_O_2_ has emerged as a promising alternative because it enables the direct conversion of water and molecular oxygen into H_2_O_2_ using solar energy. In general, photocatalytic H_2_O_2_ synthesis proceeds through two major pathways: the oxygen reduction reaction (ORR) and the water oxidation reaction (WOR). In a typical semiconductor photocatalytic system, photogenerated electrons in the CB reduce adsorbed O_2_ molecules, while the holes in the VB participate in oxidation reactions. For the ORR pathway, H_2_O_2_ can be produced either through a direct two‐electron reduction of O_2_ (O_2_ → H_2_O_2_) or through a stepwise mechanism involving single‐electron transfers (O_2_ → 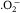 → H_2_O_2_) (Equations (5), (6), (7), (8)). Among these pathways, the direct two‐electron ORR generally exhibits higher selectivity toward H_2_O_2_ formation, whereas the stepwise route generates reactive intermediates such as the hydroperoxyl radical (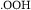), which may limit H_2_O_2_ accumulation. Thermodynamically, the direct two‐electron ORR is favorable with a potential of +0.68 V versus the normal hydrogen electrode (NHE), although the stepwise route can be kinetically advantageous due to sequential single‐electron‐transfer steps [256].



(IV)

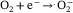






(V)

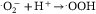






(VI)

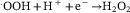

(VII) $$O_{2}+2H^{+}+2e^{-}\to H_{2}O_{2}$$

(VIII) $$2H_{2}O+4h^{+}\to O_{2}+4H^{+}$$

(IX) $$2H_{2}O+2h^{+}\to H_{2}O_{2}+2H^{+}$$

(X)

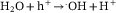






(XI)

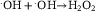




In addition to the ORR, oxidation reactions at the VB can also contribute to H_2_O_2_ formation (Equations 9–12). Photogenerated holes may oxidize water to produce O_2_ and protons through the four‐electron water oxidation pathway. Alternatively, holes can participate in the two‐electron oxidation of water to form H_2_O_2_ directly. In certain photocatalysts, holes can also generate hydroxyl radicals (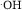) through a single‐electron water oxidation step, and the recombination of these radicals subsequently produces H_2_O_2_. However, this pathway requires a high concentration of 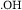 radicals and is therefore less efficient. Furthermore, from a thermodynamic perspective, the four‐electron WOR (1.23 V vs. NHE) is more favorable than the two‐electron pathway leading to H_2_O_2_ formation (1.78 V vs. NHE), making the direct two‐electron WOR relatively difficult [256]. Consequently, developing efficient photocatalysts that can selectively drive these reactions has become an important research focus.

Recent studies have demonstrated that the rational design of semiconductor photocatalysts can significantly enhance H_2_O_2_ generation. Semiconductor photocatalysts like TiO_2_, ZnO, ZnS, CdS, etc. are primarily explored for H_2_O_2_ production. Among them, the photocatalytic performance of TiO_2_, ZnO, and ZnS is limited under visible light owing to their large bandgap, while visible‐light‐active CdS is toxic to the environment. In this regard, metal chalcogenides have emerged as promising photocatalysts due to their attractive semiconducting properties and environmental friendliness. Zhang et al. reported size‐controlled ZnSe NCs synthesized through a solvothermal approach by adjusting the concentrations of the Se precursor and KBH_4_ [257]. The photocatalytic activity was found to be strongly dependent on the nanocrystal size. Under oxygen bubbling conditions, the optimized ZnSe catalyst achieved an H_2_O_2_ production rate of 8.596 mmol g^−1^ h^−1^ with an apparent quantum efficiency of 2.84% at 420 nm. Even under air‐bubbling conditions, a considerable H_2_O_2_ concentration of 1.758 mmol L^−1^ was obtained after 3 h of irradiation, demonstrating a performance significantly higher than commonly studied photocatalysts such as TiO_2_, g‐C_3_N_4_, and ZnS. The efficiency of photocatalytic H_2_O_2_ production can also be significantly enhanced through the rational construction of heterostructures, which facilitate efficient charge separation and transport. Although metal chalcogenides possess favorable optoelectronic properties for photocatalytic applications, their limited number of accessible active sites and relatively low exposed surface area remain major challenges for efficient H_2_O_2_ production. Metal–organic frameworks (MOFs), in contrast, have emerged as promising semiconducting materials owing to their high‐surface areas, abundant accessible active sites, tunable band structures, and good structural stability. Therefore, constructing heterojunctions between MOFs and metal chalcogenides represents an effective strategy for developing efficient photocatalysts that combine strong visible‐light absorption with enhanced charge separation, carrier transport, and abundant catalytically active sites. Building on this rationale, Liu et al. developed an NH_2_‐MIL‐125/ZnS heterojunction through the selective sulfidation of NH_2_‐MIL‐125/ZIF‐8 hybrids. In this system, ZnS nanocages are preferentially anchored at the corners of disc‐shaped NH_2_‐MIL‐125 crystals, forming well‐aligned band structures at the interface [258]. By controlling the ZIF‐8/ZnS size ratio, the heterojunction coverage could be optimized to regulate light absorption and charge separation. The optimized composite with ~45.1% junction coverage exhibited a H_2_O_2_ generation rate of about 120 mM g^−1^ h^−1^, demonstrating a 370% improvement compared with pristine NH_2_‐MIL‐125.

Covalent organic frameworks (COFs) represent a versatile family of crystalline porous polymers with considerable potential as semiconductor materials for optoelectronic and photocatalytic applications. Their well‐defined porous architectures, high chemical stability, tunable electronic and optical characteristics, large accessible surface areas, and structurally programmable synthesis provide opportunities for tailoring their charge‐transport and light‐harvesting properties. Owing to these advantages, coupling COFs with metal chalcogenides offers a promising approach for constructing heterojunction photocatalysts, where interfacial interactions can facilitate photogenerated charge separation and transport while providing abundant accessible catalytic sites. Liu and coworkers constructed an S‐scheme heterojunction by decorating AgInS_2_ QDs on a flower‐like covalent organic T‐COF [259]. Strong redox potentials were maintained, while effective charge separation was made possible by the resulting AgInS_2_/T‐COF composite. Consequently, without the use of sacrificial agents, the optimized photocatalyst produced H_2_O_2_ at a rate of 2768 μmol g^−1^ h^−1^, which was much higher than that of the individual components. The S‐scheme heterojunction promoted effective interfacial charge transfer and improved photocatalytic performance, according to mechanistic studies that included radical trapping experiments, in situ diffuse reflectance infrared spectroscopy, femtosecond transient absorption spectroscopy, Kelvin probe force microscopy, and in situ X‐ray photoelectron spectroscopy. Furthermore, the alloyed QDs have also garnered a lot of interest for photocatalytic H_2_O_2_ production because of their tunable electronic structures. Nonstoichiometric Zn–Cu–In–S (ZCIS) quaternary alloyed QDs for visible light‐driven H_2_O_2_ generation and ciprofloxacin degradation were reported by Prusty and colleagues using a reflux method (Figure 13a–d) [260]. With a solar‐to‐chemical conversion efficiency of 0.19% and an H_2_O_2_ production rate of 1685.2 μmol g^−1^ h^−1^, the optimized ZCIS catalyst outperformed CuInS_2_ QDs by approximately 5.3 times. Enhanced exciton separation, broad light absorption, appropriate band alignment, and effective oxygen activation were identified as the reasons for the improved performance. By introducing both cation and anion alloying, compositional engineering made further progress. To modify their optoelectronic characteristics, ternary Cu–In–S (CIS), quaternary Zn–Cu–In–S (ZCIS), and quinary Zn–Cu–In–S–Se (ZCISSe) QDs were created in a later investigation (Figure 13e–h) [261]. Among them, the quinary ZCISSe QDs exhibited optimized light absorption and charge carrier transport characteristics, resulting in a pronounced H_2_O_2_ production rate of 2565.5 μmol g^−1^ h^−1^ with a solar‐to‐chemical conversion efficiency of 0.36%. This activity was nearly twice that of ZCIS and approximately eight times higher than that of CIS QDs. In addition, the material demonstrated excellent efficiency for the degradation of organic micropollutants under visible‐light irradiation.

**FIGURE 13:**
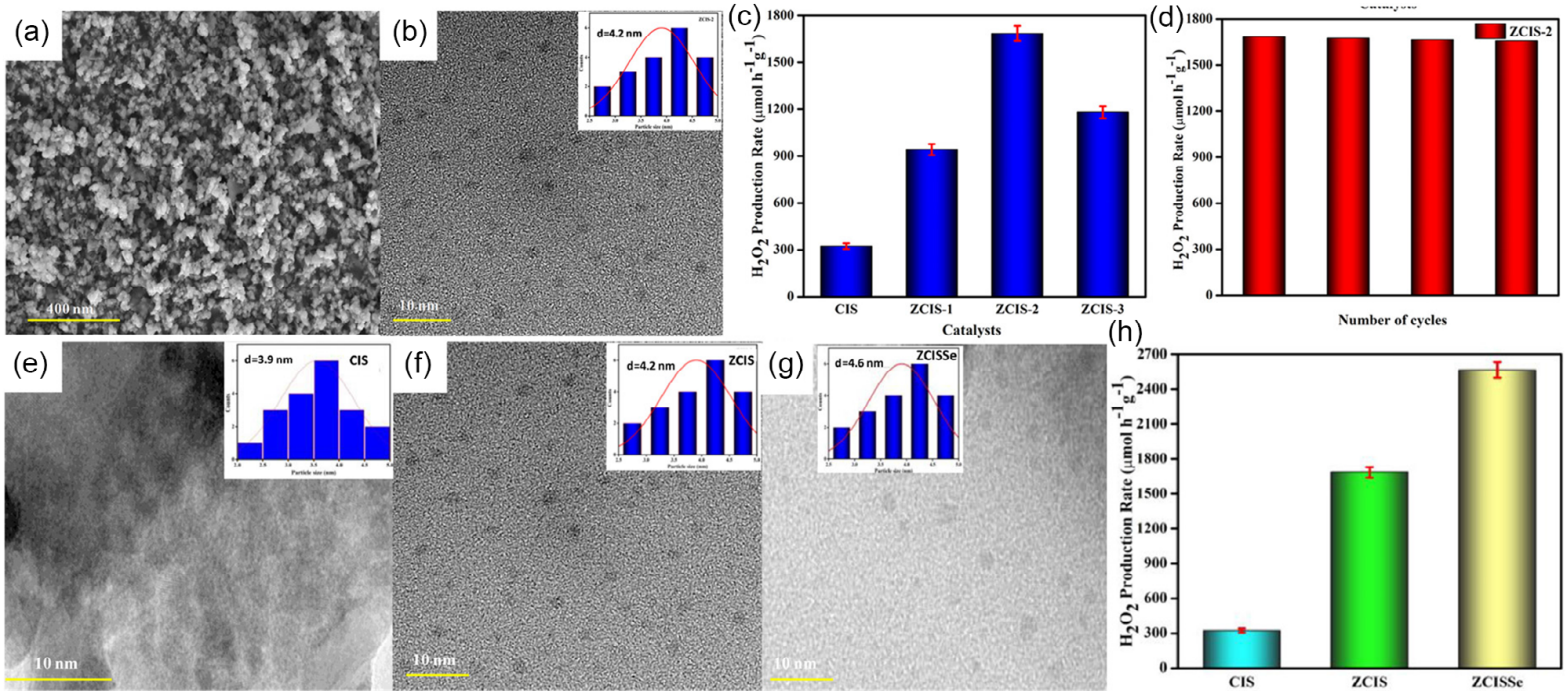
(a) FESEM and (b) HRTEM images of Zn–Cu–In–S (ZCIS, Cu/Zn 1:2) alloyed QDs. (c) H_2_O_2_ production rate of CIS (Cu/Zn 1:0), ZCIS 1 (Cu/Zn 1:1), ZCIS 2 (Cu/Zn 1:2), ZCIS 3 (Cu/Zn 1:3) QDs. (d) Photocatalytic stability test of ZCIS 2 QD. Reproduced with permission from ref. [260], Copyright 2022, American Chemical Society. HRTEM images of (e) CIS, (f) ZCIS, and (g) ZCISSe QDs, respectively. (h) Photocatalytic H_2_O_2_ production rate of CIS, ZCIS, and ZCISSe QDs. Reproduced with permission from ref. [261]. Copyright 2023, Elsevier.

Overall, these studies highlight that strategies such as size control, heterojunction engineering, and compositional tuning of semiconductor QDs are highly effective for improving photocatalytic H_2_O_2_ generation. The continued development of advanced materials with optimized electronic structures and charge‐transfer properties is expected to further advance solar‐driven H_2_O_2_ production technologies.

#### 4.4.3 Photocatalytic CO_2_ Reduction

The continuous increase in atmospheric CO_2_ concentration has become a major environmental challenge, prompting extensive research efforts toward converting CO_2_ into valuable chemicals and fuels. Among the various strategies, solar‐driven photocatalytic CO_2_ reduction has emerged as a promising approach because it utilizes abundant solar energy to transform CO_2_ into useful products. This process is analogous to natural photosynthesis, where plants convert CO_2_ and H_2_O into carbohydrates and O_2_ under sunlight. In photocatalytic systems, semiconductor materials absorb photons with energies equal to or greater than their bandgap, generating electron–hole pairs. The photogenerated electrons in the CB reduce adsorbed CO_2_ molecules into carbon‐based products, while the holes in the VB oxidize water to produce O_2_.

(XII) $${\mathrm{CO}}_{2}+2H^{+}+2e^{-}\to \text{HCOOH }E^{0}=-0.61\;V$$

(XIII) $${\mathrm{CO}}_{2}+2H^{+}+2e^{-}\to \mathrm{CO}+H_{2}O\;\;\;\;\;\;E^{0}=-0.53\;V$$

(XIV) $${\mathrm{CO}}_{2}+4H^{+}+4e^{-}\to \text{HCHO}+H_{2}O\;\;\;\;\;E^{0}=-0.48\;V$$

(XV) $${\mathrm{CO}}_{2}+6H^{+}+6e^{-}\to {\mathrm{CH}}_{3}\mathrm{OH}+H_{2}O\;\;\;\;E^{0}=-0.38\;V$$

(XVI) $${\mathrm{CO}}_{2}+8H^{+}+8e^{-}\to {\mathrm{CH}}_{4}+2H_{2}O\;\;\;\;\;E^{0}=-0.24\;V$$

Carbon in CO_2_ exists in its highest oxidation state; therefore, its reduction can yield a variety of products, such as CO, CH_4_, CH_3_OH, and HCOOH, depending on the number of transferred electrons and protons (Equations (I), (II), (III), (IV), (V)). The product distribution is governed by both thermodynamic and kinetic factors. For instance, the reduction of CO_2_ to CH_4_ (−0.24 V vs. NHE at pH 7) is thermodynamically more favorable than the formation of CO (−0.53 V vs. NHE), although the actual reaction pathway involves multiple intermediate steps. The general photocatalytic process involves several stages, including adsorption of reactants on the catalyst surface, photoactivation by charge carriers, formation of reaction intermediates, transformation of intermediates to final products, desorption of products, and regeneration of the catalyst. Because these reactions proceed through complex multistep intermediates, controlling reaction kinetics and selectivity remains a major challenge in photocatalytic CO_2_ reduction.

QDs offer size‐dependent tunable bandgaps and band‐edge positions, enabling better alignment with the redox potentials required for efficient CO_2_ reduction. Additionally, their high surface‐to‐volume ratio and short charge diffusion length enhance light absorption, charge separation, and catalytic activity compared to conventional bulk photocatalysts. Significant progress has been achieved by designing quantum‐dot‐based photocatalysts that offer tunable electronic structures and efficient charge separation. QD‐based photocatalysts offer tunable electronic structures and efficient charge separation. However, the lack of redox‐active catalytic sites, insufficient stabilization of reaction intermediates, and poor product selectivity remain major challenges in photocatalytic CO_2_ reduction. Conventional semiconductor photocatalysts often require integration with expensive noble‐metal‐based catalysts to achieve efficient CO_2_ conversion. In this context, coupling metal chalcogenide QDs with noble‐metal‐free molecular catalysts represents an attractive strategy as it can facilitate efficient charge separation and suppress carrier recombination while providing abundant redox‐active sites for CO_2_ activation and selective reduction. For example, Kuehnel and coworkers developed a noble‐metal‐free photocatalytic system consisting of ligand‐free ZnSe QDs coupled with a phosphonic‐acid‐functionalized Ni(cyclam) molecular catalyst [262]. In this hybrid system, ZnSe QDs act as visible‐light photosensitizers, while the Ni(cyclam) complex serves as the catalytic center for CO_2_ reduction. The catalyst exhibited efficient CO_2_ conversion to CO in aqueous media with a turnover number exceeding 120. When the ZnSe surface was further modified with 2‐(dimethylamino)ethanethiol (MEDA), hydrogen evolution was partially suppressed, resulting in enhanced CO production with a turnover number greater than 280 and a CO_2_ reduction selectivity exceeding 33% under visible‐light irradiation. The system also achieved an external quantum efficiency of approximately 3.4% at 400 nm, comparable to several precious‐metal‐based photocatalysts. Surface ligand engineering has also been demonstrated as an effective strategy to tune catalytic selectivity. Sahm and coworkers reported that modifying ZnSe QDs with thiolated imidazolium ligands significantly enhances photocatalytic CO_2_ reduction even in the absence of transition‐metal cocatalysts [263]. After 10 h of exposure to visible light, the imidazolium‐modified ZnSe QDs produced CO up to 2.4 mmol g^−1^ with a CO selectivity of roughly 20%, which is a significant improvement over unmodified ZnSe. Strong ligand binding to the quantum‐dot surface was revealed by spectroscopic analysis, and transient absorption spectroscopy showed that the ligand affects charge‐carrier dynamics and passivates surface defects. Additionally, calculations using density functional theory revealed that the imidazolium ligand stabilizes the surface‐bound CO_2_^−^ intermediate, increasing production yield and selectivity of CO. Sahm et al. later investigated the use of dithiol ligands to control the catalytic properties of ZnSe QDs, building on this idea of surface chemical environment engineering [264]. It was observed that dithiols, which had not gotten much attention in CO_2_ photoreduction studies before, had a major impact on the catalytic performance. Short‐chain dithiols, like 1,2‐ethanedithiol, suppress the competing HER while activating the QDs surface for CO_2_ reduction. On the other hand, when a Ni(cyclam) cocatalyst is present, longer dithiols like 1,6‐hexanedithiol improve CO_2_ reduction. Strong interactions between dithiol ligands and the quantum‐dot surface were confirmed by in‐depth spectroscopic investigations, and theoretical calculations showed that the dangling thiol groups promote noncovalent interactions with CO_2_ reduction intermediates, increasing the catalytic efficiency.

Defect engineering provides another effective approach for enhancing photocatalytic CO_2_ reduction using a QD photocatalyst. Defect manipulation creates unsaturated metal coordination sites, which not only tune the optoelectronic properties of the material but also act as redox‐active centers promoting catalytic activity. Additionally, defect–defect interactions affect both charge carrier and catalytic dynamics. Mengke Cai et al. reported defect‐engineered Zn‐doped CuInS_2_ (ZCIS) QDs in which copper vacancies and Cu^2+^ defect states work synergistically to promote CO_2_ reduction (Figure 14a–f) [265]. Optical and transient spectroscopic investigations revealed that copper vacancies modulate the distribution of the Cu^+^ and Cu^2+^ defect states. The Cu^+^‐related defect states suppress interband absorption and mitigate Shockley–Read–Hall recombination, whereas Cu^2+^‐related states contribute to prolonged exciton lifetimes in the QDs. In situ infrared spectroscopy and density functional theory calculations showed that Cu^2+^ defect sites near copper vacancies facilitate the activation of CO_2_ to form COOH* intermediates. As a result, the catalyst achieved a CO production rate of 532.3 μmol g^−1^ h^−1^ with a turnover number of ~1963 after prolonged illumination. In addition to defect engineering, compositional modification has also been explored to enhance the photocatalytic performance of QDs. Zhou et al. introduced rare‐earth ytterbium (Yb) into CuInS_2_ QDs and coupled them with two‐dimensional g‐C_3_N_4_ nanosheets to form a hybrid photocatalyst (Figure 14g–l) [266]. The incorporation of Yb alters the electronic structure of CuInS_2_, creating additional energy levels that facilitate efficient electron transfer during photocatalysis. The hybrid system achieved a CO_2_‐to‐CH_4_ conversion rate of 41.47 μmol g^−1^ h^−1^ with an appealing selectivity of 94.3%. Mechanistic studies revealed that Yb doping promotes preferential CO_2_ adsorption and lowers the activation barrier for intermediate formation, thereby accelerating reaction kinetics and enhancing product selectivity.

**FIGURE 14:**
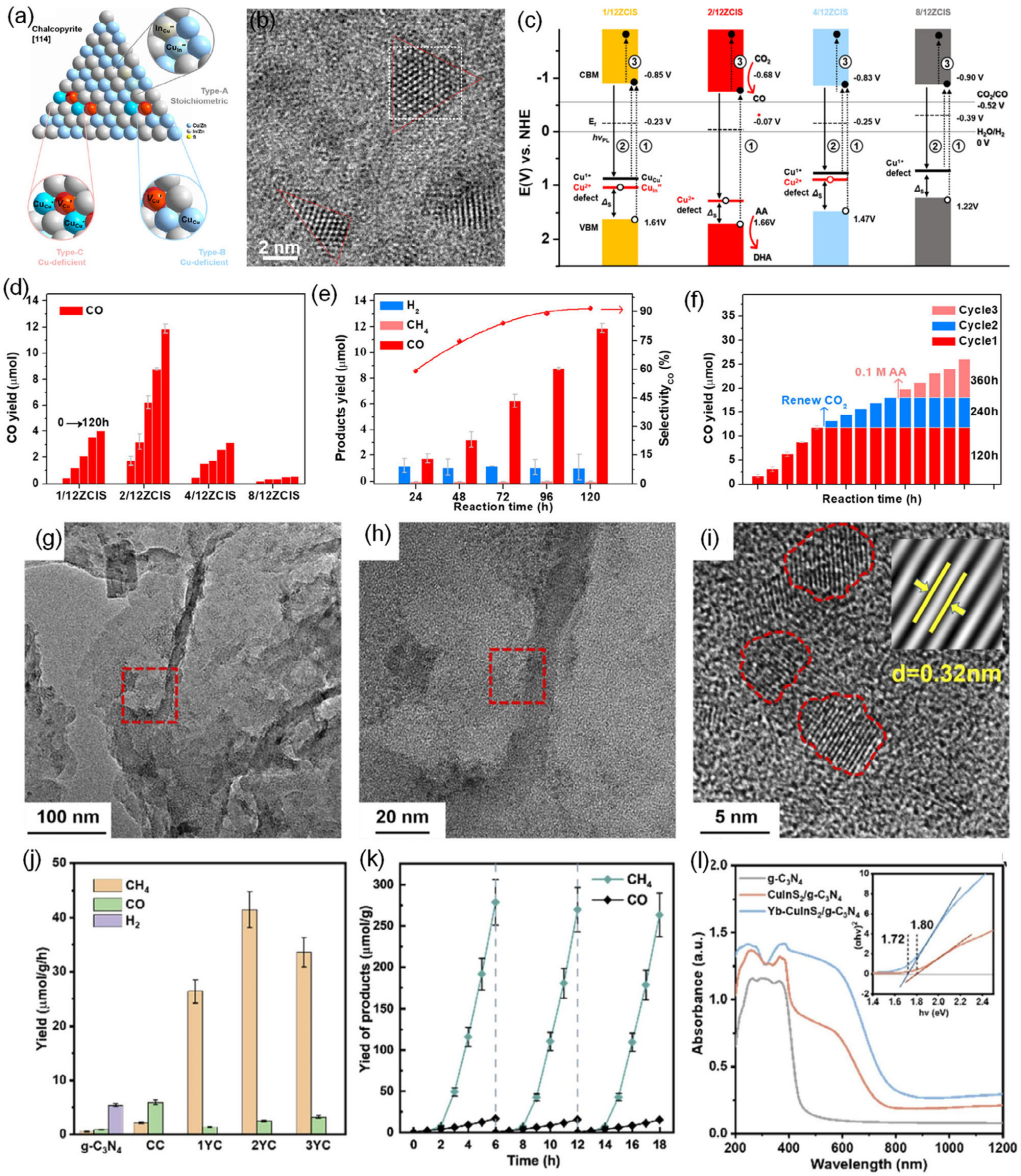
(a) Schematic diagram of different defect state types in stoichiometric and Cu‐deficient Zn‐doped CuInS_2_ (ZCIS) QDs. (b) TEM image of ZCIS QDs. (c) Band energy diagram of 1/12 ZCIS, 2/12 ZCIS, 4/12 ZCIS, and 8/12 ZCIS. (d) CO_2_ reduction to CO yield under simulated solar illumination of different ZCIS QDs. (e) CO_2_ reduction product distribution of 2/12ZCIS QDs. (f) Photocatalytic stability test of 2/12ZCIS QDs. Reproduced with permission from ref. [265]. Copyright 2023, American Chemical Society. (g, h) TEM and (i) HRTEM images of the Yb‐CuInS_2_/g‐C_3_N_4_ heterostructure. (j) Photocatalytic CO_2_ reduction test, (k) recyclability test, and (l) UV–vis absorption spectra of g‐C_3_N_4_, CuInS_2_/g‐C_3_N_4_, and Yb‐CuInS_2_/g‐C_3_N_4_. Reproduced with permission from ref. [266]. Copyright 2025, Elsevier.

Overall, these studies highlight the importance of rational catalyst design, including surface ligand engineering, defect modulation, heterostructure construction, and compositional tuning, in improving the efficiency and selectivity of photocatalytic CO_2_ reduction. Continued progress in understanding charge‐transfer processes and reaction intermediates will be essential for developing highly efficient solar‐driven systems for sustainable CO_2_ conversion.

#### 4.4.4 Photocatalytic Pollutant Degradation and Bacteria Disinfection

Concerns about the sustainability and preservation of water resources have grown as a result of rapid economic and population growth. Nearly two million tons of waste pollutants are carelessly dumped into natural water systems every day, seriously impairing water quality and endangering human health, which ultimately results in a high death rate globally [267]. The most common and dangerous contaminants are antibiotic compounds, monoaromatic hydrocarbons, and leftover toxic dyes that mostly come from industrial effluents. Water pollution has become a major worldwide concern as a persistent and complicated environmental issue. To reduce contamination of water resources, a variety of wastewater treatment techniques have been developed and put into practice, including ion‐exchange procedures, adsorption techniques, membrane separation technologies, and electrochemical purification [268]. These traditional methods still have a number of drawbacks despite significant advancements, such as limited adsorption capacity, quick activity loss, the production of secondary pollutants, and high energy requirements. Photo‐induced catalytic degradation has recently drawn a lot of attention as a viable and sustainable method for treating wastewater in response to these difficulties [269].

Semiconductor QDs have garnered significant interest in recent years as effective photocatalysts because of their enhanced charge separation capabilities, strong light absorption, and tunable band structures. Ag_2_S QDs have demonstrated an encouraging potential in environmental remediation. For example, Zhao et al. described the creation of Ag_2_S QDs anchored on SnS_2_ nanoflowers using an in situ ion‐exchange method to create a heterojunction photocatalyst [270]. The resulting Ag_2_S@SnS_2_ composite exhibited flower‐like structures (~1.5 μm) with good crystallinity and a bandgap similar to pristine SnS_2_. Notably, the optimized 3% Ag_2_S@SnS_2_ composite demonstrated significantly enhanced photocatalytic degradation of Rhodamine B under simulated sunlight irradiation, along with excellent cycling stability. The improved performance originated from the effective separation of photogenerated electron–hole pairs, while hydroxyl radicals and photogenerated holes were identified as the dominant reactive species in the degradation process. Beyond conventional semiconductor systems, Ag_2_S QDs have also been integrated with biological systems to create innovative photocatalytic platforms. Sun et al. developed a whole‐cell biohybrid catalyst by in situ synthesizing Ag_2_S QDs on the surface of engineered Escherichia coli (E. coli) cells (Figure 15) [271]. This biohybrid system exhibited superior visible‐light‐driven photocatalytic degradation of organic pollutants compared to individual components. Mechanistic studies revealed that superoxide radicals (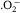) generated during photocatalysis played a key role in pollutant degradation. The enhanced performance was mainly attributed to efficient interfacial charge separation and transfer between the bacterial cells and the Ag_2_S QDs. Such biohybrid strategies offer a promising pathway for sustainable bioremediation and can potentially be extended to other microorganisms, such as Bacillus or yeast.

**FIGURE 15:**
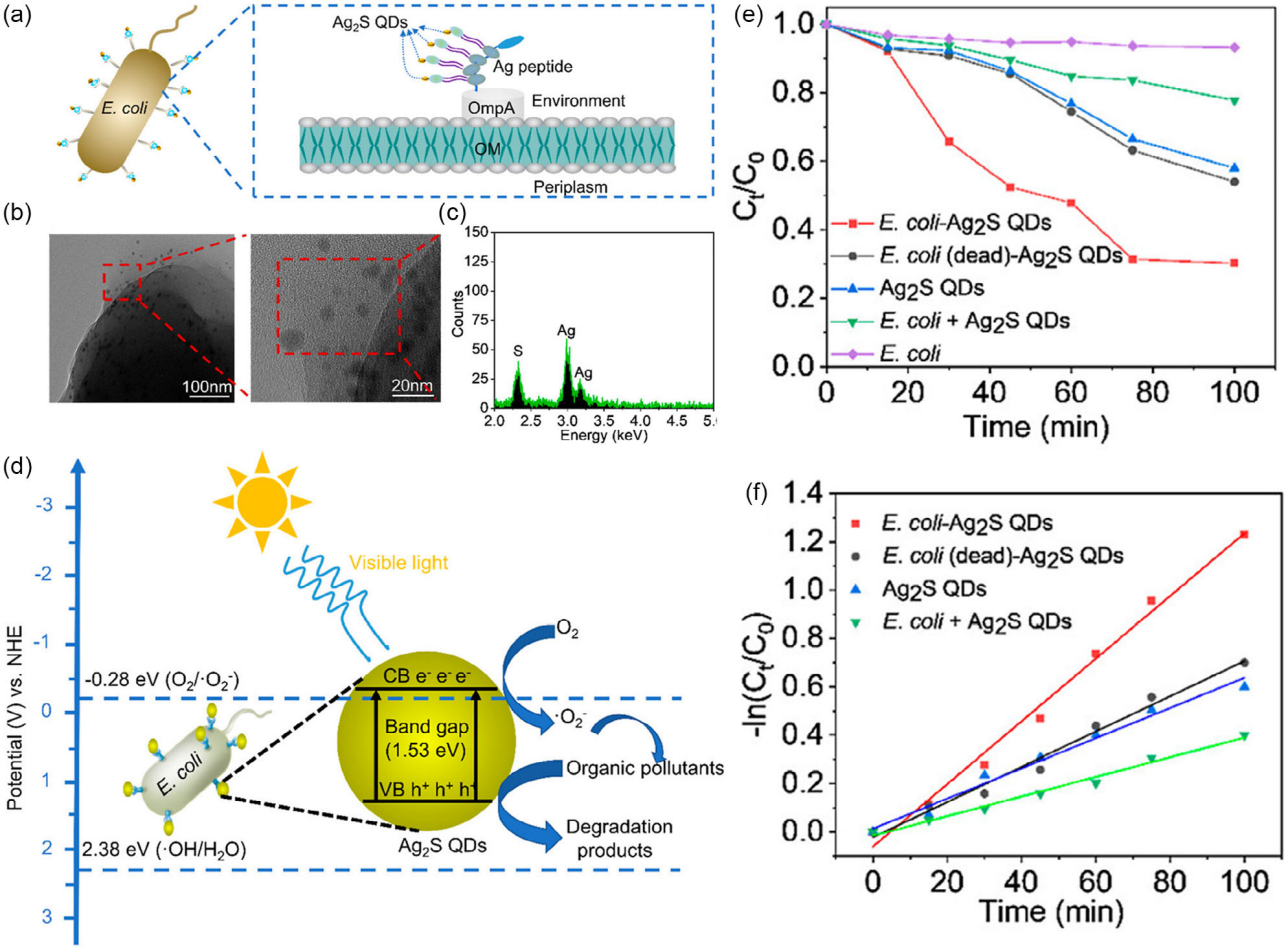
(a) Ag‐binding peptide on the surface of an engineered *E. coli* cell by the outer membrane protein OmpA. (b) TEM images of Ag_2_S QDs on *E. coli* cells. (c) EDX elemental analysis of Ag_2_S QDs. (d) Band energy diagram of Ag_2_S QDs. (e) Photocatalytic MB degradation by the *E. coli*‐Ag_2_S QD biohybrid system under visible light and corresponding (f) kinetic fit curves [271]. Reproduced with permission from ref. [271]. Copyright 2022, American Chemical Society.

Ag_2_S QDs have shown notable antimicrobial and photocatalytic properties in addition to degrading pollutants. Ag_2_S QDs with particle sizes between 8 and 9 nm were created by Sharma et al. using an aqueous method [272]. The produced QDs demonstrated potent antifungal activity against Alternaria alternata and Rosellinia, in addition to strong antibacterial activity against Staphylococcus aureus, Klebsiella pneumoniae, and E. coli. Additionally, using just 5 mg of the catalyst in a 100 mL solution, the QDs demonstrated outstanding photocatalytic performance in the degradation of methylene blue dye, achieving over 94% degradation in 120 min. Studies on radical scavenging verified that a variety of reactive species, such as superoxide and hydroxyl radicals, are involved in the photocatalytic process. Ag_2_S QDs have also been investigated for photocatalytic bacterial disinfection. Sun et al. created biocompatible Ag_2_S QDs with strong fluorescence in the second near‐infrared (NIR‐II) region by employing a peptide‐mediated synthesis method with a biomimetic silver‐binding peptide (AgBP_2_) [273]. These AgBP_2_‐capped Ag_2_S QDs demonstrated excellent photothermal and photocatalytic properties under 808 nm NIR laser irradiation, generating reactive oxygen species that effectively inactivated bacteria. As a result, the system achieved a disinfection efficiency of over 99% against E. coli within 25 min. The enhanced antibacterial performance was attributed to the synergistic effects of ROS‐mediated photocatalysis and photothermal heating.

#### 4.4.5 Photocatalytic Nitrate (NO_3_^−^) Removal

Nitrate ions are particularly difficult to remove from water because they are highly soluble and chemically stable in aqueous media. The World Health Organisation advises a maximum allowable concentration of 10 ppm of nitrate‐nitrogen in drinking water because high nitrate levels are detrimental to human health [274]. In photocatalytic nitrate reduction, NO_3_^−^ is typically reduced to NO_2_^−^ and then further reduced to nitrogen radicals ($N^{.}$), which then couple to form molecular nitrogen (N_2_). Additionally, QDs can be used as a photocatalyst to extract NO_3_^−^ from water.

CuInS_2_ (CIS) has been shown by Yue et al. to be an effective visible‐light photocatalyst for nitrate removal [274]. Under visible light irradiation (*λ* > 400 nm), the catalyst achieved a nitrate conversion rate of 8.32 mg N h^−1^ when co‐loaded with noble metals like Pt, Ru, and Au. Notably, in a matter of hours, highly concentrated nitrate solutions (100 ppm N) were entirely transformed into innocuous N_2_ without producing unwanted byproducts like NH_3_ or H_2_. With an apparent quantum yield of 23.85%, the photocatalyst was most active at 500 nm. The observation that the presence of competing halide ions decreased the catalytic efficiency supported the adsorption–reduction pathway as the reaction mechanism. Furthermore, sacrificial agents were needed for the process, even though the catalyst was still effective with a number of safe substitutes. Wang et al. tackled the problem of obtaining effective photocatalytic nitrate reduction without the use of external hole scavengers in a different study [275]. They developed a composite catalyst by anchoring ZnS QDs onto polyaniline‐coated corn straw (ZnS QDs‐PANI‐CS). The optimized catalyst (0.8% ZPC) exhibited a nitrate removal efficiency of 98.4% with a N_2_ selectivity of 96.8%, even in the absence of added sacrificial agents. The superior performance was attributed to enhanced electron mobility and prolonged charge carrier lifetimes. Furthermore, ZnS QDs promoted the transformation of the quinone structure of polyaniline into a benzene‐like structure, improving the reductive capability of the catalyst. The generation of formic acid from surface functional groups facilitated the formation of ${\mathrm{CO}}_{2}^{.-}$ radicals, which actively participated in nitrate reduction. In addition, ZnS QDs increased the density of Lewis acid sites, improving the N_2_ selectivity, while the formation of N─Zn bonds enhanced the structural stability of the composite. This strategy demonstrates a promising approach for designing efficient photocatalysts for nitrate removal in wastewater treatment.

Overall, these studies demonstrate that QDs are highly effective photocatalysts for nitrate reduction due to their tunable electronic structure, strong light absorption, and efficient charge separation properties. Their ability to selectively convert nitrate into environmentally benign N_2_ highlights their potential for sustainable water purification technologies. Moreover, owing to their unique surface reactivity and controllable band structures, QDs‐based photocatalysts also hold significant promise for future solar‐driven nitrogen fixation processes, enabling the conversion of N_2_ and NO_3_^−^ into value‐added products such as NH_3_.

## 5 Challenges and Outlook

In summary, we provided an overview of the intriguing properties of HMF chalcogenide NCs as new semiconductors for future optoelectronics and photocatalysis. Over the past decade, significant progress has been made in the performance of these materials. The various publications and studies show that the performance of this material class is not, *per se*, inferior to HMB materials. Nevertheless, several important challenges and open questions remain for HMF nanocrystals to render them a technologically viable semiconductor platform. Importantly, these challenges are particularly pronounced for ternary and multinary chalcogenide NCs, where stoichiometry, cation distribution, and structural disorder are strongly coupled to their optical and electronic properties.

The synthesis of heavy metal‐free nanocrystals has made considerable progress in recent years, and a variety of synthetic routes have been identified that allow for tuning and controlling the material composition, structure, and size of the nanocrystals. Nevertheless, it has become apparent that direct synthesis methods, such as the frequently used hot‐injection method, are sometimes impractical, especially for nanocrystals consisting of three or more elements. As the number of constituent elements increases, differences in precursor reactivity, nucleation kinetics, and incorporation rates can lead to deviations from the nominal stoichiometry and compositional heterogeneity between individual NCs. While many of these complex HMF chalcogenide NCs can be produced via cation‐ and anion‐exchange reactions, precise control over the final elemental composition and distribution in these reactions is often difficult to achieve. Incomplete or spatially nonuniform ion exchange can further generate compositional gradients and local structural disorder, which may substantially alter the electronic structure and optical response. Many studies of HMF chalcogenide NCs showed that the structural order, i.e., the precise position and relative distribution of cations and anions within the crystal lattice, significantly influences the optical and electronic properties of NCs. Consequently, the nominal chemical formula alone may not fully describe the optoelectronic behavior of multinary HMF chalcogenide NCs as local cation arrangements, vacancies, antisite defects, and compositional fluctuations can introduce localized or band‐tail states and modify carrier recombination and transport. To tackle the challenge of structural disorder in these materials, additional insights into the exact nature, formation, localization, and mitigation of these defects have to be obtained. Determining the distribution of individual elements experimentally within nanocrystals can be extremely challenging due to their very small size and a certain degree of heterogeneity among them. While microscopic methods such as HRTEM, HAADF–STEM, or EDX offer the best opportunity to examine individual nanocrystals with atomic resolution, they can also often affect the distribution themselves. Spectroscopic methods or X‐ray diffraction, on the other hand, frequently examine nanocrystal ensembles, and information about the exact elemental distribution in single nanocrystals can often only be determined indirectly. In our opinion, the greatest potential to tackle these challenges lies in a combination of theory and experimental investigations to derive a comprehensive understanding of structural disorder in HMF chalcogenide NCs, which will lead to the development of new solutions and strategies to control and reproducibly adjust the desired elemental distribution, optimized for the individual application.

Because the NCs’ properties correlate especially strongly not only with size but also with the elemental composition, future developments of highly precise synthetic approaches with high compositional control are extremely important. In particular, controlling the relationship between stoichiometry, cation distribution, and defect chemistry should be considered a central objective for achieving reproducible optical and electronic properties. Novel reagents, precursors, and synthesis strategies for this class of nanocrystals could lead to a breakthrough here. Particular emphasis should be given to the scalability of the chemical syntheses, which is a basic requirement for widespread industrial application of these materials in optoelectronics, sensors, or catalysis. Classical synthesis methods, such as batch‐wise hot‐injection methods routinely employed in the laboratory environment, are generally insufficient for producing materials on a kilogram scale, as required, for example, for applications in PVs or photocatalysis. Further creative research is needed to develop scalable syntheses that can be easily transferred to an industrial scale without compromising the material properties, composition, or quality of the nanocrystals. Moreover, such syntheses should be highly reproducible, a challenge that has received little attention in the field. Obviously, such syntheses must also be energy‐ and resource‐efficient as this is the only way to achieve cost efficiency to enter the market. To facilitate the widespread use of HMF chalcogenide NCs in the future, it is essential to examine the extent to which these materials can be reused and recycled. Currently, very few studies are focusing on the extraction and separation of these nanocrystals. Particularly concerning the efficient use of natural resources and the often‐used critical raw materials, such as indium, bismuth, and other elements in HMF chalcogenide NCs, it is crucial to establish the recyclability of these materials early on to build a truly sustainable circular economy for HMF nanocrystals.

The main challenge for HMF chalcogenide NCs undoubtedly lies in their designation as a highly environmentally compatible material class with low‐to‐no toxicity. Numerous nanocrystal compounds and compositions are hastily labeled “nontoxic” or “environmentally friendly” alternatives as soon as they do not contain Pb, Cd, or Hg. However, while the absence of such elements with high acute toxicity is promising at first glance, the toxicological properties of the various material compositions are often far less investigated and sometimes unknown. The interaction of materials in the environment and in biological systems can be extremely complex, and risks and adverse effects are often only revealed after decades of use and repeated exposure. Furthermore, the hazards and physiological effects of HMF material compositions can be very different when the material exists in a nanoscopic form. The nanocrystal size alone poses certain risks as such small objects can penetrate cell membranes or enter the bloodstream. For example, after decades of widespread and often unsupervised use, significant concerns have arisen in recent years in the case of titanium dioxide and silicon dioxide nanoparticles, which are nontoxic as coarse powders. It has recently been shown that small SiO_2_ nanoparticles (<100 nm) exhibit genetic and epigenetic toxicity [276], and their use in a variety of consumer products is becoming a major health problem. Nanocrystals possess a large surface area, which offers high reactivity. They can interact with environmental factors or biological substances, cells, or tissue, rendering the reactivity of the nanocrystals different from that of larger particles of the same material. Additionally, dynamic internal processes such as Ag^+^ migration can lead to structural degradation and the unintended leaching of free silver ions over time, drastically altering the long‐term environmental footprint and physiological toxicity of otherwise “safe” compositions. Moreover, HMF chalcogenide NCs are typically passivated by ligands, which themselves are often highly coordinating or redox‐active and can exhibit their own toxicity. As future semiconductors, HMF chalcogenide NCs offer a way to enable RoHS‐compliant electronics; however, their toxicity, environmental compatibility, and the effects of long‐term exposure still need to be thoroughly investigated.

## Funding

This study was supported by HORIZON EUROPE Excellent Science (grant no. 101154567) and the DFG Cluster of Excellence (EXC 3035, Project‐ID 533607596).

## Conflicts of Interest

The authors declare no conflicts of interest.

## Data Availability

The data that support the findings of this study are available on request from the corresponding author. The data are not publicly available due to privacy or ethical restrictions.
